# Supplementary material for: Trends in non-pharmaceutical intervention (NPI) related community practice for the prevention of COVID-19 in Addis Ababa, Ethiopia
Source: PLoS One. 2021 Nov 23;16(11):e0259229. doi: 10.1371/journal.pone.0259229 (PMC8610281; doi:10.1371/journal.pone.0259229)
Supplement: S1 File — (DOCX) [file pone.0259229.s001.docx]

**Monitoring of non-pharmaceutical Intervention (NPI) community practice for the prevention of Covid-19 in Addis Ababa, Ethiopia**

**Rapid assessment of the progress of universal key interventions**

**Standardizing the Observation Method**

**Investigators: Damen Hailemariam (Primary PI), Abera Kumie Co-PI), Samson Wakuma, Yifoker Tefera, Teferi Abegaz, Worku Teferra, Wondimu Ayele, Mulugeta Tamire**

**School of Public Health,**

**College of Health Sciences**

**Addis Ababa University**

**27 May 2020 Version 1**

# Objectives

## *General Objective*

This study is designed to monitor the trend and pattern of COVID-19 preventive behavior among residents within selected area of Addis Ababa City.

## *Specific Objectives*

1. To assess the pattern of hand hygiene practice;
2. To assess the pattern of use of face masks;
3. To assess the practice of keeping physical distance; and
4. Describe the trends in the pattern of the above preventive behavior within three months (June to August 2020.
5. Characterize the observed areas by risk groups

### **Frequency of data collection and observation**

Observation data collection will be made twice a week (Wednesdays and Sundays). All study sites will be observed on Wednesdays. In addition, those community institutions (religious institutions, open markets, transport sites, streets, and food establishments) will also be observed Sunday.

### **Time of observation**

The time of observation must fit the time of peak hours of services at the respective sites. We learned the time observation from the pilot study. This time must be that has an increased risk of covid-19 transmission when service seekers get close each other. Proposed time schedule is as follows.

1. Religious paces: Normally 6 – 7 am in the week day and 6-8 am in the weekend day, sometimes during ‘Kibrebeal’ you may observe 10-12 am
2. Workplaces: Preferably in the morning 7-9 am or in the afternoon 13-14 pm
3. Health facility: Preferably in the morning 8-10 am
4. Bank: Preferably 8-10 am or 13-14 pm
5. Transport: Preferably 7-10 or 15-17 pm
6. Open market place: Preferably 10-12 am or 16-18 pm
7. Food and drink establishment: Preferably 12-14 pm
8. Street crossing site: (“Zebra” site): Preferably 8-9 am or 12-14 or 16-17pm

## *Standardization of Observations*

To standardize the observations, the following items have been fixed:

- The time of observation is that time fitting the peak of service of the respective sites. This time is fixed for each site to be observed all across the sub cities.
- The time of observation is consistent for each day of data collection.
- The site to be observed is fixed and will be similar for every day of data collection.
- The point of observing the individual at each site is fixed in each day of data collection.

The physical context of observing an institute should be well studied before launching data collection. This is very helpful to fix the necessary standards (peak hour, peak time, observation point) to collect data consistently all the time. Figure 1 shows some of the contexts.


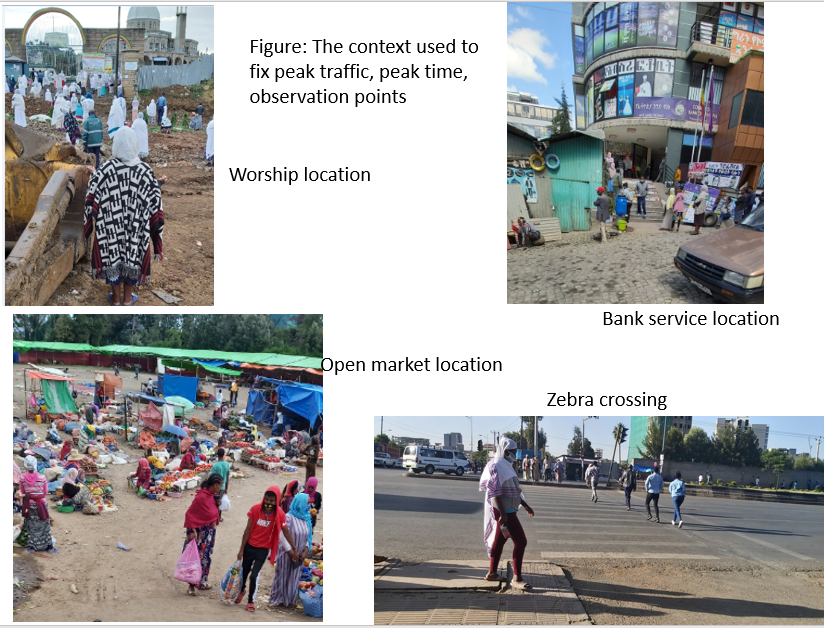


Figure 1: **The context used to fix peak human traffic, peak time, observation points**

## Data quality assurance

The data quality mainly depends on the nature of observation and individual to pick the right practice at a given moment. We had structured, pre-tested, and piloted data collection checklist. The duration of to observe the practice in one site is fixed at a minimum of 15-30 minutes. The observation process is standardized in terms of time of and site of observations across all data collectors during training sessions. The first data of observation was supervised with a view of checking the standard observation. There will be periodical supervision to cover the status of performance. The data managing using ODK provides quick access for looking for any error that can be corrected for subsequent days.

# Annex 4: Data collection procedure: Standardizing the observation

- Collect 10 observation per site
- There must be a plan of action that has the list of 8 sites by observation time accommodating peak hour services.
- Data will be collected by observation of the intended practice (hand hygiene, respiratory hygiene, and physical distance) that are advised or subjected for re-enforcement
- Data will be collected by observing the practice of individuals for a period of about 15-30 minutes at each service provision facility or location. The time of observation may vary depending on what is observed.
- Data will be collected in the middle of the week on **Wednesday’s** and week end Sunday’s
- Individual COVID-19 prevention practice for (Hand hygiene, physical distancing and Respiratory hygiene) will be recorded during the observation period
- The data collector should be in an appropriate position to observe the practice, preferably closer to the facility gate, while trying not to be observed by others for reasons of ethical issues.
- The observation facility should be selected based on reasonable number of people using the service or the location for a service
- The data collectors should start observation of the new comer to the church to avoid observation selection bias
- Care should be taken, the purpose of data collection about physical distancing is to measure the practice of a person maintaining distance with the unknown person in the community; hence the observed person should be non-relatives or families, who are naturally closer because of knowing each other status, specially observation on streets, churches, health facilities, restaurants.

**Data collection sites Fixing (hot spots)**

1. **Religious places:** This include Orthodox church, Mosques and Protestant churches
2. **Market places**: This includes open markets and supermarkets
3. **Transport services**: This includes train, bus and taxi stops
4. **Streets**: This includes a street crossing site called “zebra” that is closer to a traffic light or roundabout or footbridge. We may include as well high public gathering places, shoe polishing and street shopping
5. **Ban**ks: This include primarily CBE. It may private banks including ATM machine outlets
6. **Food and drink establishments**: This includes hotels, restaurants, cafeterias and bars where there is a good flow
7. **Health facilities**: This includes public health facilities. It may include private health institutions based on the flow of patients.
8. **Workplaces**: This includes formal business employed more than 10 workers: public or private
