# Supplementary material for: Trends in non-pharmaceutical intervention (NPI) related community practice for the prevention of COVID-19 in Addis Ababa, Ethiopia
Source: PLoS One. 2021 Nov 23;16(11):e0259229. doi: 10.1371/journal.pone.0259229 (PMC8610281; doi:10.1371/journal.pone.0259229)
Supplement: S2 File — (DOCX) [file pone.0259229.s002.docx]

# Annex 1: COVID-19 Prevention Practices Data Collection Tool in a community

Observational data collection forms – Community practice

Observation location/service facility related information

1. City name: ___________________________________________________
2. Sub-city name: ________________________________________________
3. Location name (ex, Koteb, Sarbet, etc): ____________________________
4. Type of service/facility (ex, Bank, Street, etc): _______________________
5. Specific type of service facility (ex, CBE, restaurant, etc): ______________
6. Observer name: _______________________________________________
7. Date of observation: ___________________________________________
8. Time of observation: Start time_____________ end time _____________
9. Comment (any additional observation):

______________________________________________________________

**Individual or group level practice observation form**

| **ID** | **Sex** | **Age group** | | **Hand hygiene** | **Physical distance** | | **Respiratory hygiene** | | **Incidence of cough or sneezing** | | **Comment** |
| --- | --- | --- | --- | --- | --- | --- | --- | --- | --- | --- | --- |
| 1 |  |  | |  |  | |  | |  | |  |
| 2 |  |  | |  |  | |  | |  | |  |
| 3 |  |  | |  |  | |  | |  | |  |
| 4 |  |  | |  |  | |  | |  | |  |
| 5 |  |  | |  |  | |  | |  | |  |
| 6 |  |  | |  |  | |  | |  | |  |
| 7 |  |  | |  |  | |  | |  | |  |
| 8 |  |  | |  |  | |  | |  | |  |
| 9 |  |  | |  |  | |  | |  | |  |
| 10 |  |  | |  |  | |  | |  | |  |
| 11 |  |  | |  |  | |  | |  | |  |
| 12 |  |  | |  |  | |  | |  | |  |
| **ID** **code**: Continuous number representing individuals  **Hand hygiene code:**   1. Proper hand hygiene 2. Improper hand hygiene 3. No hand hygiene | | | **Sex code:**   1. Male 2. Female   **Age group code:**   1. < 18 years old 2. 18-50 years old 3. > 50 years old | | | **Physical distance code:**   1. Proper physical distance 2. Improper physical distance 3. No physical distance | | **Respiratory hygiene code:**   1. Proper respiratory hygiene 2. Improper respiratory hygiene 3. No respiratory hygiene | | **Incidence of cough or sneezing code:**   1. Yes 2. No   9-None observable | |

**Operational definitions**

1. **Hand washing:**
   1. **Proper hand washing practice**: A person wash hands (outer and inner, fingers) with available detergent and water at for 20-30 seconds getting in the facility or taking the services
   2. **Improper hand washing practice**: A person wash hands only with water or water and detergent for less than 20 seconds before getting in the facility or taking the services.
   3. **No hand washing practice**: A person getting in to the facility or taking the services without washing hands or use of sanitizer
2. **Hand sanitizing**: use of sanitizers such as alcohol to rub all surfaces (outer and inner, fingers) of the two hands for about 20 seconds.
   1. **Proper hand sanitizing practice**: A person wash hands (outer and inner, fingers) with available detergent and water at for 20 seconds getting in the facility or taking the services
   2. **Improper hand sanitizing practice**: A person wash hands with alcohol less than 20 seconds before getting in the facility or taking the services
   3. **No hand washing practice**: A person getting in to the facility or taking the services without washing hands or use of sanitizer
3. **Social or physical distance:**
   1. **Proper social or physical distance**: A person is keeping 1 meter away from another person during getting service or greetings or shopping or discussing or praying; and 2 m from any person who is coughing or sneezing.
   2. **Improper** **social or physical distance**: A person is keeping less than 1 meter away with another person during getting service or greetings or shopping or discussing or praying
   3. **No** **social or physical distance**: A person has body contacts with another person during getting service or greetings or shopping or discussing or praying
4. **Respiratory hygiene:**
   1. **Proper respiratory hygiene**: A person cover the mouth and nose with mask or any type of cloth in any of the activity or handkerchief or clothes or tissue or hands in 1 meter away from the other person (Mark in the comment mask or cloth)
   2. **Improper respiratory hygiene**:
      - 1. A person covering only the mouth or nose with mask or clothes OR
        2. A person do not covering mouth and nose with hands or cloths while coughing or sneezing OR a person has properly using mask but observed to touch his eye/s a person has mask covering the mouth but not the nose or vice versa
        3. A person covering both nose and mouth with mask but touch the mask with hands or touch eyes
   3. **No respiratory hygiene**: A person do not cover the mouth and nose.
