# Supplementary material for: Trends in non-pharmaceutical intervention (NPI) related community practice for the prevention of COVID-19 in Addis Ababa, Ethiopia
Source: PLoS One. 2021 Nov 23;16(11):e0259229. doi: 10.1371/journal.pone.0259229 (PMC8610281; doi:10.1371/journal.pone.0259229)
Supplement: S4 File — (PDF) [file pone.0259229.s004.pdf]

ADDIS ABABA UNIVERSITY, COLLEGE OF HEALTH SCIENCES (IRB)
አዲስ አበባ ዩኒቨርስቲ ጤናሳይንስ ኮሌጅ
Institutional Review Board

ANNEX 3
Form AAUMF 03-008

IRB's Decision

Emergency meeting No: 07/2020 Emergency Meeting Date: June 08, 2020
Protocol number: 049/20/SPH

**Protocol Title:** A proposal on monitoring of non-pharmaceutical Intervention (NPI) community practice for the prevention of Covid-19 in Addis Ababa, Ethiopia Rapid assessment of the progress of universal key interventions		
Principal Investigator:	Professor Damen Haile Mariam	
Institute:	College of Health Sciences, AAU	
Elements Reviewed (AAUMF 01-008)	☑ Attached	☐ Not attached
Review of Revised Application<br>☐ Yes ☐ No	Date of Previous review:	
Decision of the meeting:	☑Approved ☐Approved with Recommendation<br>☐Resubmission ☐ Disapproved	

I. Elements approved-
1. Protocol Version No: 2
2. Protocol Version Date:
3. Informed consent Version No. 2
4. Informed Consent Version Date:

II. Obligations of the PI-
1. Should comply with the standard international & national scientific and ethical guidelines
2. All amendments and changes made in protocol and consent form needs IRB approval
3. The PI should report SAE within 10 days of the event
4. End of the study, including manuscripts and thesis works should be reported to the IRB
5. The PI should report non-compliance and unanticipated events

III. TO NERC ☐

Institution Review Board (IRB) Approval: Period from: June 17, 2020 to June 16, 2021
Follow up report expected in

3 Months ✓ 6 months ___ 9 months _____ one year ____

Chairperson, IRB
Dr. Adamu Addissie

Signature ____________
Date: 17/06/2020

አዲስ አበባ ዩኒቨርስቲ
Institutional Review Board Office
(IRB)
Faculty of Medicine
ADDIS ABABA UNIVERSITY

progress report every 3 M.
