# Supplementary material for: Trends in non-pharmaceutical intervention (NPI) related community practice for the prevention of COVID-19 in Addis Ababa, Ethiopia
Source: PLoS One. 2021 Nov 23;16(11):e0259229. doi: 10.1371/journal.pone.0259229 (PMC8610281; doi:10.1371/journal.pone.0259229)
Supplement: Attachment — Submitted filename: PONE-D-21-03031_reviewer.pdf [file pone.0259229.s005.pdf]

PLOS ONE

Trends in non-pharmaceutical intervention (NPI) related community practice for the prevention of COVID-19 in Addis Ababa, Ethiopia

--Manuscript Draft--

**Manuscript Number:**	PONE-D-21-03031
**Article Type:**	Research Article
**Full Title:**	Trends in non-pharmaceutical intervention (NPI) related community practice for the prevention of COVID-19 in Addis Ababa, Ethiopia
**Short Title:**	Community practice for the prevention of Covid-19
**Corresponding Author:**	Yifoker Tefera<br>Addis Ababa University<br>aDDIS ABABA, ETHIOPIA ETHIOPIA
**Keywords:**	COVID-19; non-pharmaceutical intervention; community practice; COVID-19 prevention
**Abstract:**	Background<br><br>The COVID-19 pandemic has affected Ethiopia since March 13, 2020, when the first case was detected. Since then, the incidence of cases continues to increase day by day. The health sector has recommended a set of universal preventive measures to be practiced by the community. However, studies on adherence to these preventive measures is limited to inform evidence-based policy making.<br><br>Objective<br><br>To rapidly appraise and monitor the preventive behavior of the population by conducting a series of observations on implementing the prescribed measures at selected sites within the city of Addis Ababa.<br><br>Methods<br><br>A repeated series of cross-sectional non-participatory observations for the preventive behaviors related to hand washing, physical distancing, and respiratory hygiene practices was done for the period of April 24-June 28/2020. Data were collected using Open Data Kit (ODK) tool in ten major sites involving each 8 public facilities targeted for individual observations. There were ten individuals who observed at each facility over two days in a week at peak hours of client's service. One week day and one weekend day were considered in order to observe a weighted outcome of the study. WHO operational definitions of the preventive behaviors were adopted for this study. Observations were fixed at gates and or entrances of public service providing facilities including health facilities, workplaces, banks, food establishments, religious institutions, public transport waiting areas, street crossing sites and market places.<br><br>Results<br><br>A total 12056 individual observations with 52% males, and 82% in 18-50 years old age group were involved in this study. The results showed a sharp rise in the practice of respiratory hygiene (from 14% in week one to 77% in week 10), while those of hand hygiene (from 24.2% in week one to 23.7% in week 10) and physical distancing (from 34.4% in week one to 35.2% in week eight) changed little during the last weeks of observation. The three combined practices of hand and respiratory hygiene and physical distancing at individual level increased at a slow pace from 4.7% in week one to 8.1% in week 10.<br><br>Conclusion<br><br>As adherence by the public to the preventive measures seems to be higher among those with relatively better awareness and during times of government mandates, mass education. The preventive measures need to be comprehensive with strict

Powered by Editorial Manager® and ProduXion Manager® from Aries Systems Corporation

	enforcement, as situations require.
Order of Authors:	Abera Kumie
	Damen Hailemariam
	Samson Wakuma
	Yifoker Tefera
	Teferi Abegaz
	Worku Tefera
	Mulugeta Tamire
	Wondimu Ayele
Additional Information:	
**Question**	**Response**
**Financial Disclosure**<br><br>Enter a financial disclosure statement that describes the sources of funding for the work included in this submission. Review the submission guidelines for detailed requirements. View published research articles from *PLOS ONE* for specific examples.<br><br>This statement is required for submission and **will appear in the published article** if the submission is accepted. Please make sure it is accurate.<br><br>**Unfunded studies**<br>Enter: *The author(s) received no specific funding for this work.*<br><br>**Funded studies**<br>Enter a statement with the following details:<br>• Initials of the authors who received each award<br>• Grant numbers awarded to each author<br>• The full name of each funder<br>• URL of each funder website<br>• Did the sponsors or funders play any role in the study design, data collection and analysis, decision to publish, or preparation of the manuscript?<br>• **NO** - Include this sentence at the end of your statement: *The funders had no role in study design, data collection and analysis, decision to publish, or preparation of the manuscript.*<br>• **YES** - Specify the role(s) played.<br><br>* typeset	The author(s) received no specific funding for this work.

**Competing Interests**<br><br>Use the instructions below to enter a competing interest statement for this submission. On behalf of all authors, disclose any competing interests that could be perceived to bias this work—acknowledging all financial support and any other relevant financial or non-financial competing interests.<br><br>This statement is required for submission and **will appear in the published article** if the submission is accepted. Please make sure it is accurate and that any funding sources listed in your Funding Information later in the submission form are also declared in your Financial Disclosure statement.<br><br>View published research articles from *PLOS ONE* for specific examples.<br><br>**NO authors have competing interests**<br><br>Enter: *The authors have declared that no competing interests exist.*<br><br>**Authors with competing interests**<br><br>Enter competing interest details beginning with this statement:<br><br>*I have read the journal's policy and the authors of this manuscript have the following competing interests: [insert competing interests here]*<br><br>* typeset	The authors have declared that no competing interests exist.
**Ethics Statement**<br><br>Enter an ethics statement for this submission. This statement is required if the study involved:<br><br>• Human participants<br>• Human specimens or tissue<br>• Vertebrate animals or cephalopods	Protocol No: 049/20/SPH<br><br>Institutional Review Board of the College of Health Sciences, Addis Ababa University

Powered by Editorial Manager® and ProduXion Manager® from Aries Systems Corporation

• Vertebrate embryos or tissues
• Field research

Write "N/A" if the submission does not require an ethics statement.

General guidance is provided below. Consult the submission guidelines for detailed instructions. **Make sure that all information entered here is included in the Methods section of the manuscript.**

Powered by Editorial Manager® and ProduXion Manager® from Aries Systems Corporation

**Format for specific study types**<br><br>**Human Subject Research (involving human participants and/or tissue)**<br>• Give the name of the institutional review board or ethics committee that approved the study<br>• Include the approval number and/or a statement indicating approval of this research<br>• Indicate the form of consent obtained (written/oral) or the reason that consent was not obtained (e.g. the data were analyzed anonymously)<br><br>**Animal Research (involving vertebrate animals, embryos or tissues)**<br>• Provide the name of the Institutional Animal Care and Use Committee (IACUC) or other relevant ethics board that reviewed the study protocol, and indicate whether they approved this research or granted a formal waiver of ethical approval<br>• Include an approval number if one was obtained<br>• If the study involved *non-human primates*, add *additional details* about animal welfare and steps taken to ameliorate suffering<br>• If anesthesia, euthanasia, or any kind of animal sacrifice is part of the study, include briefly which substances and/or methods were applied<br><br>**Field Research**<br><br>Include the following details if this study involves the collection of plant, animal, or other materials from a natural setting:<br>• Field permit number<br>• Name of the institution or relevant body that granted permission	
**Data Availability**<br><br>Authors are required to make all data underlying the findings described fully available, without restriction, and from the time of publication. PLOS allows rare exceptions to address legal and ethical concerns. See the PLOS Data Policy and FAQ for detailed information.	Yes - all data are fully available without restriction

A Data Availability Statement describing where the data can be found is required at submission. Your answers to this question constitute the Data Availability Statement and **will be published in the article**, if accepted.<br><br>**Important:** Stating 'data available on request from the author' is not sufficient. If your data are only available upon request, select 'No' for the first question and explain your exceptional situation in the text box.<br><br>Do the authors confirm that all data underlying the findings described in their manuscript are fully available without restriction?	
**Describe where the data may be found in full sentences. If you are copying our sample text, replace any instances of XXX with the appropriate details.**<br><br>• If the data are **held or will be held in a public repository**, include URLs, accession numbers or DOIs. If this information will only be available after acceptance, indicate this by ticking the box below. For example: *All XXX files are available from the XXX database (accession number(s) XXX, XXX.).*<br>• If the data are all contained **within the manuscript and/or Supporting Information files**, enter the following: *All relevant data are within the manuscript and its Supporting Information files.*<br>• If neither of these applies but you are able to provide **details of access elsewhere**, with or without limitations, please do so. For example:<br><br>*Data cannot be shared publicly because of [XXX]. Data are available from the XXX Institutional Data Access / Ethics Committee (contact via XXX) for researchers who meet the criteria for access to confidential data.*<br><br>*The data underlying the results presented in the study are available from (include the name of the third party*	All relevant data are within the manuscript and its Supporting Information files.

and contact information or URL).<br>• This text is appropriate if the data are owned by a third party and authors do not have permission to share the data.<br><br>* typeset	
Additional data availability information:	

Manuscript with text only
Click here to access/download;Manuscript;PBM5 Manuscript_Submitted_Plos_AK_Submitted_27Jan2021.docx

Original Article

Trends in non-pharmaceutical intervention (NPI) related community practice for the prevention of COVID-19 in Addis Ababa, Ethiopia

Authors List

Damen Haile Mariam[1], Abera Kumie[1], Samson Wakuma[1], Yifoker Teferra[1], Teferi Abegaz[1], Worku Tefera[1], Wendimu Ayele[1], Mulugeta Tamire[1]

[1] School of Public Health, College of Health Sciences, Addis Ababa University

Damen Mariam <damengoog@gmail.com>;
Samson Wakuma samson_wakuma@yahoo.com;
Yifo Tefera yifoomitu@yahoo.com;
Teferi Abegaz teferiabegaz@gmail.com;
Worku tefera <workutefera2000@yahoo.com>
Mulugeta Tamire <awonmuller@yahoo.com>
Wondimu Ayele <wondaay@gmail.com>

Corresponding Author

Abera Kumie[1]: aberakumie2@yahoo.com; Mobile +251911-882912

Abstract

Background: The COVID-19 pandemic has affected Ethiopia since March 13, 2020, when the first case was detected. Since then, the incidence of cases continues to increase day by day. The health sector has recommended a set of universal preventive measures to be practiced by the community. However, studies on adherence to these preventive measures is limited to inform evidence-based policy making.

Objective: To rapidly appraise and monitor the preventive behavior of the population by conducting a series of observations on implementing the prescribed measures at selected sites within the city of Addis Ababa.

Methods: A repeated series of cross-sectional non-participatory observations for the preventive behaviors related to hand washing, physical distancing, and respiratory hygiene practices was done for the period of April 24-June 28/2020. Data were collected using Open Data Kit (ODK) tool in ten major sites involving each 8 public facilities targeted for individual observations. There were ten individuals who observed at each facility over two days in a week at peak hours of client's service. One week day and one weekend day were considered in order to observe a weighted outcome of the study. WHO operational definitions of the preventive behaviors were adopted for this study. Observations were fixed at gates and or entrances of public service providing facilities including health facilities, workplaces, banks, food establishments, religious institutions, public transport waiting areas, street crossing sites and market places.

Results: A total 12056 individual observations with 52% males, and 82% in 18-50 years old age group were involved in this study. The results showed a sharp rise in the practice of respiratory hygiene (from 14% in week one to 77% in week 10), while those of hand hygiene (from 24.2% in week one to 23.7% in week 10) and physical distancing (from 34.4% in week one to 35.2% in week eight) changed little during the last weeks of observation. The three combined practices of hand and respiratory hygiene and physical distancing at individual level increased at a slow pace from 4.7% in week one to 8.1% in week 10.

Conclusion: As adherence by the public to the preventive measures seems to be higher among those with relatively better awareness and during times of government mandates, mass education. The preventive measures need to be comprehensive with strict enforcement, as situations require.

Key words: COVID-19, non-pharmaceutical intervention, community practice, COVID-19 prevention.

Introduction

The COVID-19 pandemic is assumed to originate from Wuhan, China on Dec 2019 (1). Since then, it has spread to Europe, USA, and lately to Africa. The nature of transmission of the infection is showed to be through droplets, aerosols, and contact transmission (2). The possibility of transmission through faeco-oral and airborne routes is still under investigation (2, 3). Pre-asymptomatic cases facilitate most of the transmission (4, 5). The mortality pattern is devastating among elder age groups because of limited defensive mechanisms (6, 7).

The application of the infection prevention and control (IPC) measures for emerging infectious diseases in health care settings can be managed with difficulty and confusion, especially in outbreaks where resources are limited (8). Yet such measures, including early identification, prompt isolation , proper patient placement, adequate space ventilation and proper use of personal protective equipment are reported to be important in the prevention and control for the transmission of COVID-19 (9). To reduce the burden on healthcare, community prevention activities are considered best strategies as they help slow transmission of the virus within communities (10). Community prevention strategies are a set of actions that persons and communities can implement to slow the transmission of COVID-19 by practicing hand hygiene, respiratory hygiene, and social distancing (11).

The Ministry of Health of Ethiopia is providing daily updates on the number of new infections and reported mortalities due to COVID-19. The first case in Ethiopia was declared on March 13, 2020, and the number of cases grew very slowly until the end of the first week of May 2020 (from 1 case on March 13 to 194 cases on 08 May 2020) (12). The number of reported cases continued to rapidly increase since then reaching 110,074 cases and 1,706 deaths on 01 December 2020 (the time of the write-up of this manuscript) (11).

In Ethiopia, the Ministry of Health (FMOH) and the Ethiopian Public Health Institute have created awareness about the virus transmission and prevention method to the public

using different approaches, including mass media. The interventions taken by the government and communities range from banning of public gatherings (of more than four persons) to limiting the number of passengers in public transport. All public service rendering institutions (such as food establishments, market places, banks, and health facilities) are involved in providing facilities for hand washing and in maintaining a physical distance of at least one meter between their clients. Security forces and public authorities have shown direct involvement in enforcing the maintenance of mask use and keeping physical distance in public places such as marketplaces, public transport stations and terminals. These interventions have proven the declining of covid-9 transmission in other countries (13). Data on monitoring the status of these interventions, however, is lacking in Ethiopia. Thus, the purpose of this monitoring is to generate evidence on a weekly basis about the COVID-19 prevention practices of the residents of Addis Ababa (focusing on hand hygiene, physical distancing, and respiratory hygiene). The findings are expected to inform policy in order to sustain the related behaviors in a general population.

Methods

Study Area, Setting, and Period

Addis Ababa is the capital city of Ethiopia and is headquarters for the African Union and the Economic Commission for Africa. The city has a population of over four million and is sub-divided into ten administrative sub-cities. One of the biggest markets in Africa ("*Merkato*") is in the center of the city, and there are about 131 Orthodox churches and several mosques. About 67% of the total vehicles (estimated to be about 800,000) are registered within Addis Ababa. There are several state and private banks–the Commercial Bank of Ethiopia (a state enterprise) alone having over 120 branches in Addis Ababa.

This study was started as part of the preventive behavior monitoring activity within the Emergency Operation Center (EOC) established at the College of Health Sciences, Addis Ababa University. It was aimed at monitoring the level of community practice in terms of the three non-pharmaceutical interventions (NPI) for COVID-19 prevention (hand hygiene, physical distance, and respiratory hygiene). The study has been conducted by observing the behavior of people in sites where they get various social and business-related services: religious places, health facilities, market places, banks, public transport, food and drink establishments, street crossing sites, and workplaces. These sites were selected based on level of crowding, and thus the level of risk for COVID-19 transmission. A repeated cross-sectional observation design was conducted on a weekly basis during the period of April 25–June 28, 2020.

Study Population

The source population for the study was all individuals visiting the selected sites. The study participants were individuals who are found at the selected sites during the time of data collection.

Monitoring Protocol

Prior to the data collection, a monitoring protocol has been prepared. The protocol describes the observation site selection, defines the observation facility, operationally defines the NPI practices, and the time as well as the procedure for the observation. Eight sites were selected purposively based on selection criteria that included the provision of service functionality, availability of service users, and appropriateness of the location for observation relative to increased population mobility.

Data Collection Tools

Two types of data collection tools were used for the study - location observation checklist and individual NPI practice observation form. The location observation checklist was prepared to collect information regarding Global Positioning System (GPS) of each service facility location, presence of any enforcement, posted media related to COVID-19, and availability of handwashing facilities. The NPI practice observation tool contained information regarding he observed individuals' sex, estimated age, NPI practice in three categories (proper, improper, and none). Each of the NPI practices were operationally defined within the monitoring protocol following the WHO recommendation (14).

Data Collection Procedure

Data was collected twice a week (one weekday and one weekend day) from each observation site. The duration of observation per each site was fixed for a minimum of 30 minutes. One data collector was assigned per each sub-city, and one supervisor appointed for two data collectors. The field data were collected using Open Data Kit tool (ODK) at the peak hour when the number of people is highest at each observation site stated in the protocol (Figure 1). The daily recorded data were uploaded daily to the central server for data management on a weekly basis.

Insert Figure 1

Figure 1: Observational data collection sites in Addis Ababa

Variables

Only key variables that suited the data collection technique were used. Outcome variables were those related to the practice of hand hygiene, physical distancing, and respiratory hygiene. Sex and age were considered for the demography of study subjects. Age was stratified into 3 categories (<18, 18-50, >50 years) that could easily be identified when observing. There were few contextual variables related to the observation of individuals for proper practicing per the operational definitions and checking hardware's such as hand washing facilities.

Data management and analysis

The ODK based data was stored on a server on a database that was created for this purpose. Data was downloaded in excel format that was transferred into SPSS V 23 for data merging, cleaning, and analysis. Data is presented using descriptive statistics, Tables and Charts.

Ethical Considerations

The study has got ethical approval from the Institutional Review Board of the College of Health Sciences at Addis Ababa University. The survey uses a random observation tool that does not override the rights of any individual or institution, and the observation is done purely anonymously.

Results

A total of 12,056 people were observed from April 20, 2020 to June28, 2020. More than half of these (59.2%) were observed in the weekdays (Table 1).

Table 1: Number of people observed (April 20– June 14, 2020, Addis Ababa

(Insert Table 1)

A total of 6,383 (52.9%) males and 5,673 (47.1%) females were observed in the study. Among the observed, majority 9,832 (81.6%) were in the estimated age group of 18-50 years (Table 2).

Table 2: Demographic profile of participants by sub-city (April 20– June 14, 2020, Addis Ababa)

Insert Table 2

Proper hand hygiene in the community slowly increased from 24.2% in week one to 32.5% in week three, and then slowly declined to 23.7% in week 10. Similarly, proper physical distancing in the community increased from 34.4% in week one to 42.6% in week four, and then slowly declined to 35.2% in week eight. Proper respiratory hygiene in the community continuously increased from 14% in week one to 77% in week 10. Drivers' respiratory hygiene continuously increased from 13.8% in week one to 84.6% in week 10, and from no respiratory hygiene practice among assistant drivers in week one to 70.5% in week six then slowly declined to 54.1% in week 10 (Table 3). The results also showed that adherence to the government mandated less than or equal to 50% occupancy in commercial vehicles declined from 88.8% in week one to 68.8% in week 10.

Table 3: Weekly pattern of community practice of non-pharmaceutical interventions (Addis Ababa, 2020)

Insert Table 3

The results also indicated sharp rise in the practice of respiratory hygiene, while those of hand hygiene and physical distancing did not change or declined during the last week of observation (Figure 2).

Insert Figure 2

Figure 2: The overall trend of the proper community practices for the three universal precautions

There was a similar trend of increasing respiratory hygiene when this practice was disaggregated by facility type. However, the practice of respiratory hygiene showed a fast increase at all service provision sites observed, and the highest change was at banks (from 30% in week one to 90% in week 10) (Figure 3).

Insert Figure 3

Figure 3: The trend of the proper respiratory hygiene practices at different institution sites

The three simultaneous practices of hand and respiratory hygiene and physical distancing increased at a slow pace from 4.7% in week one to 10.8% in week four and then declined to 8.1% in week 10 (Figure not shown). Two combined practices of proper distancing and respiratory hygiene showed continuous improvement from 11.4% in week one to 32.2% in week nine. Similarly, two combined practices of proper hand and respiratory hygiene increased continuously from 6.8% in week one to 20.2% in week nine, while two combined practices of hand hygiene and physical distancing barely showed any change between week one (10.7%) and week 10 (11.5%) (Table 4).

Table 4: Combined differents community practices

Insert Table 4

Discussion

As SARS-CoV-2 (COVID-19) in the presence of limited vaccine or effective treatment, non-pharmaceutical interventions are the major strategic efforts to prevent the introduction of new infections. This study was based on community observation data on non-pharmaceutical interventions recommended by World Health Organization (WHO) (14). Overall, our results showed the use of mask was satisfactory, while hand hygiene and physical distancing were not to the optimum level.

Non-pharmaceutical interventions have been implemented to reduce transmission of COVID-19 in many countries. An earlier study in China has shown the non-pharmaceutical interventions to be effective in containing the outbreak, and that the number of cases were predicted to have been 67-fold higher at the time without these interventions (15) . However, adherence to these preventive practices has been seen to be declining in many countries in recent times. For instance, the Nigerian Medical Association has been reported to disregard the non-pharmaceutical prevention protocols by citizens (16).

The present study has shown major improvement in the practice of respiratory hygiene compared to hand hygiene and physical distance during the period of observation. The improvement showed significant change since week four (Week of May 4/2021) that matched with the time when security forces enforced the use of mask as mandated by the state of emergency to respond to the pandemic crisis. Although the State emergency was declared on 20 April 2020, its impact came later, just after a few weeks. This time has been used for preparations for local production of mask and availing hand hygiene facilities by public service rendering institutions. The State Emergency in Ethiopia at the time of the study was characterized to have partial lockdown of public places, including closing of educational facilities and workplaces. According to this State of Emergency, public mobility was not restricted for seeking food, transport, health care, and marketing services. There are reports from other studies that intensive interventions with lockdown periods would need to be in place for the preventive measures to be effective since adherence remains to interrupt covid-19 transmission (17).

State Emergency had a provision that mandated private and government service providers to provide hand washing facilities with detergents for their clients. The practice of proper hand hygiene showed little change over the 10 weeks of observation, and could not go beyond 30%. Two reasons were showed from the qualitative observation. First, the media company and involvement of the enforcing volunteers had massive interventions in the first month during the data collection period, especially around churches, transport stations, and other public places. This has contributed a change in hand washing practice in that month, but not much beyond that month. On the other hand, the unavailability of water and soap for washing hand could not be sustained in many facilities, except few public services providing institution such as banks. Our qualitative observation has documented this practice. The provision of soap was challenged first that was followed by the shortage and total disappearance of water in many fronts of public service providers. We believe these happened at the time of weak enforcement when security and other partners lifted off their started effort. There was a shift from washing hand to using hand sanitizers by some members of the population, especially the youth, and our result did not include that thus may underestimate the proportion of handwashing in a community. The youth usually did not use to visit the facilities we have opted, thus unlikely to be covered by our study.

The maintenance of physical distancing at a minimum of 1m between any two individuals had a similar trend with hand washing practice, although the former had an overall performance of below 40% relative to the 30% of the later practice. Physical distancing was a lot challenged in many spheres of social services, predominantly observed in open markets, at times of walking on street, when crossing streets, and seeking public transport sectors. The context of public services is likely to affect the performance of physical distancing. Individuals in an Ethiopian open market traditionally cluster around a seller in order to make transactions. Limited and unpredicted public transport services at peak hours make service seekers get closer around the gate of transport vehicles. There is a public health concern that such a low level of physical distancing may enhance transmission. The duration and distance of the exposure to SARS infection have been shown an important predictor of the transmission (18). A previous systematic review of

172 studies reported that physical distancing of one meter was associated with a large reduction in the number of new infections in different countries (19). Taking the economic status of Ethiopia, lack of change in the physical distancing at the public level in this study was a missed opportunity to avert the occurrence of new cases in the city given. Hand hygiene and physical distancing are an effective low-cost intervention that could reducing the number of new cases, hence the severity of cases through reduce viral inoculation (20).

The problem of not practicing physical distancing was observed during the Ethiopian religious festivals. This is common in our traditional open market settings. Inconsistent to this finding, a study from China reported that almost all respondents avoided going to public places and reduced celebrating spring festivals due to COVID-19 epidemic in the country (21). The reasons for the difference might be associated with the socio-cultural contexts of the study population in a given country and the degree of enforcing preventive practices.

The extent and trend of mask utilization had visible difference compared to physical distancing and other practices. Mask utilization has shown an increasing trend until it stabilizes at about 80% towards the end of our study time. Many factors operated. The continued security enforcement, mass media mobilization, the banning of visitors and passengers to get a service without a mask, and the provisions of regular information about covid-19 using national TVs and radios were the main ones. The multiple sources of information and enforcement is believed to enhance the attitude of service seekers towards the use of mask which greatly modified their behavior towards the use of mask. We cannot deny at all that the access to multiple types of mask, such as surgical, N95, and cloth, their local production at affordable prices has greatly changed the trend of mask utilization. The local production of face masks in low-middle income countries has been indicated as a strategy to contain the epidemic (22). Systematic reviews have shown the frequent practice of hand hygiene, the use of physical distancing of 1m or more, and mask utilization have a potential benefit of lowering the covid-19 transmission (19, 23). The time of the observation, before or after the State emergency, and the age of study subjects could make a difference in the proportion of mask using. There is a preference

of using mask among young population in Poland, making the figure at 60%, a study undertaken before the government's enforcement (24), which is relatively lower than ours. The study in Poland was among young population, which is not consistent with ours. Our study was undertaken after the State Emergency, which has brought multiple interventions to impact the behaviors of individuals in using mask. Overall, the universal use of mask in a public has been shown as an effective tool to reduce the transmission of covid-19 in previous studies targeting (25, 26).

We disaggregated the mask using by type of facilities in order to get insights about the factors affecting mask using. The trend of respiratory hygiene practice increased in the entrances to the health care facilities and banks and transport services compared with other facilities included in this study. There were assigned controllers checking for the use of face-mask who did not allow individuals to enter without using it. The periodic control by the security forces on the streets and around churches and by the guards of supermarkets was also observed while no control was there in the studied work places, open markets, and around food and drink establishments. The problem of respiratory hygiene in the food and drink establishments requires close attention because people used to eat in groups and physical distancing was almost not practically applied. Onsite dinning with indoor and outdoor seats was identified as a risky situation by CDC (10).

The relatively improved practices at bank sites may be related to the fact that these institutions have committed the resources to provide hand washing facilities with detergents and that their clients would be relatively higher level of awareness and favorable attitude towards the preventive practices. In addition, the presence of security guards at the entrance/ gate of a bank has a lot enhanced compliance. As there are variabilities in people's beliefs about the effectiveness as well as the rates of engagement in the use of non-pharmaceutical interventions, public health efforts should focus on increasing perceived severity and threats of the COVID-19, while promoting the preventive measures as effective tools (27, 28) and enforcing the use of them as necessary.

The comprehensive use of the three practice at each individual level was surprisingly very low, just below 10%. Any two combination was also low, that did not have a public health relevance. The risk characterization by the level of combination is a concern given the presence of the covid-19 spread in a population. The very limited practice in a combined practice is an indication of a challenge and a threat to sustain the spread of transmission. WHO greatly advocates the complete and regular use of these practices at every individual level when getting outside home (29). With such low level of practice coupled with limited distance of exposure, there could be an increased likely of covid-19 transmission in a population any time, hence exploding the epidemic. The trend of viral related pneumonia had demonstrated a declining trend that is asserted with the likely provisions of wearing mask, hand hygiene and social distancing in a Taiwan epidemiological study (30). WHO informs that the only use of masks in the context of COVID-19 suggested the risk of false sense of security, which could lead to potential less compliance of other behaviors because of the risk of self-contamination during the manipulation of the mask.

This study has strengths in view of observing individuals randomly in major public rendering services. The provision of regular information to the Ministry of Health was an important input to enhance informed decisions to the public. However, observing individuals throughout the day in each facility was not possible because of limited resources. We assumed observing individuals at the time of transmission in the presence of increased mobility and crowding provides better insights of the transmission potential. Another area of limitation is in ascertaining the appropriate use of masks regarding checking fitness. The context of this study could not allow to optimize complete information in the general population. The benefit of this type of observation was to inform policy using research evidences.

Conclusions and Recommendations

The overall community prevention practices of COVID-19 pandemic were very low in Addis Ababa, Ethiopia. Despite the relatively low adherence of the community on proper hand washing and proper physical distancing, the success of proper respiratory hygiene was encouraging which requires inclusively the proper practice of hand hygiene and

physical distancing. We call to strengthen social mobilization using available medias and consistent enforcement mechanisms.

Funding: The data collection was funded by the School of Public Health of Addis Ababa University. The Authors have contributed professionally in view of supporting the Ministry of Health in enhancing proactive informed decisions.

Acknowledgements: The authors are grateful to the College of Health Sciences and the School of Public Health of Addis Ababa University for allowing this study to happen. Dawit Wendimagegn, the Executive director of the CHS, is greatly thanked for his continued encouragement to complete this study.

Competing interest: I do not have any financial competing interests and any conflicts in this publication with any other person or institution.

Author’s Contributions

DH tabled a research idea on the need of generating the status of community practice. AK was involved in the writing of the proposal and drafting the manuscript. AK and SW were involved in the analysis of data. MT was involved in handling the field qualitative information. WA was involved in the design of data template in ODK. YT and TA were involved in the design of observation tools. All were involved in editing the proposal and manuscript.

References

1. Chen X, Yu B. First two months of the 2019 Coronavirus Disease (COVID-19) epidemic in China: real-time surveillance and evaluation with a second derivative model. . Glob health res policy. 2020;5(7(2020). https://doi.org/10.1186/s41256-020-00137-4).
2. Beattie RM, Ashton JJ, Penman ID. COVID-19 and the gastrointestinal tract: emerging clinical data. Frontline Gastroenterology. 2020:flgastro-2020-101507.
3. Liu J, Liao X, Qia nS, Yuan J, Wang F, Liu Y, et al. Community transmission of severe acute respiratory syndrome coronavirus 2, Shenzhen, China, 2020. Emerg Infect Dis. 2020;26(6).
4. Guoqing Q, Naibin Y, Ada HYM, Liping W, Guoxiang L, Xueqin C, et al. COVID-19 Transmission Within a Family Cluster by Presymptomatic Carriers in China Clinical Infectious Diseases. 2020;71(15):861-.
5. Yu X, Yang R. COVID-19 transmission through asymptomatic carriers is a challenge to containment. Influenza Other Respir Viruses. 2020;14(4):474–5.
6. Dudley JP, Lee NT. Disparities in Age-specific Morbidity and Mortality From SARS-CoV-2 in China and the Republic of Korea. Clin Infect Dis. 2020;71(15):863-5. Epub 2020/04/02.
7. Palaiodimos L, Kokkinidis DG, Li W, Karamanis D, Ognibene J, Arora S, et al. Severe obesity, increasing age and male sex are independently associated with worse in-hospital outcomes, and higher in-hospital mortality, in a cohort of patients with COVID-19 in the Bronx, New York. Metabolism. 2020;108:154262. Epub 2020/05/19.
8. World Health Organization. Infection prevention and control of epidemic- and pandemic-prone acute respiratory infections in health care 2014 [cited 2020 April 2]; Available from: https://apps.who.int/iris/bitstream/handle/10665/112656/9789241507134_eng.pdf?sequence=1.
9. European Center for Diseases Prevention and Control. Infection prevention and control and preparedness for COVID-19 in healthcare settings. [cited 2020 April]; Available from: https://www.ecdc.europa.eu/sites/default/files/documents/Infection-prevention-control-for-the-care-of-patients-with-2019-nCoV-healthcare-settings_update-31-March-2020.pdf.
10. Centers for Diseases Control and Prevention. Implementation of Mitigation Strategies for Communities with Local COVID-19 Transmission. [cited 2020 April]; Available from: https://www.cdc.gov/coronavirus/2019-ncov/downloads/community-mitigation-strategy.pdf.
11. World Health Organization. Corona virus disease (COVID-19) advice for the public.https://www.who.int/emergencies/diseases/novel-coronavirus-2019/advice-for-public. [cited 2020 April]; Available from: https://www.who.int/emergencies/diseases/novel-coronavirus-2019/advice-for-public.
12. Worldometers. Ethiopia: Corona Virus Cases. https://www.worldometers.info/coronavirus/country/ethiopia/. 2020.
13. Pei S, Kandula S, Shaman J. Differential Effects of Intervention Timing on COVID-19 Spread in the United States. medRxiv. 2020:2020.05.15.20103655.
14. World Health Organization. Coronovirus. https://www.who.int/health-topics/coronavirus#tab=tab_2. 2020.

15. Shengjie L, Nick WR, Liangcai Z, Olivia Prosper, Wei L, Jessica RF, Amy W, et al. Effect of non-pharmaceutical interventions to contain COVID-19 in China. Nature. 2020;585:410-3.

16. Alexander D. NMA Condemns Non-Adherence To COVID-19 Prevention Protocols. 2020 [cited 2021 January]; Available from: https://consumerconnectng.com/8186.

17. Yacong B, Cui G, Changqing L, Samuel YSW, Alexis KHL, Xiang QL. Effectiveness of non-pharmaceutical interventions on COVID-19 transmission in 190 countries from 23 January to 13 April 2020. International Journal of Infectious diseases. 2021;102:247-51. ttps://doi.org/10.1016/j.ijid.2020.10.066.

18. Rea E, Laflèche J, Stalker S, uarda BK, Shapiro H, Johnson I, et al. Duration and distance of exposure are important predictors of transmission among community contacts of Ontario SARS cases. https://doi.org/10.1017/S0950268806007771. Epidemiology and infection. 2007;135(6):914–21.

19. Chu DK, Akl EA, Duda S, Solo K, Yaacoub S, Schünemann HJ. Physical distancing, face masks, and eye protection to prevent person-to-person transmission of SARS-CoV-2 and COVID-19: a systematic review and meta-analysis. Lancet. 2020;395(10242):1973-87.

20. Dalton CB, Corbett SJ, Katelaris AL. COVID-19: implementing sustainable low cost physical distancing and enhanced hygiene. Medical journal of Australia. 2020;212(10):443–6.e1. https://doi.org/10.5694/mja2.50602.

21. Global Times. Beijing districts, companies to enter into a state of emergency: official. 2020 [cited 2020 March]; Available from: https://www.globaltimes.cn/content/1211098.shtml.

22. Missoni E, Armocida B, Formenti B. Face Masks for All and All for Face Masks in the COVID-19 Pandemic: Community Level Production to Face the Global Shortage and Shorten the Epidemic. Disaster medicine and public health preparedness. 2020:1-5. https://doi.org/10.1017/dmp.2020.207.

23. Jefferson T, Del Mar CB, Dooley L, Ferroni E, Al-Ansary LA, Bawazeer GA, et al. Physical interventions to interrupt or reduce the spread of respiratory viruses. The Cochrane database of systematic reviews, 2011(7), CD006207. . Cochrane Database Syst Rev. 2011;7(CD006207). https://doi.org/10.1002/14651858.CD006207).

24. Matusiak Ł, Szepietowska M, Krajewski PK, Białynicki-Birula R, zepietowski JC. The use of face masks during the COVID-19 pandemic in Poland: A survey study of 2315 young adults. Dermatologic therapy. 2020(e13909):Advanced online publication. https://doi.org/10.1111/dth.13909.

25. Jefferson T, Del Mar C, Dooley L, Ferroni E, Al-Ansary LA, Bahamdan SA, et al. Physical interventions to interrupt or reduce the spread of respiratory viruses: systematic review. Br Med J. 2009;339(b3675).

26. Zhang L, Peng Z, Ou J, Zeng G, Fontaine RE, Liu M, et al. Protection by face masks against influenza A(H1N1)pdm09 virus on trans-Pacific passenger aircraft, 2009. Emerg Infect Dis. 2013;19(9). Epub 2013/08/24.

27. Van den Broucke S. Why health promotion matters to the COVID-19 pandemic, and vice versa. Health Promot International. 2020;35(2):181-6. doi: 10.1093/heapro/daaa042.

28. Shahnazi H, Ahmadi-Livani M, Pahlavanzadeh B, Rajabi A, Shoaib MH, Charkazi A. Shahnazi, H., Ahmadi-Livani, M., Pahlavanzadeh, B. et al. Assessing preventive health behaviors from COVID-19: a cross sectional study with health belief model in Golestan Province, Northern of Iran. Infectious Disease Poverty. 2020;0(157):https://doi.org/10.1186/s40249-020-00776-2.

29. World Health Organization. Coronavirus disease (COVID-19) advice for the public. 2021 [cited 2021 January]; Available from: https://www.who.int/emergencies/diseases/novel-coronavirus-2019/advice-for-public.

30. Chiu N, Chi H, Tai Y, Peng C, Tseng C, Chen C, et al. Impact of Wearing Masks, Hand Hygiene, and Social Distancing on Influenza, Enterovirus, and All-Cause Pneumonia During the Coronavirus Pandemic: Retrospective National Epidemiological Surveillance Study. J Med Internet Res. 2020;8:e21257. doi: 10.2196/.

Figures and Tables are in one file Click here to access/download;Figure;Figures and tables Plos 1.docx

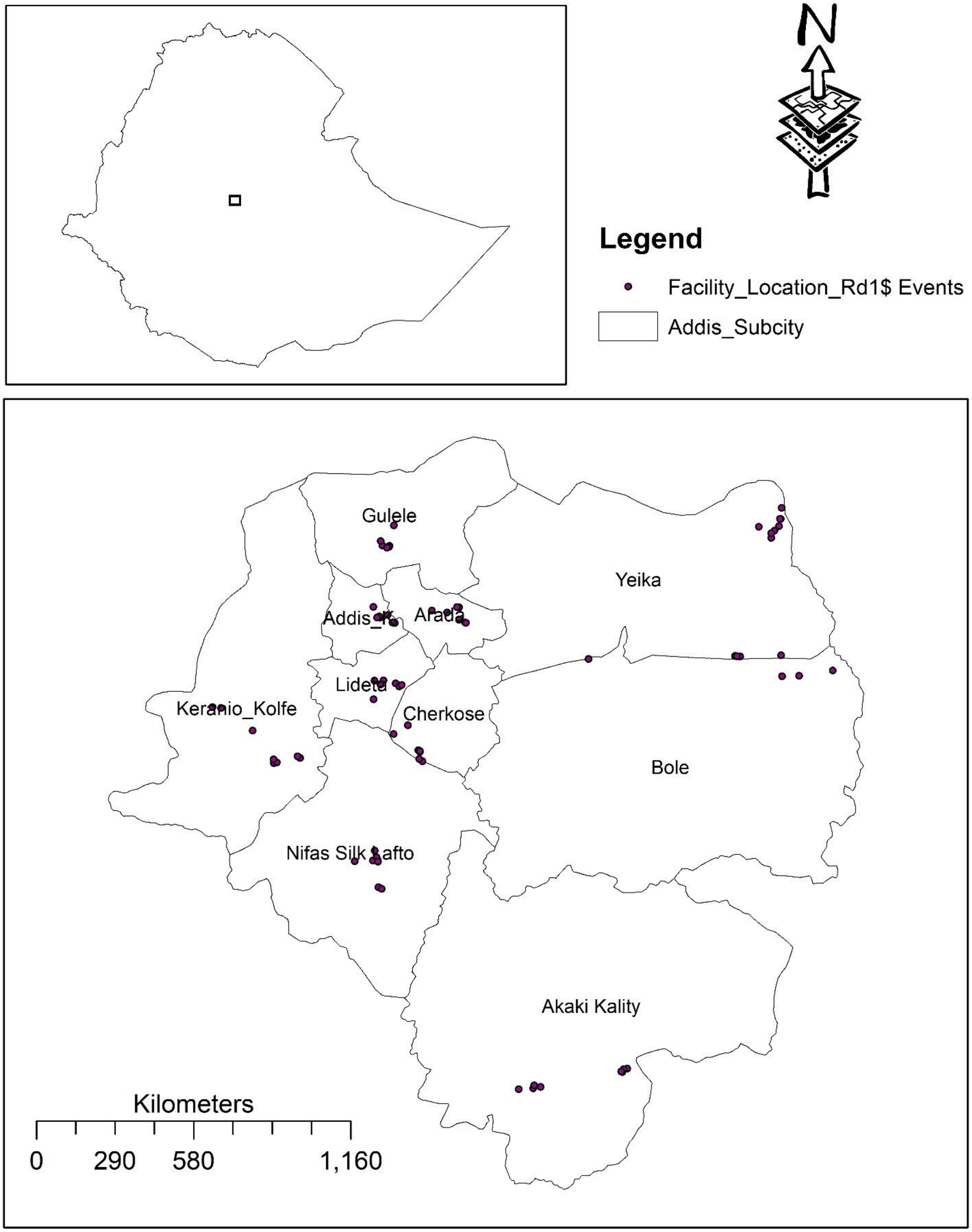

Figure 1: Observational data collection sites in Addis Ababa

Table 1: Number of people observed (April 20– June 14, 2020, Addis Ababa)

Insert Table 1

Observation weeks	Weekdays observations # (%)	Weekend observations # (%)	Total observations # (%)
Week 1 (Apr20-26)	225 (26.3)	629 (73.7)	854 (7.08)
Week 2 (Apr27-May3)	723 (62.1)	442 (37.9)	1165 (9.66)
Week 3 (May 4-May 10)	804 (63.6)	460 (36.4)	1264 (10.48)
Week 4 (May 11-May 17)	719 (60.0)	480 (40.0)	1199 (9.95)
Week 5 (May 20- May 24)	775 (62.2)	470 (37.8)	1245 (10.33)
Week 6 (May 27-May 31)	706 (59.1)	488 (40.9)	1194 (9.90)
Week 7 (June 3-June 7)	799(62.0)	489 (38.0)	1288 (10.68)
Week 8 (June 10-June 14)	788(61.7)	490 (38.3)	1278 (10.60)
Week 9 (June 15- June 21)	802 (62.6)	479 (37.4)	1281 (10.63)
Week 10 (June 22- June 28)	798 (62.0)	490 (38.0)	1288 (10.68)
**Total**	**7139 (59.2)**	**4917 (40.8)**	**12056 (100)**

Table 2: Demographic profile of participants by sub-city (April 20– June 14, 2020, Addis Ababa)

Variable	Total n (%) (n=12056)
Sex	
Male	6383 (52.9)
Female	5673 (47.1)
Age*	
<18 years	551 (4.6)
18-50 years	9832 (81.6)
>50 years	1673 (13.9)

*Age categories were made broad purposely in order to identify the closest age using observation without asking an individual

Table 3: Weekly pattern of community practice of non-pharmaceutical interventions (Addis Ababa, 2020)

NPI	NPI Practices	Week 1 (Apr20-26)	Week 2 (Apr27-May3)	Week 3 (May 4-10)	Week 4 (May 11- 17)	Week 5 (May20- 24)	Week 6 (May 27-31)	Week 7 (June 3-7)	Week 8 (June10-14)	Week 9 (June15-21)	Week 10 June 22-28
Hand hygiene	**Proper hand hygiene**	**178 (24.2)**	**328 (33.1)**	**350 (32.5)**	**323 (32.0)**	**305 (28.8)**	**26 1 (25.9)**	**288 (26.1)**	**290 (26.8)**	**281 (28.5)**	**254 (23.7)**
	Improper hand hygiene	88 (11.9)	93 (9.4)	101 (9.4)	76 (7.5)	78 (7.4)	80 (7.9)	121 (10.9)	105 (9.7)	123 (12.4)	103 (9.6)
	No hand hygiene	469 (63.8)	571 (57.6)	625 (58.1)	610 (60.4)	674 (63.8	667 (66.2)	692 (62.8)	684 (63.4)	582 (59.1)	715 (66.7)
	**Total**	**735**	**992**	**1076**	**1009**	**1057**	**1008**	**1101**	**1079**	**986**	**1072**
Physical distance	**Proper physical distance**	**294 (34.4)**	**462 (39.7)**	**491 (38.8)**	**511 (42.6)**	**46 7 (37.5)**	**444 (37.2)**	**479 (37.2)**	**496 (38.8)**	**503 (39.3)**	**454 (35.2)**
	Improper physical distance	408 (47.8)	531 (45.6)	593 (46.9)	551 (46.0)	655 (52.6)	643 (53.9)	699 (54.3)	662 (51.8)	711 (55.5)	710(55.1)
	No physical distance	152 (17.8)	172 (14.8)	180 (14.2)	137 (11.4)	123 (9.9)	107 (9.0)	110 (8.5)	120 (9.4)	67 (5.2)	124(9.6)
	**Total**	**854**	**1165**	**1264**	**1199**	**1245**	**1194**	**1288**	**1278**	**1281**	**1288**
Respiratory hygiene	**Proper respiratory hygiene**	**205 (24.0)**	**301 (25.8)**	**405 (32.0)**	**575 (48.0)**	**746 (59.9)**	**845 (70.8)**	**985 (76.5)**	**980 (76.7)**	**999 (78.0)**	**995 (77.3)**
	Improper respiratory hygiene	79 (9.3)	174 (14.9)	213 (16.9)	271 (22.6)	151 (12.1)	153 (12.8)	198 (15.4)	214 (16.7)	208 (16.2)	201 (15.6)
	No respiratory hygiene	570 (66.7)	690 (59.2)	646 (51.1)	353 (29.4)	348 (28.0)	196 (16.4)	105 (8.2)	84 (6.6)	74 (5.8)	92 (7.1)
	**Total**	**854**	**1165**	**1264**	**1199**	**1245**	**1194**	**1288**	**1278**	**1281**	**1288**
Drivers' respiratory hygiene	**Proper respiratory hygiene**	**11 (13.8)**	**34 (23.0)**	**91 (55.2)**	**133 (78.7)**	**159 (84.6)**	**170 (89.5)**	**167(84.8)**	**165(82.9)**	**156(82.5)**	**168 (84.6)**
	Improper respiratory hygiene	1 (1.3)	11 (7.4)	15 (9.1)	11 (6.5)	23 (12.2)	17 (8.9)	29 (14.7)	30 (15.1)	30 (15.9)	27 (13.6)
	No respiratory hygiene	68 (85.0)	103 (69.6)	59 (35.8)	26 (15.3)	6 (3.2)	3 (1.6)	1 (0.5)	4 (2.0)	3 (1.6)	4 (2.0)
	**Total**	**80**	**148**	**165**	**170**	**188**	**190**	**197**	**199**	**189**	**199**
Driver assistants' respiratory hygiene	**Proper respiratory hygiene**	**0%**	**29 (19.6)**	**60 (37.3)**	**87 (52.1)**	**119 (62.6)**	**141 (70.5)**	**135 (69.9)**	**118 (60.5)**	**111 (59.0)**	**106 (54.1)**
	Improper respiratory hygiene	3(4.0)	19(12.8)	29 (18.0)	44 (26.3)	61 (32.1)	48 (24.0)	57 (29.5)	72 (36.9)	73 (38.8)	80 (40.8)
	No respiratory hygiene	72 (96.0)	100 (67.6)	72 (44.7)	36 (21.6)	10 (5.3)	11 (5.5)	1 (0.5)	5 (2.6)	4 (2.1)	10 (5.1)
	**Total**	**75**	**148**	**161**	**167**	**190**	**200**	**193**	**195**	**188**	**196**
Vehicle occupant capacity	**Less than or equal to 50% capacity**	**71 (88.8)**	**122 (83.0)**	**114 (69.1)**	**110 (65.1)**	**128 (68.1)**	**128 (67.4)**	**136(69.0)**	**140(70.4)**	**132(69.8)**	**137(68.8)**
	Greater than 50% capacity	9 (11.3)	25 (17.0)	51 (30.9)	59 (34.9)	60 (31.9)	62 (32.6)	61 (31.0)	59 (29.6)	57 (30.2)	62 (31.2)
	**Total**	**80**	**147**	**165**	**169**	**188**	**190**	**197**	**199**	**189**	**199**

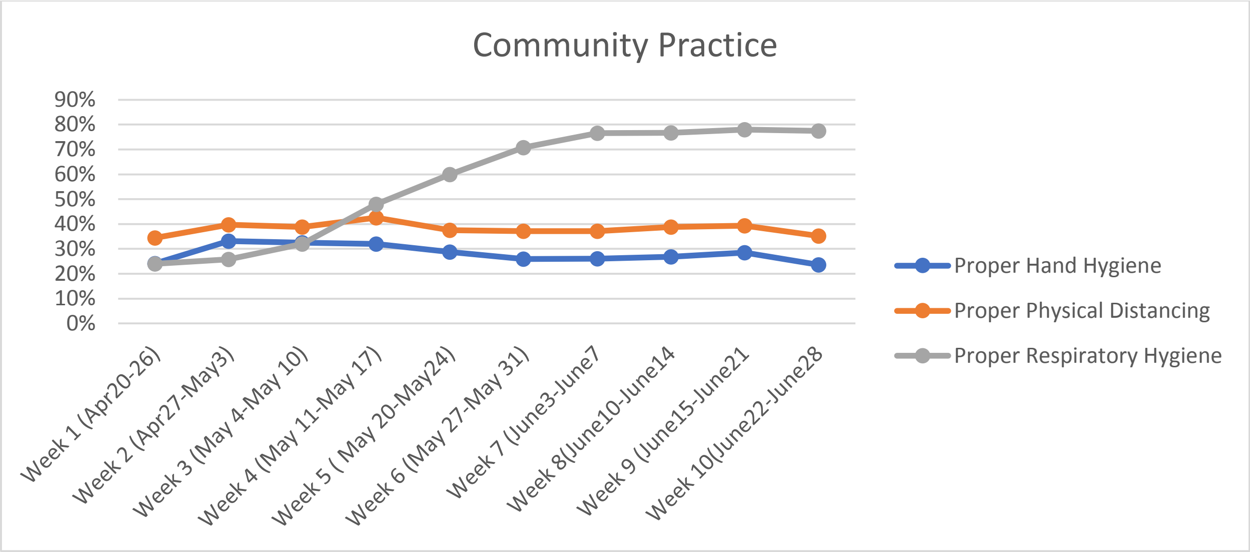

Figure 2: The overall trend of the proper community practices for the three universal precautions

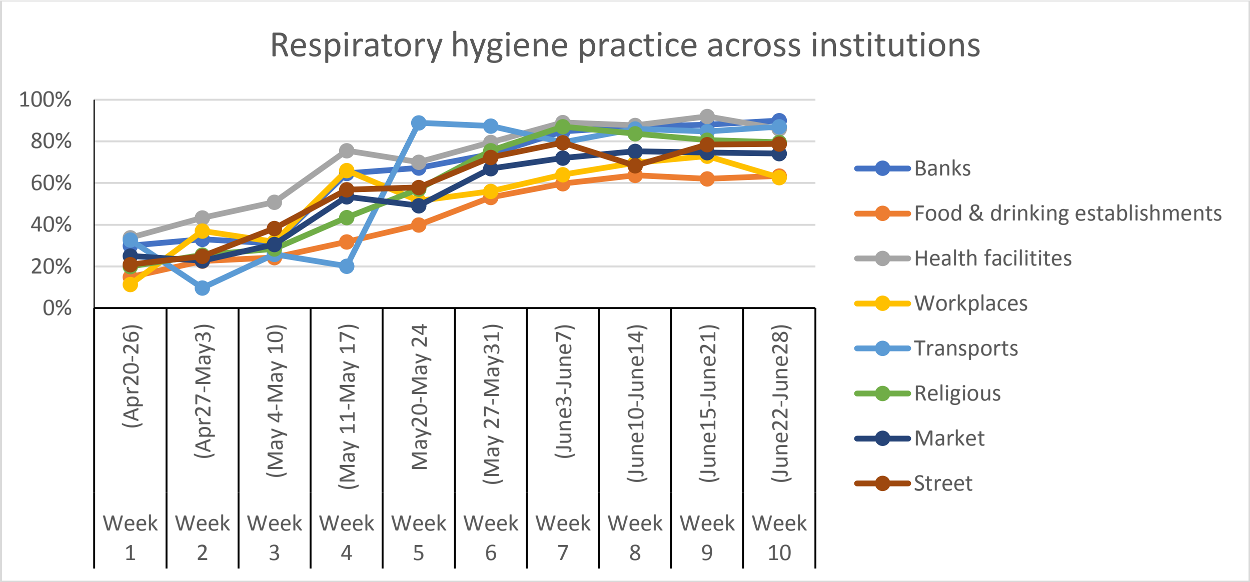

Figure 3: The trend of the proper respiratory hygiene practices at different institution sites

Table 4: Combined differents community practices

Risk category	Risk classification	Week 1 (Apr 20-26)	Week 2 (Apr 27-May 3)	Week 3 (May 4-10)	Week 4 (May 11-17)	Week 5 May 20-24	Week 6 May 27-31	Week 7 June 3-7	Week 8 June10-14	Week 9 June15-21	Week 10 June22-28	Total
**Proper hand hygiene & distancing & respiratory**	Low risk	40 (4.7)	79(6.8)	96 (7.6)	130 (10.8)	99 (8.0)	95 (8.0)	98 (7.6)	123 (9.6)	113 (8.9)	100 (7.8)	973 (8.1)
**Proper distance & respiratory**	Low risk	97 (11.4)	150(12.9)	206 (16.3)	264 (22.0)	311 (25.0)	334 (28.0)	396 (30.7)	405 (31.7)	413 (32.2)	383 (29.7)	2959 (24.5)
**Proper hand hygiene & distancing**	Medium risk	91 (10.7)	172(14.8)	174 (13.8)	180 (15.0)	149 (12.0)	118 (9.9)	116 (9.0)	140 (11.0)	130 (10.3)	110 (8.6)	1380 (11.5)
**Proper hand and resp hygiene**	Medium risk	58 (6.8)	127(10.9)	163 (12.9)	205 (17.1)	183 (14.7)	193 (16.2)	237 (18.4)	250 (19.6)	240 (20.2)	227 (17.8)	1883 (15.8)
**Only mask**	High risk	73 (8.5)	68(5.8)	90 (7.1)	165 (13.8)	276 (22.2)	328 (27.5)	348 (27.0)	375 (29.3)	290 (24.1)	381 (29.8)	2394 (20.0)
**Only physical distancing**	High risk	130 (15.2)	183(15.7)	181 (14.3)	176 (14.7)	87 (7.0)	68 (5.7)	50 (3.9)	54 (4.2)	59 (4.6)	47 (3.7)	1035 (8.6)
**Only hand hygiene**	High risk	69 (8.1)	108(9.3)	109 (8.6)	68(5.7)	72(5.8)	45(3.8)	33(2.6)	23(1.8)	24(1.9)	17(1.3)	568 (4.7)
**No hand & respiratory hygiene & no distancing**	High risk	305 (35.7)	350 (30.0)	375 (29.7)	248 (20.7)	211 (16.9)	161 (13.5)	162 (12.6)	141 (11.0)	124 (9.8)	169 (13.1)	2246 (18.7)

*Risk classification was made based on the level of three practices at individual level. It is a relative term: an individual practicing the three is in low risk, while none of only doing hand hygiene and physical distance in high-risk group. An individual with two combination is in the medium risk.

Data in Spss

Click here to access/download

Supporting Information

Ten_Weeks combined_26_7_12 USE.sav
