# Supplementary material for: Trends in non-pharmaceutical intervention (NPI) related community practice for the prevention of COVID-19 in Addis Ababa, Ethiopia
Source: PLoS One. 2021 Nov 23;16(11):e0259229. doi: 10.1371/journal.pone.0259229 (PMC8610281; doi:10.1371/journal.pone.0259229)
Supplement: Attachment — Submitted filename: PONE-D-21-03031_R1 Edits by Academic Editor.pdf [file pone.0259229.s007.pdf]

PLOS ONE

Trends in non-pharmaceutical intervention (NPI) related community practice for the prevention of COVID-19 in Addis Ababa, Ethiopia

--Manuscript Draft--

Manuscript Number:	PONE-D-21-03031R1
Article Type:	Research Article
Full Title:	Trends in non-pharmaceutical intervention (NPI) related community practice for the prevention of COVID-19 in Addis Ababa, Ethiopia
Short Title:	Community practice for the prevention of Covid-19
Corresponding Author:	Yifoker Tefera<br>Addis Ababa University<br>aDDIS ABABA, ETHIOPIA ETHIOPIA
Keywords:	COVID-19; non-pharmaceutical intervention; community practice; COVID-19 prevention
Abstract:	Background: The COVID-19 pandemic had affected Ethiopia since March 13, 2020, when the first case was detected in Addis Ababa. Since then, the incidence of cases has continued to increase day by day. As a result, the health sector has recommended universal preventive measures to be practiced by the public. However, studies on adherence to these preventive measures are limited to inform evidence-based policy-making interventions.<br><br>Objective: To monitor the status of preventive practices of the population related to hand washing, physical distancing, and respiratory hygiene practices at selected sites within the city of Addis Ababa.<br>Methods: A weekly cross-sectional non-participatory observation was done during the period of April-June, 2020. Data was collected using Open Data Kit (ODK) tool in ten public sites involving eight public facilities targeted for individual observations. Ten individuals were randomly observed at each facility over two days a week at peak hours of public services WHO operational definitions of the preventive behaviors were adopted for this study. Observations were anonymously fixed at gates or entrances of public facilities and places.<br>Results : 12,056 individual observations with 53% males and 82% in 18-50 years age group were involved in this study. There was an increase in the practice of respiratory hygiene (from 14% in week one to 77% in week 10), while those of hand hygiene and physical distancing changed little over the weeks from their baseline of 24% and 34%, respectively. Overall, respiratory hygiene has shown an increased rate of 6% per week, while hand hygiene and physical distancing had <1%. Females and age group 18-50 years had increased practice in respiratory hygiene with no difference in the two practices. Respiratory hygiene took about six weeks to reach a level of 77% from its baseline of 24%, making an increment of about 9% per week.<br>Conclusion: The public practice of respiratory hygiene was relatively better than hand hygiene and physical distancing. It has shown visible weekly changes towards an increasing trend. Regularly sustained public mobilization and mass education are required to sustain the achievements gained in respiratory hygiene and improving hand hygiene and physical distancing.
Order of Authors:	Abera Kumie
	Damen Hailemariam
	Samson Wakuma
	Yifoker Tefera
	Teferi Abegaz
	Worku Tefera
	Mulugeta Tamire
	Wondimu Ayele

Powered by Editorial Manager® and ProduXion Manager® from Aries Systems Corporation

	Shibabaw Yirsaw
**Opposed Reviewers:**	
**Response to Reviewers:**	Dear Reviewers<br>The reviewing questions and concerns were very helpful. We have all accommodated giving a point by point response to each concern in three separate files. The English language was heavily edited.<br>We are hoping to satisfy your concern.<br><Many thanks for the understanding
**Additional Information:**	
**Question**	**Response**
**Financial Disclosure**<br><br>Enter a financial disclosure statement that describes the sources of funding for the work included in this submission. Review the submission guidelines for detailed requirements. View published research articles from *PLOS ONE* for specific examples.<br><br>This statement is required for submission and **will appear in the published article** if the submission is accepted. Please make sure it is accurate.<br><br>**Unfunded studies**<br>Enter: *The author(s) received no specific funding for this work.*<br><br>**Funded studies**<br>Enter a statement with the following details:<br>• Initials of the authors who received each award<br>• Grant numbers awarded to each author<br>• The full name of each funder<br>• URL of each funder website<br>• Did the sponsors or funders play any role in the study design, data collection and analysis, decision to publish, or preparation of the manuscript?<br>• **NO** - Include this sentence at the end of your statement: *The funders had no role in study design, data collection and analysis, decision to publish, or preparation of the manuscript.*<br>• **YES** - Specify the role(s) played.<br><br>* typeset	The author(s) received no specific funding for this work.
**Competing Interests**<br><br>Use the instructions below to enter a	The authors have declared that no competing interests exist.

Powered by Editorial Manager® and ProduXion Manager® from Aries Systems Corporation

competing interest statement for this submission. On behalf of all authors, disclose any competing interests that could be perceived to bias this work—acknowledging all financial support and any other relevant financial or non-financial competing interests.<br><br>This statement is required for submission and **will appear in the published article** if the submission is accepted. Please make sure it is accurate and that any funding sources listed in your Funding Information later in the submission form are also declared in your Financial Disclosure statement.<br><br>View published research articles from *PLOS ONE* for specific examples.<br><br>**NO authors have competing interests**<br><br>Enter: *The authors have declared that no competing interests exist.*<br><br>**Authors with competing interests**<br><br>Enter competing interest details beginning with this statement:<br><br>*I have read the journal's policy and the authors of this manuscript have the following competing interests: [insert competing interests here]*<br><br>* typeset	
**Ethics Statement**<br><br>Enter an ethics statement for this submission. This statement is required if the study involved:<br><br>• Human participants<br>• Human specimens or tissue<br>• Vertebrate animals or cephalopods<br>• Vertebrate embryos or tissues<br>• Field research	Protocol No: 049/20/SPH<br><br>Institutional Review Board of the College of Health Sciences, Addis Ababa University

Powered by Editorial Manager® and ProduXion Manager® from Aries Systems Corporation

Write "N/A" if the submission does not require an ethics statement.

General guidance is provided below. Consult the submission guidelines for detailed instructions. **Make sure that all information entered here is included in the Methods section of the manuscript.**

Format for specific study types

Human Subject Research (involving human participants and/or tissue)

- Give the name of the institutional review board or ethics committee that approved the study
- Include the approval number and/or a statement indicating approval of this research
- Indicate the form of consent obtained (written/oral) or the reason that consent was not obtained (e.g. the data were analyzed anonymously)

Animal Research (involving vertebrate animals, embryos or tissues)

- Provide the name of the Institutional Animal Care and Use Committee (IACUC) or other relevant ethics board that reviewed the study protocol, and indicate whether they approved this research or granted a formal waiver of ethical approval
- Include an approval number if one was obtained
- If the study involved *non-human primates*, add *additional details* about animal welfare and steps taken to ameliorate suffering
- If anesthesia, euthanasia, or any kind of animal sacrifice is part of the study, include briefly which substances and/or methods were applied

Field Research

Include the following details if this study involves the collection of plant, animal, or other materials from a natural setting:

- Field permit number
- Name of the institution or relevant body that granted permission

Powered by Editorial Manager® and ProduXion Manager® from Aries Systems Corporation

**Data Availability**<br><br>Authors are required to make all data underlying the findings described fully available, without restriction, and from the time of publication. PLOS allows rare exceptions to address legal and ethical concerns. See the PLOS Data Policy and FAQ for detailed information.<br><br>A Data Availability Statement describing where the data can be found is required at submission. Your answers to this question constitute the Data Availability Statement and **will be published in the article**, if accepted.<br><br>**Important:** Stating 'data available on request from the author' is not sufficient. If your data are only available upon request, select 'No' for the first question and explain your exceptional situation in the text box.<br><br>Do the authors confirm that all data underlying the findings described in their manuscript are fully available without restriction?	Yes - all data are fully available without restriction
**Describe where the data may be found in full sentences. If you are copying our sample text, replace any instances of XXX with the appropriate details.**<br><br>• If the data are **held or will be held in a public repository**, include URLs, accession numbers or DOIs. If this information will only be available after acceptance, indicate this by ticking the box below. For example: *All XXX files are available from the XXX database (accession number(s) XXX, XXX.*).<br>• If the data are all contained **within the manuscript and/or Supporting Information files**, enter the following: *All relevant data are within the manuscript and its Supporting Information files.*<br>• If neither of these applies but you are able to provide **details of access elsewhere**, with or without limitations, please do so. For example:	All relevant data are within the manuscript and its Supporting Information files.

Data cannot be shared publicly because of [XXX]. Data are available from the XXX Institutional Data Access / Ethics Committee (contact via XXX) for researchers who meet the criteria for access to confidential data.<br><br>*The data underlying the results presented in the study are available from (include the name of the third party and contact information or URL).*<br>• This text is appropriate if the data are owned by a third party and authors do not have permission to share the data.<br><br>* typeset	
Additional data availability information:	

This is the Authors statement

Cover Letter

Trends in non-pharmaceutical intervention (NPI) related community practice for the prevention of COVID-19 in Addis Ababa, Ethiopia

What this study adds

Our study involved a timely event that targets a pro-active policy decision for the prevention of ~~Covid-19.~~ We used a non-participatory method of observation that helps to collect valid data at population level. ~~An observation on weekly~~ basis involving dedicated sites with public services ~~is hoped~~ to show the trend of the progress that can be with a mitigation ~~proactive measure~~. ~~This is realistic tool where vaccines are not or limited in countries.~~

~~This study was the mandate of the academia, whose outcomes is~~ planned to be used by the Ministry of Health here in Ethiopia. The study results were ~~greatly~~ acknowledged and ~~raised a fund~~ from the Ministry to continue the monitoring of personal protective behaviors up to present time. The Ministry is using the ~~out come of data~~ for social mobilization and ~~consider as~~ inputs for modelling the transmission.

~~Authors ensure that our study have not~~ previously ~~been~~ published, either in whole or in part (unless as an abstract), and ~~that~~ no similar paper is in press or under review elsewhere. The authors do not have any financial competing interests and ~~any~~ conflicts in this publication with any other person or institution. We ensure also that we do not have any closely related paper with this submission.

Source of Fund: The data collection was funded by the School of Public Health of Addis Ababa University. The Authors have contributed professionally ~~in view of supporting~~ the Ministry of Health in enhancing proactive informed decisions.

Authors list: Damen Haile Mariam[1], Abera Kumie[1], Samson Wakuma[1], Yifoker Teferra[1], Teferi Abegaz[1], Worku Tefera[1], Wendimu Ayele[1], Mulugeta Tamire[1]

[1] School of Public Health, College of Health Sciences, Addis Ababa University

Manuscript

Click here to access/download;Manuscript;PLOS Manuscript_Revised _DH3_AK.edited.docx

Research Article

Trends in non-pharmaceutical intervention (NPI) related community practice for the prevention of COVID-19 in Addis Ababa, Ethiopia

Authors List

Damen Haile Mariam[1], Abera Kumie[1*], Samson Wakuma[1], Yifoker Teferra[1], Teferi Abegaz[1], Worku Tefera[1], Wendimu Ayele[1], Mulugeta Tamire[1,] Shibabaw Yirsaw[1]

[1] School of Public Health, College of Health Sciences, Addis Ababa University

Corresponding Author

*aberakumie2@yahoo.com

// Abstract

Background: The COVID-19 pandemic ~~had~~ affected Ethiopia since March 13, 2020, when the first case was detected in Addis Ababa. Since then, the incidence of cases has continued to increase day by day. As a result, the health sector has recommended universal preventive measures to be practiced by the public. However, studies on adherence to these preventive measures are limited ~~to inform evidence-based policy-making interventions.~~

Objective: To monitor the status of preventive practices of the population related to hand washing, physical distancing, and respiratory hygiene practices at selected sites within the city of Addis Ababa.

Methods: ~~A~~ weekly cross-sectional non-participatory observation ~~was~~ done during the period of April-June, 2020. Data was collected using Open Data Kit (ODK) tool in ten public sites involving eight public facilities targeted for individual observations. Ten individuals were randomly observed at each facility over two days a week at peak hours of public services. WHO operational definitions of the preventive behaviors were adopted for this study. Observations were anonymously ~~fixed~~ at gates or entrances of public facilities and places.

Results: A total of 12,056 individual observations with 53% males and 82% in 18-50 years age group were involved in this study. There was an increase in the practice of respiratory hygiene ~~(~~from 14% in week one to 77% in week 10~~)~~, while those of hand hygiene and physical distancing changed little over the weeks from their baseline of 24% and 34%, respectively. Overall, respiratory hygiene ~~has shown~~ an increased rate of 6% per week, while hand hygiene and

physical distancing had ~~<1%~~. Females and age group 18-50 years had ~~increased~~ practice ~~in~~ respiratory hygiene with no difference in hand hygiene and physical distancing. Respiratory hygiene took about six weeks to reach a level of 77% from its baseline of 24%, making an increment of about 9% per week.

Conclusion: The public practice of respiratory hygiene ~~was relatively better than hand hygiene and physical distancing. It has shown visible weekly changes towards an increasing trend.~~ Regularly sustained public mobilization and mass education are required to sustain the achievements gained in respiratory hygiene and further ~~improving~~ hand hygiene and physical distancing.

Keywords: COVID-19, hand hygiene, physical distancing, respiratory hygiene, mask use.

Introduction

The COVID-19 pandemic ~~is assumed to originate from~~ Wuhan, China, in Dec 2019 [1]. Since then, it has spread to Europe, the USA, and later to Africa. The infection's nature of transmission ~~is shown~~ to be through droplets~~, aerosols,~~ and contact transmission (2). The possibility of transmission through faeco-oral and airborne routes is still under investigation [2,3]. Pre-symptomatic cases facilitate ~~most of~~ the transmission for a household clustering of infection and during travel~~s~~ [4-6]. Symptomatic infections ~~were having~~ the highest transmission potential compared to asymptomatic and presymptomatic when considering the transmission index [7]. ~~This is in a way to say known COVID-19 cases with symptoms have the greatest transmission potential if left uncontrolled the source of transmission. While asymptomatic and pre-symptomatic cases involve unnoticed silent transmissions.~~ The mortality pattern is devastating among elder age groups because of limited body immunity [8,9].

Infection prevention and control (IPC) measures for emerging infectious diseases, like COVID-19~~,~~ in health care settings can be ~~managed with difficulty and confusion,~~ especially in outbreaks where resources are limited [10]. Yet such measures, including early identification, prompt isolation, proper patient placement, adequate space ventilation, and proper use of personal protective equipment, are important in preventing and controlling the transmission of COVID-19 [11]. In addition, community prevention activities are considered the best strategies to reduce the ~~healthcare burden, as they help slow~~ transmission of the virus in ~~a~~ population [12]. Community prevention strategies are a set of actions that persons and communities can implement to slow the transmission of COVID-19 by practicing hand hygiene, respiratory hygiene, and social distancing [13].

The Ministry of Health of Ethiopia provides daily updates on the number of new infections and reported mortalities because of COVID-19 (https://www.worldometers.info/coronavirus/country/ethiopia/). The first case in Ethiopia was declared on March 13, 2020, and the number of cases grew very slowly until the end of the first week of May 2020 (from 1 case on March 13 to 194 cases on 08 May 2020) [14]. However, the number of reported cases increased since then, reaching 110,074 cases and 1,706 deaths on 01 December 2020 (the time of the write-up of this manuscript) [13].

In Ethiopia, the Federal Ministry of Health (FMOH) and the Ethiopian Public Health Institute have created awareness about the virus transmission and prevention method using different approaches, including mass media such as nation TVs and radios during the partial-lock down period of five months since April, 20/2021. The interventions taken by the government ranged from banning public gatherings (over four persons) to limiting the number of passengers on public transport. In addition, all public service rendering institutions (such as food establishments, marketplaces, banks, and health facilities) provided hand-washing facilities and maintained a physical distance of at least one meter between their clients. Furthermore, security forces and public authorities have shown direct involvement in maintaining mask use and keeping physical distance in public places such as marketplaces, public transport stations and terminals, and streets. Land borders were shut in view of controlling the entrance of the virus abroad. These interventions have proven the decline of COVID-19 transmission in other countries [15] that present the only effective ones in the absence of vaccines and medications [16]. Data on monitoring the status of these interventions, however, is lacking in Ethiopia.

~~This monitoring~~ generates evidence on COVID-19 prevention practices of the residents of Addis Ababa (focusing on hand hygiene, physical distancing, and respiratory hygiene). The findings are expected to inform policy to sustain the related behaviors in the general population.

Methods

Study design, Study Area, Setting, and Period

A ~~weekly~~ cross-sectional observational design was conducted ~~during~~ April 24-June 28, 2020. Addis Ababa is the capital city of Ethiopia and is headquarters for the African Union and the Economic Commission for Africa. The city ~~has five mln in~~ Dec 2021 [17] and is sub-divided into ten administrative sub-cities. Subcities are divided into districts (“Wereda”). One of the biggest markets in Africa (“*Merkato*”) is located in the heart of the city. There are about 131 Orthodox churches as well as several mosques. About 67% of the total vehicles (estimated to be about 800,000) are registered within Addis Ababa. There are several public and private banks in the city – including the Commercial Bank of Ethiopia (a state enterprise) that alone has over 120 branches in Addis Ababa.

This study was initiated as part of the Emergency Operation Center (EOC) preventive behaviour monitoring activity at the College of Health Sciences (CHS), Addis Ababa University. CHS owns a teaching hospital that ~~required~~ an epidemic preparedness and management plan for its staff and visiting patients. The monitoring aimed to explore community practice ~~level~~ regarding the three non-pharmaceutical interventions (NPI) for COVID-19 prevention ~~(~~hand hygiene, physical distancing, and respiratory hygiene~~)~~ in the ten administrative units (sub-cities) of Addis Ababa. The study ~~has been~~ conducted by observing individual’s behavior at selected sites when getting public services. These sites were selected based on the level of crowding and population mobility, and thus ~~considering~~ an indication of the risk for COVID-19 transmission.

Study population

The source population for the study was all individuals visiting the selected sites during the day of data collection. Study participants were individuals who visited the selected sites during the time of data collection.

Monitoring protocol

Before the data collection, a monitoring protocol has been prepared and approved by the research committee. This is a standard operative procedure (SOP) used for the application of data collection. The protocol describes the observation site selection, identifies the list of public facilities to be observed, operationally defines the NPI practices, and the time and the procedure for the observation. First, “community center-public sites' were identified in each sub-city. These sites are characterized by having increased population mobility relative to the nearby neighborhoods. Then, one of the relevant sites was identified as a nucleus for the subsequent selection of government and public institutions and facilities for monitoring. Eight facility-sites were purposely selected based on selection criteria that included active public service provision, availability of adequate flow of service users, and appropriateness of the location for observation relative to increased population mobility. These sites are religious places, health facilities, market places, banks, public transport, food and drink establishments, street crossing sites, and workplaces. Each public facility had ten observations.

We followed the protocol’s provision in standardizing the context of observation. Fixing the distance of fewer than 10 meters from the observation point represented by the gate or entrance to the public services (buildings or open spaces like a traditional market) was kept. This is the distance that maintains the proximity of observation by the research assistant. The observation of

an individual seeking a public service was designed anonymously to get valid behaviours of an individual's practice in reference to hand hygiene, physical distance, and respiratory hygiene. Ethical approval was obtained for this purpose. No names or photos were required for observation. The research assistant finds a convenient place that allowed his visual field to be optimum for an observation. He used a mobile phone ODK to record data without being identified by any person. This helps to distract suspecting his work, hence maintaining neutrality and data validity. He kept moving within the proximity of the gate in case there might be the discomfort of being identified. The research assistant was instructed to spend about 15-30 minutes on one site, which allowed him not to be identified easily. Finally, only two days in a week, weekdays (Wednesdays) and weekends (Sundays) were used for data collection to have weighted data represented by working days and holidays. The provisions of the context of monitoring are indicated in the support information (S1)

Data collection tools

We prepared a data collection tool that had two sub-sections (attached in a supplementary file). Section 1 addressed the location characteristics, while section 2 records the practice of the individual. The location checklist addressed information regarding the time of observation, the Global Positioning System (GPS) of each service facility location, the presence of any enforcement, and the availability of handwashing facilities, including water and detergents. The NPI practice section contained sex, estimated age, NPI practice in three categories (proper, improper, and none). Each NPI practice was operationally defined within the monitoring protocol following the WHO recommendation [18] (S2).

Data collection procedure

One trained research assistant was assigned per sub-city, and one supervisor was appointed for two data collectors. The supervisor ~~used to communicate~~ with the field research assistant on each day of data collection to discuss the context and challenges of data collection. The field data were collected using the Open Data Kit tool (ODK) at the peak hours when the number of people was assumed to be highest at each observation site stated in the protocol (Fig 1). The ~~daily~~ recorded ODK data were uploaded daily to the central server.

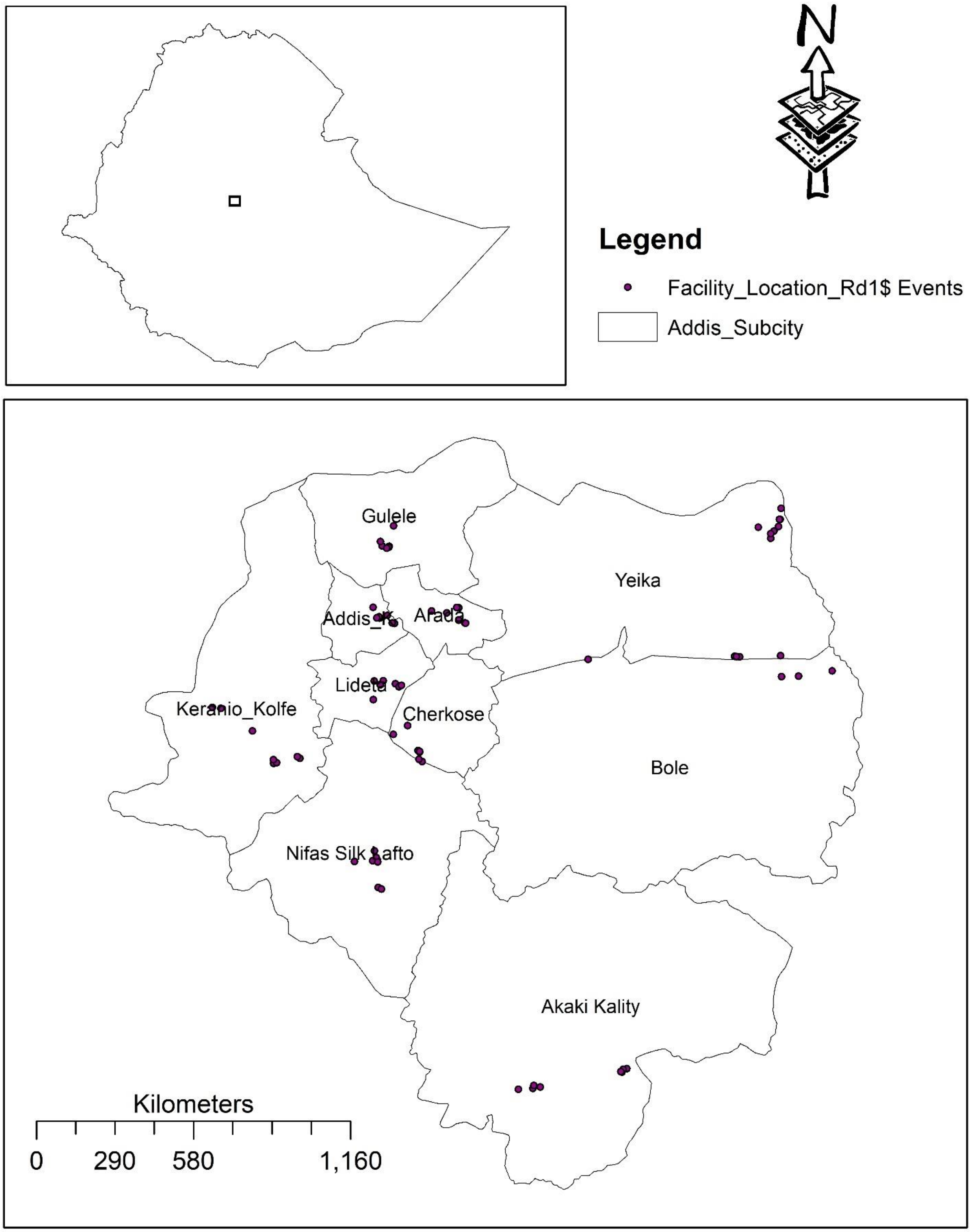

Figure 1: Observational data collection sites in Addis Ababa

Data management and analysis

Data from the main server were downloaded ~~in excel format~~ and transferred into SPSS V 23 for data merging, cleaning, and analysis. Data were presented using descriptive statistics by calculating the proportion of practices for each week. The trend of the practice was evaluated using weekly differences in proportions (a current week with the previous week). The long-term trend was evaluated by visualizing the line graph and looking at the overall average of weekly differences. Limited statistical tests were done to see the significance of the overall practice with sex and ~~age group~~. The weekly difference in the three practices was checked using a non-parametric ~~test of~~ Kruskal Wallis. The average of differences between each week was considered to estimate the strength of the overall weekly change. Tables, line graphs, and charts were used to present data. The data set is in the ~~support~~ information (S3).

Ethical considerations

The study ~~has~~ ethical approval from the Institutional Review Board (IRB) of the College of Health Sciences at Addis Ababa University. We had permission from IRB not to have consent as the results of ~~observation~~ were meant to benefit the public at large and we did not seek any ~~identifiers and photos of an individual~~ (S4). The survey ~~uses~~ a random observation tool that does not override the rights of any individual or institution, and the observation ~~is~~ done purely anonymously. A weekly report on the progress of monitoring was submitted to the Ministry of Health of Ethiopia to help to take proactive actions.

Results

Study participants

A total of 12,056 people were observed from April 20 to June 28, 2020. More than half of these (59.2%) were observed on the weekdays (Table 1). We had males 6,383 (52.9%) and females 5,673 (47.1%) in the study. The majority, 9832 (81.6%), were in the estimated age group of 18-50 years.

Table 1: Number of people observed (April 20– June 14, 2020, Addis Ababa)

Observation week	Weekdays observations # (%)	Weekend observations # (%)	Total observations # (%)
Week 1 (Apr20-26)	225 (26.3)	629 (73.7)	854 (7.08)
Week 2 (Apr27-May3)	723 (62.1)	442 (37.9)	1,165 (9.66)
Week 3 (May 4-May 10)	804 (63.6)	460 (36.4)	1264 (10.48)
Week 4 (May 11-May 17)	719 (60.0)	480 (40.0)	1,199 (9.95)
Week 5 (May 20- May 24)	775 (62.2)	470 (37.8)	1,245 (10.33)
Week 6 (May 27-May 31)	706 (59.1)	488 (40.9)	1,194 (9.90)
Week 7 (June 3-June 7)	799(62.0)	489 (38.0)	1,288 (10.68)
Week 8 (June 10-June 14)	788(61.7)	490 (38.3)	1,278 (10.60)
Week 9 (June 15- June 21)	802 (62.6)	479 (37.4)	1,281 (10.63)
Week 10 (June 22- June 28)	798 (62.0)	490 (38.0)	1,288 (10.68)
**Total**	**7,139 (59.2)**	**4,917 (40.8)**	**12,056 (100)**

The overall trend of community practice

Surgical masks predominated, 51% relative to cloth masks, 41%, N95, 4%, and scarf, 4%. Proper hand hygiene in the community slowly increased from the baseline of 24.2% in week one to 32.5% in week three and then slowly declined to 23.7% in week 10. Similarly, proper physical distancing in the community increased from 34.4% in week one to 42.6 % in week four and then slowly declined to 35.2 % in week eight. On the contrary, proper respiratory hygiene in the community continuously increased from 14% in week one to 77% in week ten.

Driver's respiratory hygiene continuously increased from 13.8 % in week one to 84.6 % in week 10. Driver's assistants had 0% respiratory hygiene practice in week one to 70.5% in week six, then slowly declined to 54.1% in week 10 (Table 2). The results also showed that adherence to the required vehicle occupancy less than or equal to 50% occupancy rate in commercial vehicles declined from 88.8% in week one to 68.8 % in week 10. There was a sharp rise in the practice of respiratory hygiene, while those of hand hygiene and physical distancing did not change or declined during the last week of observation (Fig 2). Week 4 was the turning point to observe an ~~increasing change,~~ while the highest mask used in a population took seven weeks. The trend in hand hygiene and physically distancing through the ten weeks was stable relative to respiratory hygiene.

The three combined hand and respiratory hygiene practices with physical distancing managed by an individual were below 10% in any week. It increased slowly from 4.7% in week one to 10.8% in week four and then declined to 8.1% in week 10.

Varation in practices

Overall, we ~~have~~ found ~~the within observations~~ ~~overtime~~ statistical differences for the three practices ($p<0.05$), and within-subject differences by sex, age, and day of observation ($P<0.05$). Females and age category of 18-50 years had relatively ~~increased practice for~~ respiratory hygiene. The practices observed in the week-day had an increased proportion over the week-end day ($p<0.05$) for hand and respiratory hygiene.

Table 2: Weekly pattern of a community practice of non-pharmaceutical interventions (Addis Ababa, 2020)

NPI	NPI Practices	Week 1 (Apr20-26)	Week 2 (Apr27-May3)	Week 3 (May 4-10)	Week 4 (May 11- 17)	Week 5 (May20- 24)	Week 6 (May 27-31)	Week 7 (June 3-7)	Week 8 (June10-14)	Week 9 (June15-21)	Week 10 June 22-28
Hand hygiene	**Proper hand hygiene**	**178 (24.2)**	**328 (33.1)**	**350 (32.5)**	**323 (32.0)**	**305 (28.8)**	**26 1 (25.9)**	**288 (26.1)**	**290 (26.8)**	**281 (28.5)**	**254 (23.7)**
	Improper hand hygiene	88 (11.9)	93 (9.4)	101 (9.4)	76 (7.5)	78 (7.4)	80 (7.9)	121 (10.9)	105 (9.7)	123 (12.4)	103 (9.6)
	No hand hygiene	469 (63.8)	571 (57.6)	625 (58.1)	610 (60.4)	674 (63.8	667 (66.2)	692 (62.8)	684 (63.4)	582 (59.1)	715 (66.7)
	**Total**	**735**	**992**	**1076**	**1009**	**1057**	**1008**	**1101**	**1079**	**986**	**1072**
Physical distance	**Proper physical distance**	**294 (34.4)**	**462 (39.7)**	**491 (38.8)**	**511 (42.6)**	**46 7 (37.5)**	**444 (37.2)**	**479 (37.2)**	**496 (38.8)**	**503 (39.3)**	**454 (35.2)**
	Improper physical distance	408 (47.8)	531 (45.6)	593 (46.9)	551 (46.0)	655 (52.6)	643 (53.9)	699 (54.3)	662 (51.8)	711 (55.5)	710(55.1)
	No physical distance	152 (17.8)	172 (14.8)	180 (14.2)	137 (11.4)	123 (9.9)	107 (9.0)	110 (8.5)	120 (9.4)	67 (5.2)	124(9.6)
	**Total**	**854**	**1165**	**1264**	**1199**	**1245**	**1194**	**1288**	**1278**	**1281**	**1288**
Respiratory hygiene	**Proper respiratory hygiene**	**205 (24.0)**	**301 (25.8)**	**405 (32.0)**	**575 (48.0)**	**746 (59.9)**	**845 (70.8)**	**985 (76.5)**	**980 (76.7)**	**999 (78.0)**	**995 (77.3)**
	Improper respiratory hygiene	79 (9.3)	174 (14.9)	213 (16.9)	271 (22.6)	151 (12.1)	153 (12.8)	198 (15.4)	214 (16.7)	208 (16.2)	201 (15.6)
	No respiratory hygiene	570 (66.7)	690 (59.2)	646 (51.1)	353 (29.4)	348 (28.0)	196 (16.4)	105 (8.2)	84 (6.6)	74 (5.8)	92 (7.1)
	**Total**	**854**	**1165**	**1264**	**1199**	**1245**	**1194**	**1288**	**1278**	**1281**	**1288**
Drivers' respiratory hygiene	**Proper respiratory hygiene**	**11 (13.8)**	**34 (23.0)**	**91 (55.2)**	**133 (78.7)**	**159 (84.6)**	**170 (89.5)**	**167(84.8)**	**165(82.9)**	**156(82.5)**	**168 (84.6)**
	Improper respiratory hygiene	1 (1.3)	11 (7.4)	15 (9.1)	11 (6.5)	23 (12.2)	17 (8.9)	29 (14.7)	30 (15.1)	30 (15.9)	27 (13.6)
	No respiratory hygiene	68 (85.0)	103 (69.6)	59 (35.8)	26 (15.3)	6 (3.2)	3 (1.6)	1 (0.5)	4 (2.0)	3 (1.6)	4 (2.0)
	**Total**	**80**	**148**	**165**	**170**	**188**	**190**	**197**	**199**	**189**	**199**
Driver assistants' respiratory hygiene	**Proper respiratory hygiene**	**0%**	**29 (19.6)**	**60 (37.3)**	**87 (52.1)**	**119 (62.6)**	**141 (70.5)**	**135 (69.9)**	**118 (60.5)**	**111 (59.0)**	**106 (54.1)**
	Improper respiratory hygiene	3(4.0)	19(12.8)	29 (18.0)	44 (26.3)	61 (32.1)	48 (24.0)	57 (29.5)	72 (36.9)	73 (38.8)	80 (40.8)
	No respiratory hygiene	72 (96.0)	100 (67.6)	72 (44.7)	36 (21.6)	10 (5.3)	11 (5.5)	1 (0.5)	5 (2.6)	4 (2.1)	10 (5.1)
	**Total**	**75**	**148**	**161**	**167**	**190**	**200**	**193**	**195**	**188**	**196**
Vehicle occupant capacity	**Less than or equal to 50% capacity**	**71 (88.8)**	**122 (83.0)**	**114 (69.1)**	**110 (65.1)**	**128 (68.1)**	**128 (67.4)**	**136(69.0)**	**140(70.4)**	**132(69.8)**	**137(68.8)**
	Greater than 50% capacity	9 (11.3)	25 (17.0)	51 (30.9)	59 (34.9)	60 (31.9)	62 (32.6)	61 (31.0)	59 (29.6)	57 (30.2)	62 (31.2)
	**Total**	**80**	**147**	**165**	**169**	**188**	**190**	**197**	**199**	**189**	**199**

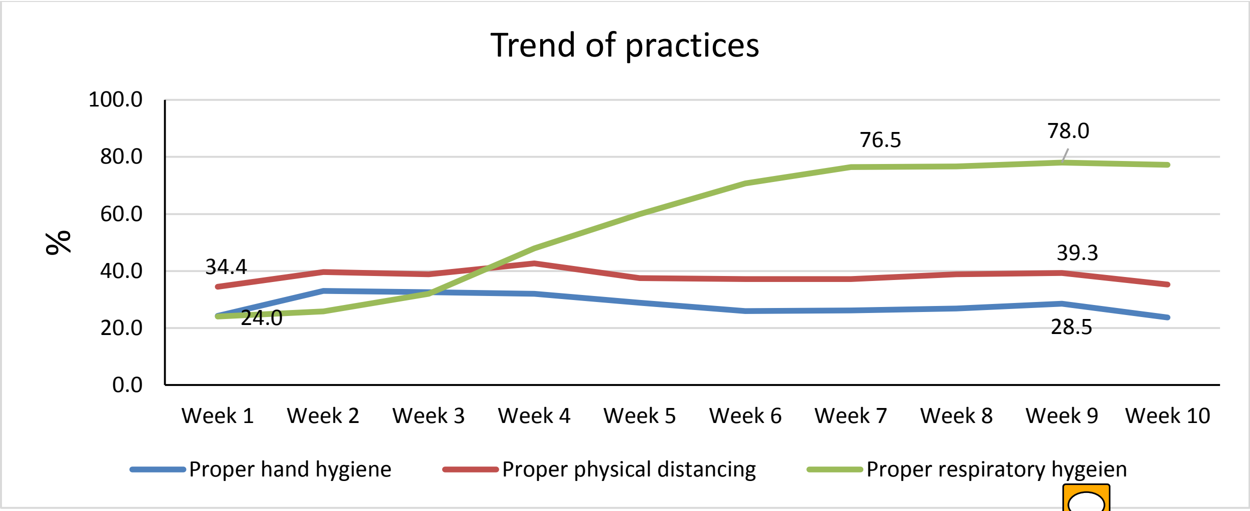

Figure 2: The overall trend of the proper community practices for the three ~~universal precautions~~

The trend of respiratory hygiene by observation facilities

There was a similar pattern of an increasing trend in all eight facilities with varying intensity of weekly ~~increasing~~. Banks and health facilities showed fast and stable growth rates over the ten weeks, from 30% in week one to 90% in week ten and from 30% in week 1 to 86% in week 10, respectively (Fig 3).

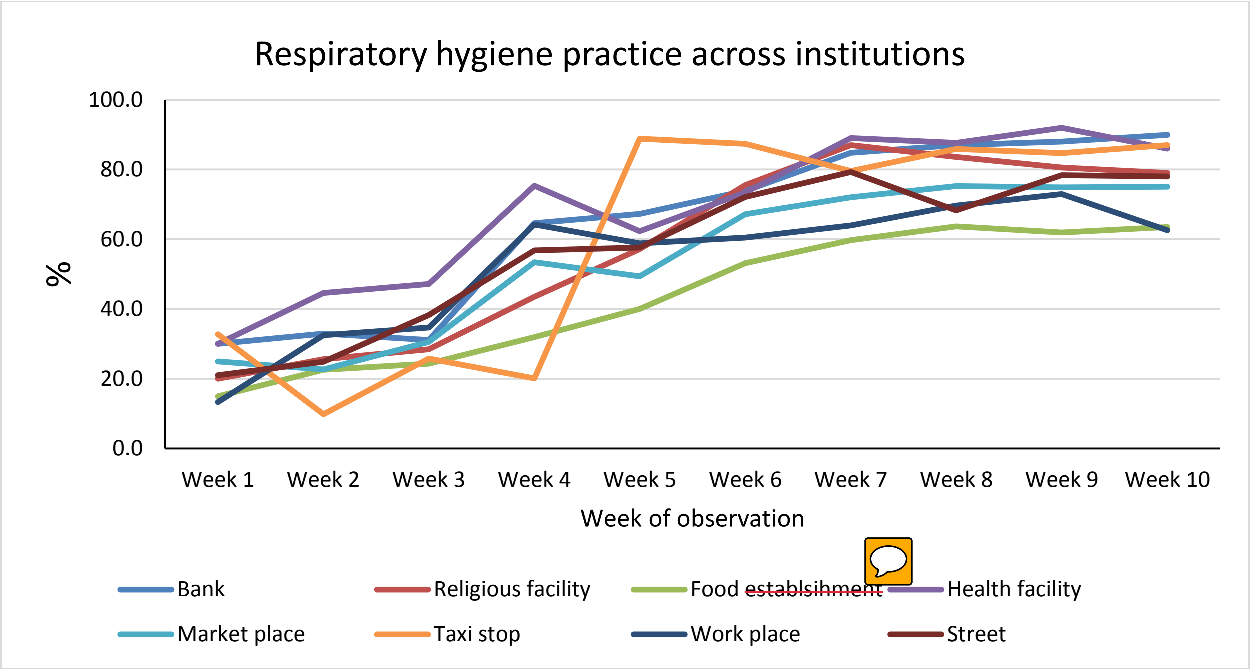

Figure 3: The trend of the proper respiratory hygiene practices by public facilities

Discussion

Non-pharmaceutical interventions are the primary preventions to restrict the spread of COVID-19 on the background of limited access to vaccines. Overall, our results demonstrated that the use of masks was stable at around 80%, while hand hygiene and physical distancing could not go beyond 40%.

The optimum application the three practices was limited in our study. This is only attained for the respiratory hygiene. The World Health Organization greatly acknowledged the combined practice of non-pharmaceutical interventions as a frontline tool to combat the infection [13,18] .The non-pharmaceutical interventions were effective in containing the outbreak demonstrated by the number of cases that could had been 67-fold higher at the time without these interventions [19]. Systematic reviews have shown the frequent practice of hand hygiene, the use of physical distancing of 1 m or more, and mask utilization have the potential benefit of lowering the COVID-19 transmission [20,21]. These preventions are effective with optimum coverage if not universal in a community as has been strongly proposed in China for a high-risk population [22]. The Nigerian Medical Association strongly condemned the limited use of non-pharmaceutical prevention protocols by citizens [23]. The low level of mask using was mainly attributed to risky social behaviors, perceived risk of transmission, gender and age differences [24]. This study also showed the variation of the mask use over time across many countries: some having gradually increased, others showing a declining trend. The community's access to affordable masks in our study should have provided a sense of protection compared to hand hygiene and physical distancing. The use of cloth masks that is easily accessible, cheap, and existing in many different options has provided a degree of protection next to surgical masks.

The present study has shown major improvement in the practice of respiratory hygiene compared to hand hygiene and physical distance during the period of our observation. The improvement started to show significant change since week five matched with when security forces started to enforce primary preventions in response to the state of emergency related to the epidemic crisis in Ethiopia. Although the state of emergency was declared on 20 April 2020, its impact came later, just after few weeks that have been used for massive promotional provisions targeting the public at large and public service rendering institutions in particular. At the time of the study, the State Emergency in Ethiopia was characterized to have partial lock-down of public places, including the closing of educational facilities and workplaces coupled with strict use of masks at public places, such as transport and public service places, which resulted in a rapid increase during our monitoring period. According to the state of emergency provisions, public mobility was not restricted to seeking food, transport, health care, and marketing services. There are reports from other countries that NPI interventions during lock-down periods are effective tools to interrupt COVID-19 transmission [25,26], although the benefit of physical distance and mask has been reported with uncertainties due to limited strong study designs [27]. The state of emergency mandated private and government service providers to avail handwashing facilities with detergents for their clients who seek services. Handwashing facilities are usually placed at the gate or entrance of the institution to encourage hand hygiene before accessing the service. The perception of self-protections by using masks might have contributed towards the increasing practice. On the contrary, a small portion of the public, about 20%, did not use a mask that might be linked to the negative attitudes towards mask ineffectiveness to reduce the transmission [28].

Despite the progressed public enforcement, the practice of proper hand hygiene did not show much change over the ten weeks of observation and could not go beyond 30%. Two reasons were indicated from our observation. Firstly, the media company and involvement of the enforcing volunteers had massive promotions in the first four weeks during our data collection period around churches, transport stations, and other public places. This has contributed to a change in handwashing practice in those weeks but not much beyond these weeks. On the other hand, the unavailability of water and soap could not be sustained in many facilities, except few public services providing institutions such as banks and health facilities. Generally, the provision of soap was challenged first, which was followed by the shortage and total disappearance of water in many public service providers. We believe these happened at the time of weak enforcement when security and other partners discontinued the initiated interventions. There was a shift from washing hands to using hand sanitizer by some members of the population, especially the youth. Our result had limited coverage of the youth; thus, we might have underestimated the proportion of handwashing in a community. The youths had limited seeking services in facilities that were involved in our study.

The maintenance of physical distancing at a minimum of one meter between any two individuals had a similar trend with handwashing practice. However, the former had an overall performance of below 40% relative to the 30% of the later practice. Physical distancing was challenged context-wise of social services, predominantly observed in open markets, walking and crossing on a street, and seeking public transport sectors. Individuals in an open market traditionally tend to cluster around a seller to make transactions. Seeking public transport services at peak hours makes service seekers get closer around the gate of transport vehicles. The duration of exposure and physical distancing has been shown an important predictor of the transmission of [redacted] [29]. A

systematic review of 172 studies reported that physical distancing of one meter was associated with a large reduction in the number of new infections in different countries [20]. Taking the economic status of Ethiopia, the lack of change in the physical distancing at the public level in this study was a missed opportunity to avert the occurrence of new cases in the city ~~given~~. Hand hygiene and physical distancing are effective, low-cost interventions that could ~~be reducing~~ the number of new cases [16,30]. We believe limited adherence in hand hygiene and physical distancing are missed opportunities to avert new infections in a community.

Ethiopia possesses traditions that ~~enhance~~ the community to get together, such as burial events, traditional religious festivals, attending churches, and traditional open markets. ~~State~~ emergency was not universal in the restriction of mass mobility during its active implementation phase, which might be a factor for the spread of ~~the~~ infection. Clients ~~are~~ advised to perform the three practices strictly during the initial wave of the epidemic. ~~The restriction became, however, a week after lifting off the state emergency.~~ There were incidences ~~when the~~ socio-cultural events ~~ignore~~ physical ~~distance~~ because of the population's crowding, such as religious holidays. ~~Inconsistent~~ with this finding, a study from China reported that almost all respondents avoided going to public places and reduced celebrating spring festivals due to the COVID-19 epidemic [31]. The possible reasons for the difference in adherence might be associated with the socio-cultural contexts in a given country and the degree of enforcing preventive practices.

The extent and trend of mask utilization had visible differences compared to physical distancing and hand hygiene. Mask utilization ~~has shown an~~ increasing trend until it ~~stabilizes~~ at about 80% towards the end of our study time. ~~Many factors operated.~~ The continued security enforcement,

mass media mobilization, the restrictions of public services without a mask, and the provisions of regular information about COVID-19 using national TVs and radios were the main contributing factors. The multiple sources of information and public enforcement were believed to enhance the attitude of service seekers, which greatly modified their behavior towards the use of a mask. We cannot deny that access to multiple types of masks, such as surgical, N95, and cloth, their local production at affordable prices has critically sustained the trend of mask utilization. The local production of face masks in low-middle income countries has been indicated as a strategy to contain the epidemic [32]. Before or after the state of emergency, the time of the observation and the age of study subjects could make a difference in the proportion of mask using. There is a preference of using masks among the young population in Poland, making the figure at 60%, a study undertaken before the government's enforcement [33], which is relatively lower than ours. The study in Poland was among the young population, which is not consistent with ours. Our study was undertaken after the State Emergency, which has brought multiple interventions to impact the behaviors of individuals in using a mask. Overall, the universal use of masks in public has been shown as an effective tool to reduce the transmission of COVID-19 in previous studies [34,35].

We disaggregated the mask used by type of facilities to get insights about the factors affecting mask using. The trend of respiratory hygiene practice visibly increased among service providers involving health care facilities, banks, and transport services compared with other facilities included in this study. There were assigned controllers and guards checking for the use of face-mask and maintenance of physical distancing and hand hygiene, who did not allow individuals to get a public service without these proper practices.

The relatively improved practices at bank and health facilities are related to the fact that these institutions have committed the resources to provide handwashing facilities with detergents and that their clients would be a relatively higher level of awareness and favourable attitude towards the preventive practices As there are variabilities in people's beliefs about the effectiveness as well as the rates of engagement in the use of non-pharmaceutical interventions, public health efforts should focus on increasing perceived severity and threats of the COVID-19 while promoting the preventive measures as effective tools [36,37] and enforcing the use of them as necessary. On the contrary, the adherence to mask using and physical distance in open markets and food establishments will continue to exist as a challenge. Food and drink establishments require close attention because people eat in groups, and physical distancing was almost not practically applied. Onsite dining with indoor and outdoor seats was identified as a risky situation by CDC[12].

This study has strengths because of observing individuals randomly and anonymously at times of service provisions during peak hours in key social providing services. This approach is believed to manage respondent's bias. The provision of weekly information to the Ministry of Health was an important input to enhance informed decisions to the public. We assumed observing individuals at the time of possible transmission in the presence of increased mobility and crowding provides better insights into the possibility of the transmission potential. We could not undermine the presence of interview bias that could happen over time, although we had regular supervisions and meetings with research assistants. The data representing only for a limited time observation could not represent the profile individual's behavior in a given day. This study cannot predict the trend in community practice in the absence of state wide interventions.

Conclusions and recommendations

The overall community prevention practices ~~of~~ COVID-19 pandemic were very low in Addis Ababa, Ethiopia, although respiratory hygiene singly showed a relatively increased rate. Despite the relatively low adherence of the community to proper handwashing and proper physical distancing, the success of proper respiratory hygiene was encouraging. ~~We call to strengthen~~ social mobilization using available media and consistent enforcement mechanisms to get sustained performances in community practices.

Acknowledgments: The authors are grateful to the College of Health Sciences and the School of Public Health of Addis Ababa University for allowing this study to happen. The leadership of Dr Dawit Wendimagegn, who is the Chief ~~e~~xecutive Director of the College of Health Sciences~~,~~ is greatly thanked for his support to undertake this study.

Competing interest: We do not have any competing financial interests or conflicts in this publication with any other person or institution.

Author's Contributions

DH tabled a research idea on the need to generate the status of community practice. AK was involved in the writing of the proposal and drafting the manuscript. AK and SW were involved in the analysis of data. MT was involved in handling the qualitative field information. WA was involved in the design of the data template in ODK. YT and TA were involved in the design of observation tools. SY was involved in coordinating the data collection. Finally, all were involved in editing the proposal and manuscript.

References

1. Chen X, Yu B (2020) First two months of the 2019 Coronavirus Disease (COVID-19) epidemic in China: real-time surveillance and evaluation with a second derivative model. Glob health res policy https://doiorg/101186/s41256-020-00137-4 5.

2. Beattie RM, Ashton JJ, Penman ID (2020) COVID-19 and the gastrointestinal tract: emerging clinical data. Frontline Gastroenterology 0: 1-3. https://fg.bmj.com/content/flgastro/early/2020/2004/2027/flgastro-2020-101507.full.pdf.

3. Liu J, Liao X, Qia nS, Yuan J, Wang F, et al. (2020) Community transmission of severe acute respiratory syndrome coronavirus 2, Shenzhen, China, 2020. Emerg Infect Dis 26.

4. Guoqing Q, Naibin Y, Ada HYM, Liping W, Guoxiang L, et al. (2020) COVID-19 Transmission Within a Family Cluster by Presymptomatic Carriers in China Clinical Infectious Diseases 71: 861-861.

5. Yu X, Yang R (2020) COVID-19 transmission through asymptomatic carriers is a challenge to containment. Influenza Other Respir Viruses 14: 474–475.

6. Qiu X, Nergiz AI, Maraolo AE, Bogoch II, Low N, et al. (2021) The role of asymptomatic and pre-symptomatic infection in SARS-CoV-2 transmission—a living systematic review. Clinical Microbiology and Infection 27: 511-519.

7. Chian WK, Naing L, Chaw L, Ali MR, Fathi MA, et al. (2020) What do we know about SARS-CoV-2 transmission? A systematic review and meta-analysis of the secondary attack rate and associated risk factors. PLOS ONE: https://doi.org/10.1371/journal.pone.0240205.

8. Dudley JP, Lee NT (2020) Disparities in Age-specific Morbidity and Mortality From SARS-CoV-2 in China and the Republic of Korea. Clin Infect Dis 71: 863-865.

9. Palaiodimos L, Kokkinidis DG, Li W, Karamanis D, Ognibene J, et al. (2020) Severe obesity, increasing age and male sex are independently associated with worse in-hospital outcomes, and higher in-hospital mortality, in a cohort of patients with COVID-19 in the Bronx, New York. Metabolism 108: 154262.

10. World Health Organization (2014) Infection prevention and control of epidemic- and pandemic-prone acute respiratory infections in health care. https://apps.who.int/iris/bitstream/handle/10665/112656/9789241507134_eng.pdf.

11. European Center for Diseases Prevention and Control (2021) Infection prevention and control and preparedness for COVID-19 in healthcare settings. https://www.ecdc.europa.eu/en/publications-data/infection-prevention-and-control-and-preparedness-covid-19-healthcare-settings.

12. Centers for Diseases Control and Prevention (2020) Implementation of Mitigation Strategies for Communities with Local COVID-19 Transmission. https://www.cdc.gov/coronavirus/2019-ncov/downloads/community-mitigation-strategy.pdf.

13. World Health Organization Corona virus disease (COVID-19) advice for the public.https://www.who.int/emergencies/diseases/novel-coronavirus-2019/advice-for-public. https://www.who.int/emergencies/diseases/novel-coronavirus-2019/advice-for-public.

.

14. Worldometers (2020) Ethiopia: Corona Virus Cases. https://www.worldometers.info/coronavirus/country/ethiopia/.

15. Pei S, Kandula S, Shaman J (2020) Differential Effects of Intervention Timing on COVID-19 Spread in the United States. medRxiv: 2020.2005.2015.20103655.

16. O M, F N, O R (2020) [Epidemiology and control of COVID-19] (Abstract). Dtsch Med Wochenschr 145: 670-674. doi 610.1055/a-1162-1987.

17. United Nations (2021) World Population Prospects. https://www.macrotrends.net/cities/20921/addis-ababa/population

18. World Health Organization (2020) Considerations for implementing and adjusting public health and social measures in the context of COVID-19. https://www.who.int/health-topics/coronavirus#tab=tab_2.

19. Shengjie L, Nick WR, Liangcai Z, Olivia Prosper, Wei L, Jessica RF, et al. (2020) Effect of non-pharmaceutical interventions to contain COVID-19 in China. Nature 585: 410-413.

20. Chu DK, Akl EA, Duda S, Solo K, Yaacoub S, et al. (2020) Physical distancing, face masks, and eye protection to prevent person-to-person transmission of SARS-CoV-2 and COVID-19: a systematic review and meta-analysis. Lancet 395: 1973-1987.

21. Jefferson T, Del Mar CB, Dooley L, Ferroni E, Al-Ansary LA, et al. (2011) Physical interventions to interrupt or reduce the spread of respiratory viruses. The Cochrane database of systematic reviews, 2011(7), CD006207. . Cochrane Database Syst Rev 7.

22. Joseph TFL, Xilin Y, Tsui HY, Ellie P (2004) SARS related preventive and risk behaviours practised by Hong Kong-mainland China cross border travellers during the outbreak of the SARS epidemic in Hong Kong. J Epidemiol Community Health 58: 988-996.

23. Alexander D (2020) NMA Condemns Non-Adherence To COVID-19 Prevention Protocols. https://consumerconnectng.com/8186.

24. Badillo-Goicoechea E, Chang T-HK, Esther, LaRocca S, Morris K, Deng X, et al. (2021) Global Trends and Predictors of Face Mask Usage During the COVID-19 Pandemic. arXiv https://arxivorg/ftp/arxiv/papers/2012/201211678pdf.

25. Yacong B, Cui G, Changqing L, Samuel YSW, Alexis KHL, et al. (2021) Effectiveness of non-pharmaceutical interventions on COVID-19 transmission in 190 countries from 23 January to 13 April 2020. International Journal of Infectious diseases 102: 247-251. https://pubmed.ncbi.nlm.nih.gov/33129965/.

26. Stutt R, Retkute R, Bradley M, Gilligan C, Colvin J (2020) A modelling framework to assess the likely effectiveness of facemasks in combination with 'lock-down' in managing the COVID-19 pandemic. Proceedings of the Royal Scociety. A 476: 20200376. http://dx.doi.org/10.1098/rspa.2020.0376.

27. Jefferson T, Del Mar CB, Dooley L, Ferroni E, Al-Ansary LA, et al. (2020) Physical interventions to interrupt or reduce the spread of respiratory viruses. Cochrane Database Syst Rev 11: CD006207.

28. Taylor S, Asmundson G (2021) Negative attitudes about facemasks during the COVID-19 pandemic: The dual importance of perceived ineffectiveness and psychological reactance. PLoS ONE 16(2. PLoS One 16: e0246317. https://doi.org/0246310.0241371/journal.pone.0246317.

29. Rea E, Laflèche J, Stalker S, uarda BK, Shapiro H, et al. (2007) Duration and distance of exposure are important predictors of transmission among community contacts of Ontario SARS cases. https://doi.org/10.1017/S0950268806007771. Epidemiology and infection 135: 914–921.

30. Dalton CB, Corbett SJ, Katelaris AL (2020) COVID-19: implementing sustainable low cost physical distancing and enhanced hygiene. Medical journal of Australia 212: 443–446.e441. https://doi.org/410.5694/mja5692.50602.

31. Global Times (2020) Beijing districts, companies to enter into a state of emergency: official. https://www.globaltimes.cn/content/1211098.shtml.

32. Missoni E, Armocida B, Formenti B (2020) Face Masks for All and All for Face Masks in the COVID-19 Pandemic: Community Level Production to Face the Global Shortage and Shorten the Epidemic. Disaster medicine and public health preparedness: 1-5. https://doi.org/10.1017/dmp.2020.1207.

33. Matusiak Ł, Szepietowska M, Krajewski PK, Białynicki-Birula R, zepietowski JC (2020) The use of face masks during the COVID-19 pandemic in Poland: A survey study of 2315 young adults. Dermatologic therapy: Advanced online publication. https://doi.org/10.1111/dth.13909.

34. Jefferson T, Del Mar C, Dooley L, Ferroni E, Al-Ansary LA, et al. (2009) Physical interventions to interrupt or reduce the spread of respiratory viruses: systematic review. Br Med J 339.

35. Zhang L, Peng Z, Ou J, Zeng G, Fontaine RE, et al. (2013) Protection by face masks against influenza A(H1N1)pdm09 virus on trans-Pacific passenger aircraft, 2009. Emerg Infect Dis 19.

36. Van den Broucke S (2020) Why health promotion matters to the COVID-19 pandemic, and vice versa. Health Promot International 35: 181-186. doi: 110.1093/heapro/daaa1042.

37. Shahnazi H, Ahmadi-Livani M, Pahlavanzadeh B, Rajabi A, Shoaib MH, et al. (2020) Shahnazi, H., Ahmadi-Livani, M., Pahlavanzadeh, B. et al. Assessing preventive health behaviors from COVID-19: a cross sectional study with health belief model in Golestan Province, Northern of Iran. Infectious Disease Poverty 0: https://doi.org/10.1186/s40249-40020-00776-40242.

Supporting information

S1 Study/Monitoring Protocol

S2 Data collection check list

S3 Data set in SPSS format

S4 IRB Approval

Figures and Tables are in one file
Click here to access/download;Figure;Figures and tables Plos 1.docx

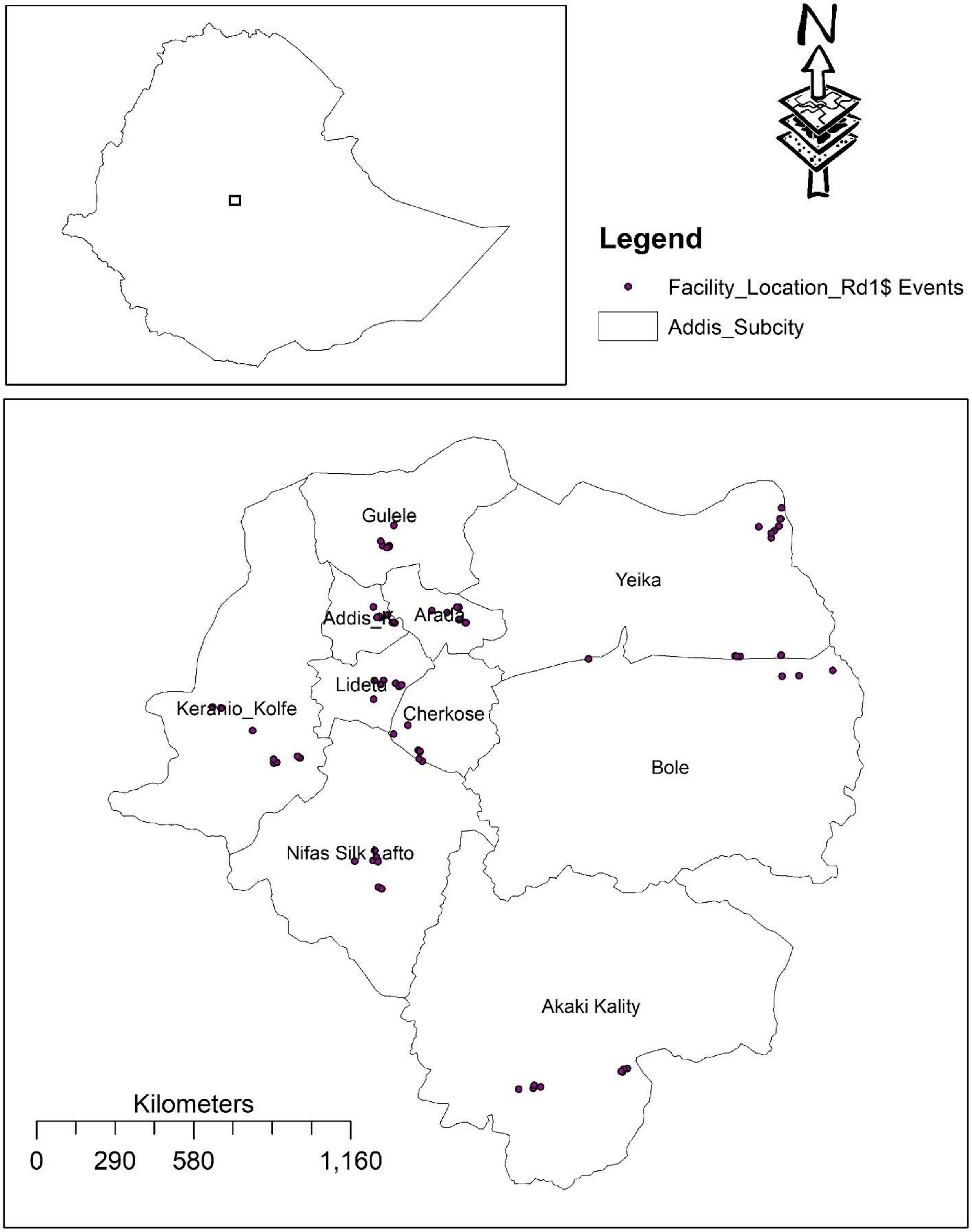

Figure 1: Observational data collection sites in Addis Ababa

Table 1: Number of people observed (April 20– June 14, 2020, Addis Ababa)

Insert Table 1

Observation weeks	Weekdays observations # (%)	Weekend observations # (%)	Total observations # (%)
Week 1 (Apr20-26)	225 (26.3)	629 (73.7)	854 (7.08)
Week 2 (Apr27-May3)	723 (62.1)	442 (37.9)	1165 (9.66)
Week 3 (May 4-May 10)	804 (63.6)	460 (36.4)	1264 (10.48)
Week 4 (May 11-May 17)	719 (60.0)	480 (40.0)	1199 (9.95)
Week 5 (May 20- May 24)	775 (62.2)	470 (37.8)	1245 (10.33)
Week 6 (May 27-May 31)	706 (59.1)	488 (40.9)	1194 (9.90)
Week 7 (June 3-June 7)	799(62.0)	489 (38.0)	1288 (10.68)
Week 8 (June 10-June 14)	788(61.7)	490 (38.3)	1278 (10.60)
Week 9 (June 15- June 21)	802 (62.6)	479 (37.4)	1281 (10.63)
Week 10 (June 22- June 28)	798 (62.0)	490 (38.0)	1288 (10.68)
**Total**	**7139 (59.2)**	**4917 (40.8)**	**12056 (100)**

Table 2: Demographic profile of participants by sub-city (April 20– June 14, 2020, Addis Ababa)

Variable	Total n (%) (n=12056)
Sex	
Male	6383 (52.9)
Female	5673 (47.1)
Age*	
<18 years	551 (4.6)
18-50 years	9832 (81.6)
>50 years	1673 (13.9)

*Age categories were made broad purposely in order to identify the closest age using observation without asking an individual

Table 3: Weekly pattern of community practice of non-pharmaceutical interventions (Addis Ababa, 2020)

NPI	NPI Practices	Week 1 (Apr20-26)	Week 2 (Apr27-May3)	Week 3 (May 4-10)	Week 4 (May 11- 17)	Week 5 (May20- 24)	Week 6 (May 27-31)	Week 7 (June 3-7)	Week 8 (June10-14)	Week 9 (June15-21)	Week 10 June 22-28
Hand hygiene	**Proper hand hygiene**	**178 (24.2)**	**328 (33.1)**	**350 (32.5)**	**323 (32.0)**	**305 (28.8)**	**26 1 (25.9)**	**288 (26.1)**	**290 (26.8)**	**281 (28.5)**	**254 (23.7)**
	Improper hand hygiene	88 (11.9)	93 (9.4)	101 (9.4)	76 (7.5)	78 (7.4)	80 (7.9)	121 (10.9)	105 (9.7)	123 (12.4)	103 (9.6)
	No hand hygiene	469 (63.8)	571 (57.6)	625 (58.1)	610 (60.4)	674 (63.8	667 (66.2)	692 (62.8)	684 (63.4)	582 (59.1)	715 (66.7)
	**Total**	**735**	**992**	**1076**	**1009**	**1057**	**1008**	**1101**	**1079**	**986**	**1072**
Physical ~~distance~~	**Proper physical distance**	**294 (34.4)**	**462 (39.7)**	**491 (38.8)**	**511 (42.6)**	**46 7 (37.5)**	**444 (37.2)**	**479 (37.2)**	**496 (38.8)**	**503 (39.3)**	**454 (35.2)**
	Improper physical distance	408 (47.8)	531 (45.6)	593 (46.9)	551 (46.0)	655 (52.6)	643 (53.9)	699 (54.3)	662 (51.8)	711 (55.5)	710(55.1)
	No physical distance	152 (17.8)	172 (14.8)	180 (14.2)	137 (11.4)	123 (9.9)	107 (9.0)	110 (8.5)	120 (9.4)	67 (5.2)	124(9.6)
	**Total**	**854**	**1165**	**1264**	**1199**	**1245**	**1194**	**1288**	**1278**	**1281**	**1288**
Respiratory hygiene	**Proper respiratory hygiene**	**205 (24.0)**	**301 (25.8)**	**405 (32.0)**	**575 (48.0)**	**746 (59.9)**	**845 (70.8)**	**985 (76.5)**	**980 (76.7)**	**999 (78.0)**	**995 (77.3)**
	Improper respiratory hygiene	79 (9.3)	174 (14.9)	213 (16.9)	271 (22.6)	151 (12.1)	153 (12.8)	198 (15.4)	214 (16.7)	208 (16.2)	201 (15.6)
	No respiratory hygiene	570 (66.7)	690 (59.2)	646 (51.1)	353 (29.4)	348 (28.0)	196 (16.4)	105 (8.2)	84 (6.6)	74 (5.8)	92 (7.1)
	**Total**	**854**	**1165**	**1264**	**1199**	**1245**	**1194**	**1288**	**1278**	**1281**	**1288**
Drivers' respiratory hygiene	**Proper respiratory hygiene**	**11 (13.8)**	**34 (23.0)**	**91 (55.2)**	**133 (78.7)**	**159 (84.6)**	**170 (89.5)**	**167(84.8)**	**165(82.9)**	**156(82.5)**	**168 (84.6)**
	Improper respiratory hygiene	1 (1.3)	11 (7.4)	15 (9.1)	11 (6.5)	23 (12.2)	17 (8.9)	29 (14.7)	30 (15.1)	30 (15.9)	27 (13.6)
	No respiratory hygiene	68 (85.0)	103 (69.6)	59 (35.8)	26 (15.3)	6 (3.2)	3 (1.6)	1 (0.5)	4 (2.0)	3 (1.6)	4 (2.0)
	**Total**	**80**	**148**	**165**	**170**	**188**	**190**	**197**	**199**	**189**	**199**
Driver assistants' respiratory hygiene	**Proper respiratory hygiene**	**0%**	**29 (19.6)**	**60 (37.3)**	**87 (52.1)**	**119 (62.6)**	**141 (70.5)**	**135 (69.9)**	**118 (60.5)**	**111 (59.0)**	**106 (54.1)**
	Improper respiratory hygiene	3(4.0)	19(12.8)	29 (18.0)	44 (26.3)	61 (32.1)	48 (24.0)	57 (29.5)	72 (36.9)	73 (38.8)	80 (40.8)
	No respiratory hygiene	72 (96.0)	100 (67.6)	72 (44.7)	36 (21.6)	10 (5.3)	11 (5.5)	1 (0.5)	5 (2.6)	4 (2.1)	10 (5.1)
	**Total**	**75**	**148**	**161**	**167**	**190**	**200**	**193**	**195**	**188**	**196**
Vehicle occupant capacity	**Less than or equal to 50% capacity**	**71 (88.8)**	**122 (83.0)**	**114 (69.1)**	**110 (65.1)**	**128 (68.1)**	**128 (67.4)**	**136(69.0)**	**140(70.4)**	**132(69.8)**	**137(68.8)**
	Greater than 50% capacity	9 (11.3)	25 (17.0)	51 (30.9)	59 (34.9)	60 (31.9)	62 (32.6)	61 (31.0)	59 (29.6)	57 (30.2)	62 (31.2)
	**Total**	**80**	**147**	**165**	**169**	**188**	**190**	**197**	**199**	**189**	**199**

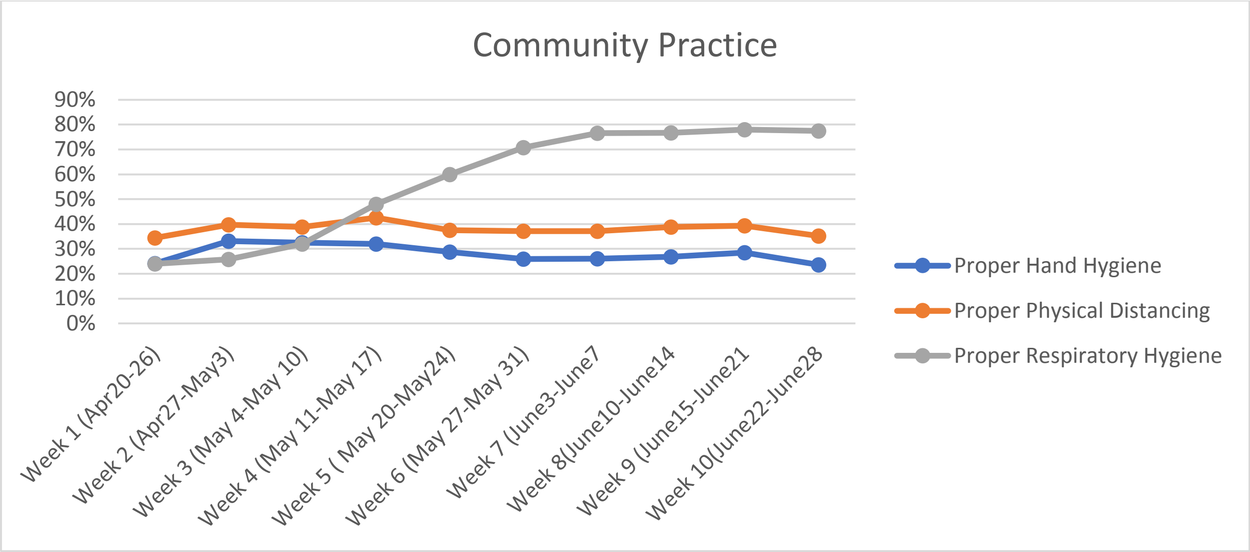

Figure 2: The overall trend of the proper community practices for the three ~~universal precautions~~

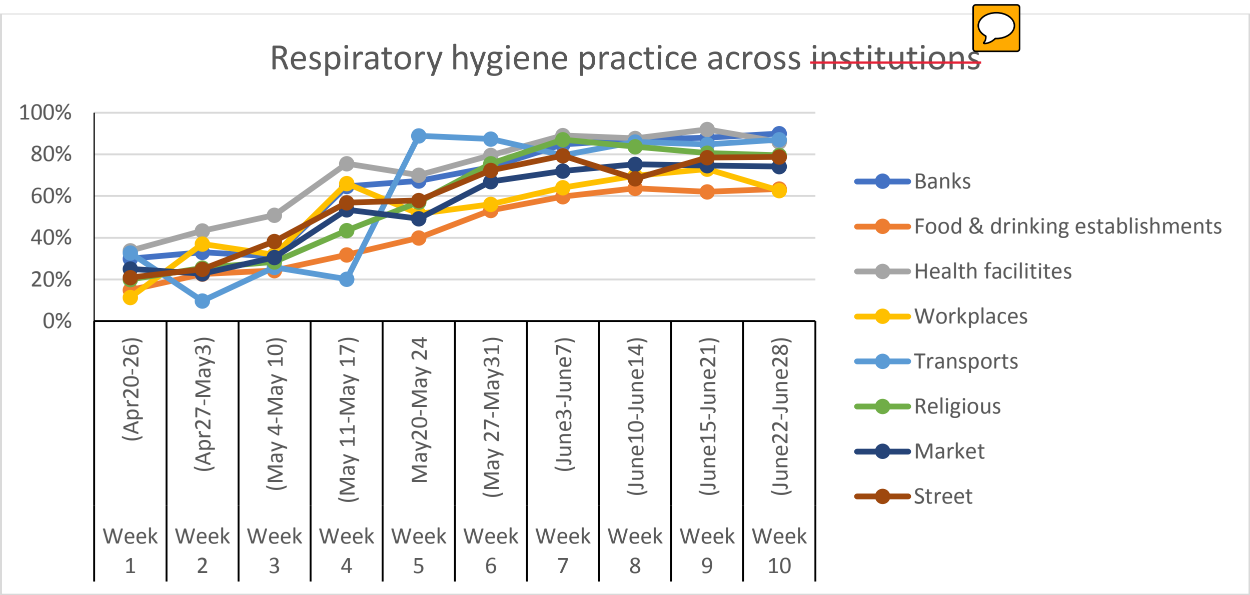

Figure 3: The trend of the proper respiratory hygiene practices at different institution sites

Table 4: Combined ~~differents~~ community practices

Risk category	Risk classification	Week 1 (Apr 20-26)	Week 2 (Apr 27-May 3)	Week 3 (May 4-10)	Week 4 (May 11-17)	Week 5 May 20-24	Week 6 May 27-31	Week 7 June 3-7	Week 8 June10-14	Week 9 June15-21	Week 10 June22-28	Total
**Proper hand hygiene & distancing & respiratory**	Low risk	40 (4.7)	79(6.8)	96 (7.6)	130 (10.8)	99 (8.0)	95 (8.0)	98 (7.6)	123 (9.6)	113 (8.9)	100 (7.8)	973 (8.1)
**Proper distance & respiratory**	Low risk	97 (11.4)	150(12.9)	206 (16.3)	264 (22.0)	311 (25.0)	334 (28.0)	396 (30.7)	405 (31.7)	413 (32.2)	383 (29.7)	2959 (24.5)
**Proper hand hygiene & distancing**	Medium risk	91 (10.7)	172(14.8)	174 (13.8)	180 (15.0)	149 (12.0)	118 (9.9)	116 (9.0)	140 (11.0)	130 (10.3)	110 (8.6)	1380 (11.5)
**Proper hand and resp hygiene**	Medium risk	58 (6.8)	127(10.9)	163 (12.9)	205 (17.1)	183 (14.7)	193 (16.2)	237 (18.4)	250 (19.6)	240 (20.2)	227 (17.8)	1883 (15.8)
**Only mask**	High risk	73 (8.5)	68(5.8)	90 (7.1)	165 (13.8)	276 (22.2)	328 (27.5)	348 (27.0)	375 (29.3)	290 (24.1)	381 (29.8)	2394 (20.0)
**Only physical distancing**	High risk	130 (15.2)	183(15.7)	181 (14.3)	176 (14.7)	87 (7.0)	68 (5.7)	50 (3.9)	54 (4.2)	59 (4.6)	47 (3.7)	1035 (8.6)
**Only hand hygiene**	High risk	69 (8.1)	108(9.3)	109 (8.6)	68(5.7)	72(5.8)	45(3.8)	33(2.6)	23(1.8)	24(1.9)	17(1.3)	568 (4.7)
**No hand & respiratory hygiene & no distancing**	High risk	305 (35.7)	350 (30.0)	375 [illegible]	248 (20.7)	211 (16.9)	161 (13.5)	162 (12.6)	141 [illegible]	124 (9.8)	169 (13.1)	2246 (18.7)

*Risk classification was made based on the level of [illegible]e practices at [illegible]ndividual lev[illegible]t is a relative term: an individual practicing ~~the~~ three is ~~in~~ low risk, while none of ~~only doing hand hygiene and physical distance in~~ high-risk group. ~~An individual with two combination~~ is ~~in the~~ medium risk.

Click here to access/download
Supporting Information
S2 Data Collection Checklist .docx

Click here to access/download
Supporting Information
S3 Data set SPSS.sav

Click here to access/download
Supporting Information
S1 Study protocol .docx

Click here to access/download
Supporting Information
S4 IRB approval letter_08June2020 .pdf

Original Article

Trends in non-pharmaceutical intervention (NPI) related community practice for the prevention of COVID-19 in Addis Ababa, Ethiopia

Authors List

Damen Haile Mariam[1], Abera Kumie[1], Samson Wakuma[1], Yifoker Teferra[1], Teferi Abegaz[1], Worku Tefera[1], Wendimu Ayele[1], Mulugeta Tamire[1]

[1] School of Public Health, College of Health Sciences, Addis Ababa University

Corresponding Author

Abera Kumie[1:] aberakumie2@yahoo.com; Mobile +251911-882912

Abstract

Background: The COVID-19 pandemic has affected Ethiopia since March 13, 2020, when the first case was detected in Addis Ababa. Since then, the incidence of cases continued to increase day by day. The health sector has recommended a set of universal preventive measures to be practiced by the community public. However, studies on adherence to these preventive measures is limited to inform evidence-based policy making interventions.

Objective:

To rapidly appraise and monitor the status of preventive practices of the population related to hand washing, physical distancing, and respiratory hygiene practices.

To rapidly appraise and monitor the preventive behavior of the population by conducting a series of observations on implementing the prescribed measures at selected sites within the city of Addis Ababa.

Methods: A weekly cross-sectional non-participatory observations was done during the period of April 24-June 28/2020. Data was collected using Open Data Kit (ODK) tool in ten public sites involving each 8 public facilities targeted for individual observations. Ten individuals were randomly observed at each facility over two days in a week at peak hours of public services. WHO operational definitions of the preventive behaviors were adopted for this study. Observations were anonymously fixed at gates or entrances of facilities that included health facilities, workplaces, banks, food establishments, religious institutions, public transport waiting areas, street crossing sites and market places.A repeated series of cross-sectional non-participatory observations for the preventive behaviors related to hand washing, physical distancing, and respiratory hygiene practices was done for the period of April 24-June 28/2020. Data were collected using Open Data Kit (ODK) tool in ten major sites involving each 8 public facilities targeted for individual observations. There were ten individuals who observed at each facility over two days in a week at peak hours of client's service. One week day and one weekend day were considered in order to observe a weighted outcome of the study. WHO operational definitions of the preventive behaviors were adopted for this study. Observations were fixed at gates and or entrances of public service providing facilities including health facilities, workplaces, banks, food establishments, religious institutions, public transport waiting areas, street crossing sites and market places.

Results: 12056 individual observations with 53% males, and 82% in 18-50 years age group were involved in this study. There was an increase in the practice of respiratory hygiene (from 14% in week one to 77% in week 10), while those of hand hygiene and physical distancing changed little over the weeks from their baseline of 24% and 34%, respectively. Overall, respiratory hygiene has shown an increasing rate of 6% per week, while hand hygiene and physical distancing had <1%. Females and age group 18-50 years had increased practice in respiratory hygiene with no difference in the two practices A total 12056 individual observations with 52% males, and 82% in 18-50 years

Formatted: Font: Not Bold

old age group were involved in this study. The results showed a sharp rise in the practice of respiratory hygiene (from 14% in week one to 77% in week 10), while those of hand hygiene (from 24.2% in week one to 23.7% in week 10) and physical distancing (from 34.4% in week one to 35.2% in week eight) changed little during the last weeks of observation. The three combined practices of hand and respiratory hygiene and physical distancing at individual level increased at a slow pace from 4.7% in week one to 8.1% in week 10.

Conclusion:

The public practice of respiratory hygiene was relatively better than hand hygiene and physical distancing. It has shown visible weekly changes towards an increasing trend. Public mobilization and regular mass education are required to sustain the achievements gained in respiratory hygiene and improving hand hygiene and physical distancing.

As adherence by the public to the preventive measures seems to be higher among those with relatively better awareness and during times of government mandates, mass education. The preventive measures need to be comprehensive with strict enforcement, as situations require.

Abstract

Background: The COVID-19 pandemic has affected Ethiopia since March 13, 2020, when the first case was detected in Addis Ababa. Since then, the incidence of cases continued to increase day by day. The health sector has recommended a set of universal preventive measures to be practiced by the public. However, studies on adherence to these preventive measures is limited to inform evidence-based policy interventions.

Objective:

To rapidly appraise and monitor the status of preventive practices of the population related to hand washing, physical distancing, and respiratory hygiene practices.

Methods: A weekly cross-sectional non-participatory observations was done during the period of April 24-June 28/2020. Data was collected using Open Data Kit (ODK) tool in ten public sites involving each 8 public facilities targeted for individual observations. Ten individuals were randomly observed at each facility over two days in a week at peak hours of public services. WHO operational definitions of the preventive behaviors were adopted for this study. Observations were anonymously fixed at gates or entrances of facilities that included health facilities, workplaces, banks, food establishments, religious institutions, public transport waiting areas, street crossing sites and market places..

Results: 12056 individual observations with 53% males, and 82% in 18-50 years age group were involved in this study. There was an increase in the practice of respiratory hygiene (from 14% in week one to 77% in week 10), while those of hand hygiene and physical distancing changed little over the weeks from their baseline of 24% and 34%, respectively. Overall, respiratory hygiene has shown an increasing rate of 6% per week, while hand hygiene and physical distancing had <1%. Females and age group 18-50 years had increased practice in respiratory hygiene with no difference in the two practices

Conclusion:

The public practice of respiratory hygiene was relatively better than hand hygiene and physical distancing. It has shown visible weekly changes towards an increasing trend. Public mobilization and regular mass education are required to sustain the achievements gained in respiratory hygiene and improving hand hygiene and physical distancing.

.

Introduction

The COVID-19 pandemic is assumed to originate from Wuhan, China on Dec 2019 (1). Since then, it has spread to Europe, USA, and lately to Africa. The nature of transmission of the infection is indicated to be through droplets, aerosols, and contact transmission (2). The possibility of transmission through faeco-oral and airborne routes is still under investigation (2, 3). Pre-asymptomatic cases facilitate most of the transmission (4, 5). The mortality pattern is devastating among elder age groups because of limited defensive mechanisms (6, 7).

The application of the infection prevention control (IPC) measures for emerging infectious diseases in health care settings can be managed with difficulty and confusion, especially in outbreaks where resources are limited (8). Yet such measures, including early identification, prompt isolation , proper patient placement, adequate space ventilation and proper use of personal protective equipment are reported to be important in the prevention and control for the transmission of COVID 19 (9). To reduce the burden on healthcare, community prevention activities are considered best strategies as they help slow transmission of the virus within communities (10). Community prevention strategies are a set of actions that persons and communities can implement to slow the transmission of COVID-19 by practicing hand hygiene, respiratory hygiene, and social distancing (11).

The Ministry of Health of Ethiopia is providing daily updates on the number of new infections as well as reported mortalities due to COVID-19. The first case in Ethiopia was declared on March 13, 2020, and the number of cases grew very slowly until the end of the first week of May 2020 (from 1 case on March 13 to 194 cases on 08 May 2020) (12). The number of reported cases continued to rapidly increase since then reaching 110,074 cases and 1,706 deaths on 01 December 2020 (the time of the write-up of this manuscript) (11).

In Ethiopia, the Ministry of Health (FMOH) and the Ethiopian Public Health Institute have created awareness about the virus transmission and prevention method to the public using different approaches including mass media. The interventions taken by the

government and communities range from banning of public gatherings (of more than four persons) to limiting the number of passengers in public transport. All public service rendering institutions (such as food establishments, market places, banks, and health facilities) are involved in providing facilities for hand washing and in maintaining physical distance of at least one meter between their clients. Security forces and public authorities have shown direct involvement in enforcing the maintenance of mask use and keeping physical distance in public places such as market places, public transport stations and terminals. These interventions have proven the declining of covid-9 transmission in other countries (13). Data on monitoring the status of these interventions, however, is lacking in Ethiopia.

The Thus, the purpose of this monitoring is to generate evidence on a weekly bases about the COVID-19 prevention practices of the residents of Addis Ababa (focusing on hand hygiene, physical distancing, and respiratory hygiene). The findings are expected to inform policy in order to sustain the related behaviors in thea general population.

Methods

Study design, Study Area, Setting, and Period

. A weekly cross-sectional observational design was conducted during the period of April 25 – June 28, 2020.

Addis Ababa is the capital city of Ethiopia, and is headquarters for the African Union and the Economic Commission for Africa. The city has a population of over four million, and is sub-divided into ten administrative sub-cities. One of the biggest markets in Africa ("*Merkato*") is located in the center of the city, and there are about 131 Orthodox churches as well as several mosques. About 67% of the total vehicles (estimated to be about 800,000) are registered within Addis Ababa. There are a number of state as well as private banks – the Commercial Bank of Ethiopia (a state enterprise) alone having more than 120 branches in Addis Ababa.

This study was initiated as part of the preventive behavior monitoring activity within the Emergency Operation Center (EOC) established at the College of Health Sciences, Addis Ababa University. It was aimed to ~~at~~ monitor~~ing~~ the level of community practice in terms of the three non-pharmaceutical interventions (NPI) for COVID-19 prevention (hand hygiene, physical distance, and respiratory hygiene). The study has been conducted by observing the behavior of individuals at selected sites at the time of getting ~~people in sites where they get various social and business-related services~~public ~~:~~services: religious places, health facilities, market places, banks, public transport, food and drink establishments, street crossing sites, as well as work places. These sites were selected based on level of crowding, and thus the level risk for COVID-19 transmission~~. A repeated cross-sectional observation design was conducted on weekly basis during the period of April 25 – June 28, 2020.~~

Study Population

The source population for the study were all individuals including children,visiting the selected sites. ~~, and s~~Study participants were individuals who visited ~~are found at~~ the selected sites during the time of data collection.

Monitoring Protocol

Prior to the data collection, a monitoring protocol has been prepared. The protocol describes the observation site selection, defines the observation facility, operationally defines the NPI practices, and the time as well as the procedure for the observation. First, "community center-public sites' were identified in each sub city. These sites are characterized by having increased population mobility relative to the nearby neighborhoods. Then, one of the sites are identified as a nucleus to identify government and public institutions and or facilities. Eight facility-sites were selected purposively based on selection criteria that included: the provision of public service functionalitybeing active, availability of service users, and appropriateness of the location for observation relative to increased population mobility. These sites are:

We followed the protocol's provision in standardizing the context of observation. Fixing the distance in less than 10 meters from the point of observation represented by the gate or entrance to the public services (could be buildings or open spaces like a traditional market) was kept. As the observation was designed anonymous without the consent of the study subject, we have adopted a strategy to consider the data collector should not bear any sign of observation about his job. He purposely seeks a convenient place to have a good vision for observation. He used a mobile phone (note pad) to enter the data. This helps to distract suspecting his work. At times, he kept moving around the gate in case there might be a discomfort od observation.

Data Collection ***T****ools*

"COVID-19 Prevention Practices Data Collection Tool in a community" tool with two sub sections was designed Two types of data collection tools were used for the study (attached in a supplementary file). Section 1 addressed the location characteristics, while section 2 records the practice of individual. – location observation checklist and individual NPI practice observation form. The location observation checklist was prepared to collect information regarding Global Positioning System (GPS) of each service facility location, presence of any enforcement, posted media related to COVID-19, and availability of hand washing facilities. The NPI practice observation tool contained information regarding the observed individuals' sex, estimated age, NPI practice in three categories (proper,

improper, and none). Each of the NPI practices were operationally defined within the monitoring protocol following the WHO recommendation (14). Include the defintions briefly

Formatted: Highlight

Data Collection Procedure

Data have been collected twice a week (one weekday and one weekend day) from each observation site identified in 10 sub-cities (zone). The duration of observation per each site is fixed for a minimum of 30 minutes. One data collector was assigned per each sub-city, and one supervisor appointed for two data collectors. The field data were collected using Open Data Kit tool (ODK) at the peak hour when the number of people is highest at each observation site stated in the protocol (Figure 1). The daily recorded data were uploaded daily to the central for data management on a weekly basis.

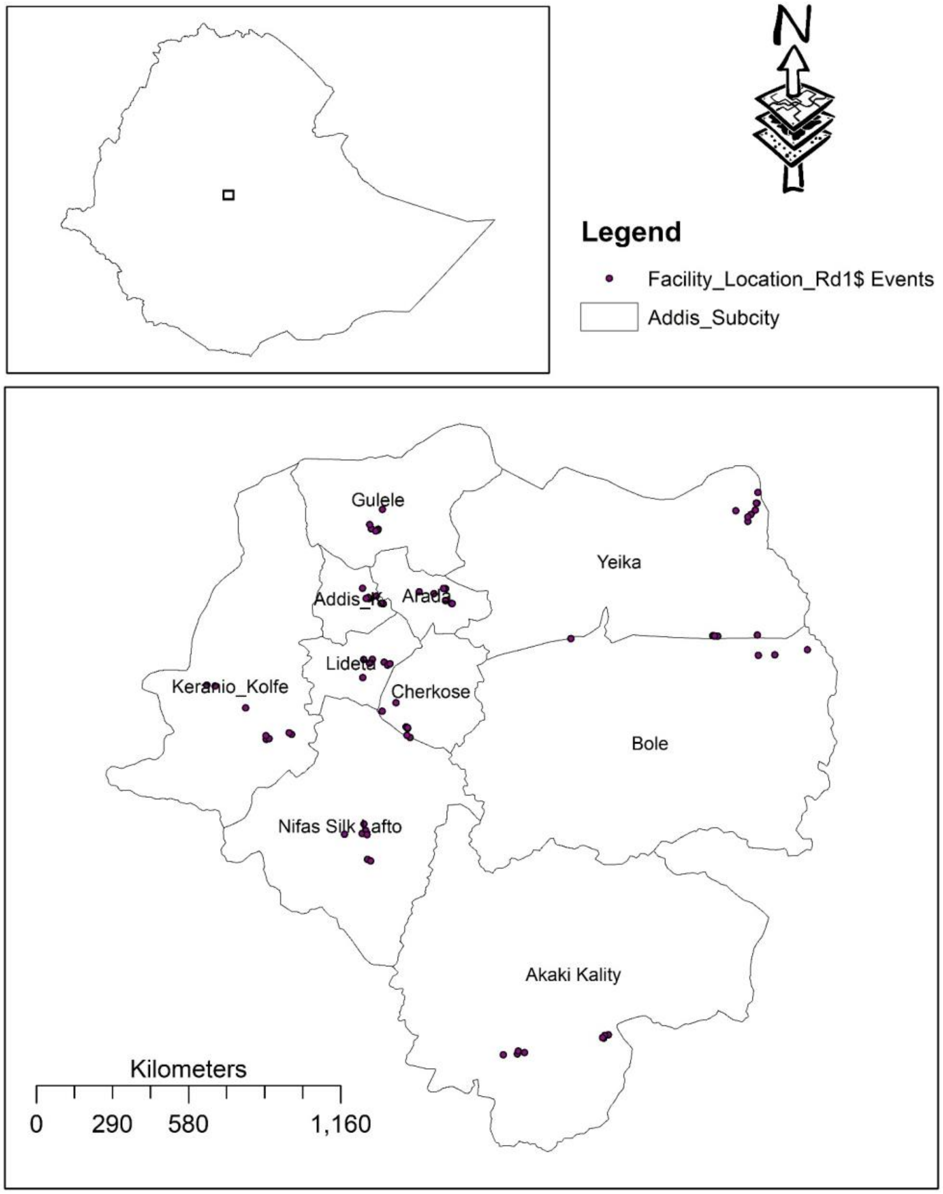

Figure 1: Observational data collection sites in Addis Ababa

Data management and analysis

The ODK based data was stored on a server on a database that was created for this purpose. Data was downloaded in excel format that was transferred into SPSS V 23 for data merging, cleaning, and analysis. Data is presented using descriptive statistics by calculating the proportion of practices by each week. The trend of the practice was evaluated using weekly differences in proportions that would indicate increasing with positive differences (current week with previous week,), The long term trend was evaluated by visualizing the line graph and looking at the overall average weekly differences. Limited statistical tests were done to see the significance of the overall practice with sex and age Tablesgroup. Tables, line graphs and Charts were managed to present the data.

Ethical Considerations

The study has got ethical approval from the Institutional Review Board of the College of Health Sciences at Addis Ababa University. The survey uses a random observation tool that does not override the rights of any individual or institution, and the observation is done purely anonymously.

Results

A total of 12,056 people were observed from April 20, 2020 to June 28, 2020. More than half of these (59.2%) were observed in the weekdays (Table 1).

Table 1: Number of people observed (April 20– June 14, 2020, Addis Ababa)

Observation weeks	Weekdays observations # (%)	Weekend observations # (%)	Total observations # (%)
Week 1 (Apr20-26)	225 (26.3)	629 (73.7)	854 (7.08)
Week 2 (Apr27-May3)	723 (62.1)	442 (37.9)	1165 (9.66)
Week 3 (May 4-May 10)	804 (63.6)	460 (36.4)	1264 (10.48)
Week 4 (May 11-May 17)	719 (60.0)	480 (40.0)	1199 (9.95)
Week 5 (May 20- May 24)	775 (62.2)	470 (37.8)	1245 (10.33)
Week 6 (May 27-May 31)	706 (59.1)	488 (40.9)	1194 (9.90)
Week 7 (June 3-June 7)	799(62.0)	489 (38.0)	1288 (10.68)
Week 8 (June 10-June 14)	788(61.7)	490 (38.3)	1278 (10.60)
Week 9 (June 15- June 21)	802 (62.6)	479 (37.4)	1281 (10.63)
Week 10 (June 22- June 28)	798 (62.0)	490 (38.0)	1288 (10.68)
**Total**	**7139 (59.2)**	**4917 (40.8)**	**12056 (100)**

A total of 6,383 (52.9%) males and 5,673 (47.1%) females were observed in the study. Among the observed, majority 9,832 (81.6%) were in the estimated age group of 18-50 years years(Table 2). The use of surgical mask predominated, 51% relative to cloth mask, 41%, N95, 4%, and scarf, 4%.

Table 2: Demographic profile of participants by sub-city (April 20– June 14, 2020, Addis Ababa)

Variable	Total n (%) (n=12056)
Sex	
Male	6383 (52.9)
Female	5673 (47.1)
Age*	
<18 years	551 (4.6)
18-50 years	9832 (81.6)
>50 years	1673 (13.9)

*Age categories were made broad purposely in order to identify the closest age using observation without asking an individual

The results showed that proper hand hygiene in the community slowly increased from 24.2% in week one to 32.5% in week three, and then slowly declined to 23.7% in week 10. Similarly, proper physical distancing in the community increased from 34.4% in week one to 42.6 % in week four, and then slowly declined to 35.2 % in week eight. On the contrary, proper respiratory hygiene in the community continuously increased from 14% in week one to 77% in week 10ten. Overall, we have found the within observations statistical differences for the three practices (p<0.05), and within subject differences by sex, age and day of observation (P<0.05).

Drivers' respiratory hygiene continuously increased from 13.8 % in week one to 84.6 % in week 10, and from no respiratory hygiene practice among assistant drivers in week one to 70.5% in week six then slowly declined to 54.1% in week 10 (Table 3). The results also showed that adherence to the government mandated less than or equal to 50% occupancy in commercial vehicles declined from 88.8% in week one to 68.8 % in week 10.

Table 3: Weekly pattern of community practice of non-pharmaceutical interventions (Addis Ababa, 2020)

NPI	NPI Practices	Week 1 (Apr20-26)	Week 2 (Apr27-May3)	Week 3 (May 4-10)	Week 4 (May 11- 17)	Week 5 (May20- 24)	Week 6 (May 27-31)	Week 7 (June 3-7)	Week 8 (June10-14)	Week 9 (June15-21)	Week 10 June 22-28
Hand hygiene	**Proper hand hygiene**	**178 (24.2)**	**328 (33.1)**	**350 (32.5)**	**323 (32.0)**	**305 (28.8)**	**26 1 (25.9)**	**288 (26.1)**	**290 (26.8)**	**281 (28.5)**	**254 (23.7)**
	Improper hand hygiene	88 (11.9)	93 (9.4)	101 (9.4)	76 (7.5)	78 (7.4)	80 (7.9)	121 (10.9)	105 (9.7)	123 (12.4)	103 (9.6)
	No hand hygiene	469 (63.8)	571 (57.6)	625 (58.1)	610 (60.4)	674 (63.8	667 (66.2)	692 (62.8)	684 (63.4)	582 (59.1)	715 (66.7)
	**Total**	**735**	**992**	**1076**	**1009**	**1057**	**1008**	**1101**	**1079**	**986**	**1072**
Physical distance	**Proper physical distance**	**294 (34.4)**	**462 (39.7)**	**491 (38.8)**	**511 (42.6)**	**46 7 (37.5)**	**444 (37.2)**	**479 (37.2)**	**496 (38.8)**	**503 (39.3)**	**454 (35.2)**
	Improper physical distance	408 (47.8)	531 (45.6)	593 (46.9)	551 (46.0)	655 (52.6)	643 (53.9)	699 (54.3)	662 (51.8)	711 (55.5)	710(55.1)
	No physical distance	152 (17.8)	172 (14.8)	180 (14.2)	137 (11.4)	123 (9.9)	107 (9.0)	110 (8.5)	120 (9.4)	67 (5.2)	124(9.6)
	**Total**	**854**	**1165**	**1264**	**1199**	**1245**	**1194**	**1288**	**1278**	**1281**	**1288**
Respiratory hygiene	**Proper respiratory hygiene**	**205 (24.0)**	**301 (25.8)**	**405 (32.0)**	**575 (48.0)**	**746 (59.9)**	**845 (70.8)**	**985 (76.5)**	**980 (76.7)**	**999 (78.0)**	**995 (77.3)**
	Improper respiratory hygiene	79 (9.3)	174 (14.9)	213 (16.9)	271 (22.6)	151 (12.1)	153 (12.8)	198 (15.4)	214 (16.7)	208 (16.2)	201 (15.6)
	No respiratory hygiene	570 (66.7)	690 (59.2)	646 (51.1)	353 (29.4)	348 (28.0)	196 (16.4)	105 (8.2)	84 (6.6)	74 (5.8)	92 (7.1)
	**Total**	**854**	**1165**	**1264**	**1199**	**1245**	**1194**	**1288**	**1278**	**1281**	**1288**
Drivers' respiratory hygiene	**Proper respiratory hygiene**	**11 (13.8)**	**34 (23.0)**	**91 (55.2)**	**133 (78.7)**	**159 (84.6)**	**170 (89.5)**	**167(84.8)**	**165(82.9)**	**156(82.5)**	**168 (84.6)**
	Improper respiratory hygiene	1 (1.3)	11 (7.4)	15 (9.1)	11 (6.5)	23 (12.2)	17 (8.9)	29 (14.7)	30 (15.1)	30 (15.9)	27 (13.6)
	No respiratory hygiene	68 (85.0)	103 (69.6)	59 (35.8)	26 (15.3)	6 (3.2)	3 (1.6)	1 (0.5)	4 (2.0)	3 (1.6)	4 (2.0)
	**Total**	**80**	**148**	**165**	**170**	**188**	**190**	**197**	**199**	**189**	**199**
Driver assistants' respiratory hygiene	**Proper respiratory hygiene**	**0%**	**29 (19.6)**	**60 (37.3)**	**87 (52.1)**	**119 (62.6)**	**141 (70.5)**	**135 (69.9)**	**118 (60.5)**	**111 (59.0)**	**106 (54.1)**
	Improper respiratory hygiene	3(4.0)	19(12.8)	29 (18.0)	44 (26.3)	61 (32.1)	48 (24.0)	57 (29.5)	72 (36.9)	73 (38.8)	80 (40.8)
	No respiratory hygiene	72 (96.0)	100 (67.6)	72 (44.7)	36 (21.6)	10 (5.3)	11 (5.5)	1 (0.5)	5 (2.6)	4 (2.1)	10 (5.1)
	**Total**	**75**	**148**	**161**	**167**	**190**	**200**	**193**	**195**	**188**	**196**
Vehicle occupant capacity	**Less than or equal to 50% capacity**	**71 (88.8)**	**122 (83.0)**	**114 (69.1)**	**110 (65.1)**	**128 (68.1)**	**128 (67.4)**	**136(69.0)**	**140(70.4)**	**132(69.8)**	**137(68.8)**
	Greater than 50% capacity	9 (11.3)	25 (17.0)	51 (30.9)	59 (34.9)	60 (31.9)	62 (32.6)	61 (31.0)	59 (29.6)	57 (30.2)	62 (31.2)
	**Total**	**80**	**147**	**165**	**169**	**188**	**190**	**197**	**199**	**189**	**199**

The results also indicated sharp rise in the practice of respiratory hygiene, while those of hand hygiene and physical distancing did not change or declined during the last week of observation (Figure 2).

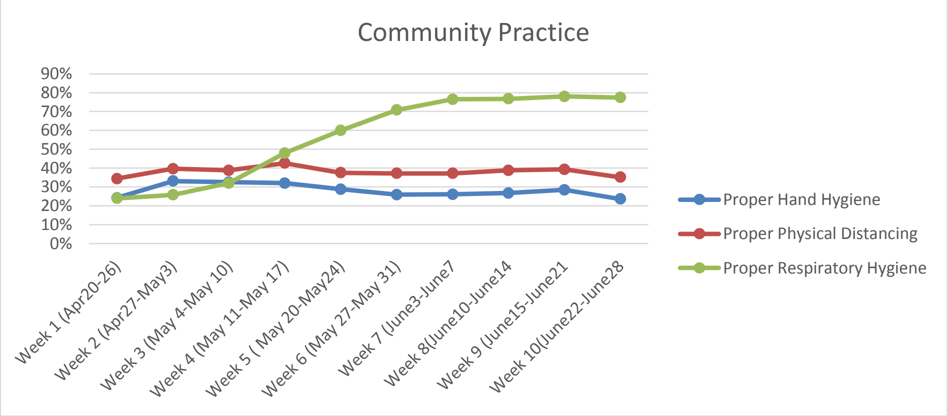

Figure 2: The overall trend of the proper community practices for the three universal precautions

The practice of respiratory hygiene showed fast increase at all service provision sites observed, and the highest change was at banks (from 30% in week one to 90% in week 10) (Figure 3).

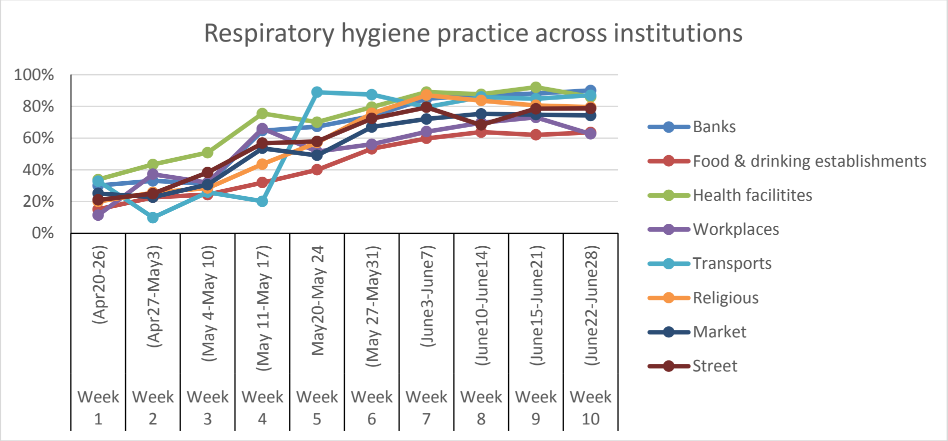

Figure 3: The trend of the proper respiratory hygiene practices at different institution sites

The three combined practices of hand and respiratory hygiene as well as physical distancing increased at a slow pace from 4.7% in week one to 10.8% in week four, and then declined to 8.1% in week 10. ~~On the other hand, two combined practices of proper distancing and respiratory hygiene showed continuous improvement from 11.4% in week one to 32.2% in week nine. Similarly, two combined practices of proper hand and respiratory hygiene increased continuously from 6.8% in week one to 20.2% in week nine, while two combined practices of hand hygiene and phsyical distancing barely showed any change between week one (10.7%) and week 10 (11.5%) (Table 4).~~

Table 4: Combined differents community practices

Risk category	Risk classification	Week 1 (Apr 20-26)	Week 2 (Apr 27-May 3)	Week 3 (May 4-10)	Week 4 (May 11-17)	Week 5 May 20-24	Week 6 May 27-31	Week 7 June 3-7	Week 8 June10-14	Week 9 June15-21	Week 10 June22-28	Total
**Proper hand hygiene & distancing & respiratory**	Low risk	40 (4.7)	79(6.8)	96 (7.6)	130 (10.8)	99 (8.0)	95 (8.0)	98 (7.6)	123 (9.6)	113 (8.9)	100 (7.8)	973 (8.1)
**Proper distance & respiratory**	Low risk	97 (11.4)	150(12.9)	206 (16.3)	264 (22.0)	311 (25.0)	334 (28.0)	396 (30.7)	405 (31.7)	413 (32.2)	383 (29.7)	2959 (24.5)
**Proper hand hygiene & distancing**	Medium risk	91 (10.7)	172(14.8)	174 (13.8)	180 (15.0)	149 (12.0)	118 (9.9)	116 (9.0)	140 (11.0)	130 (10.3)	110 (8.6)	1380 (11.5)
**Proper hand and resp hygiene**	Medium risk	58 (6.8)	127(10.9)	163 (12.9)	205 (17.1)	183 (14.7)	193 (16.2)	237 (18.4)	250 (19.6)	240 (20.2)	227 (17.8)	1883 (15.8)
**Only mask**	High risk	73 (8.5)	68(5.8)	90 (7.1)	165 (13.8)	276 (22.2)	328 (27.5)	348 (27.0)	375 (29.3)	290 (24.1)	381 (29.8)	2394 (20.0)
**Only physical distancing**	High risk	130 (15.2)	183(15.7)	181 (14.3)	176 (14.7)	87 (7.0)	68 (5.7)	50 (3.9)	54 (4.2)	59 (4.6)	47 (3.7)	1035 (8.6)
**Only hand hygiene**	High risk	69 (8.1)	108(9.3)	109 (8.6)	68(5.7)	72(5.8)	45(3.8)	33(2.6)	23(1.8)	24(1.9)	17(1.3)	568 (4.7)
**No hand & respiratory hygiene & no distancing**	High risk	305 (35.7)	350 (30.0)	375 (29.7)	248 (20.7)	211 (16.9)	161 (13.5)	162 (12.6)	141 (11.0)	124 (9.8)	169 (13.1)	2246 (18.7)

*Risk classification was made based on the level of three practices at individual level. It is a relative term: an individual practicing the three is in low risk, while none of only doing hand hygiene and physical distance in high-risk group. An individual with two combination is in the medium risk.

Discussion

~~As SARS-CoV-2 (COVID-19) in the presence of limited vaccine or effective treatment,~~ N~~n~~on-pharmaceutical interventions are the primary ~~major~~ ~~strategic efforts~~preventions ~~to prevent~~ to restrict the ~~introduction of new infections~~spread of Covid-19 on the background of limited access to vaccines. ~~This study was based on community observation data on non-pharmaceutical interventions recommended by World Health Organization (WHO) (14).~~ Overall, our results demonstrated the use of mask was able to be stable at 80%~~satisfactory~~, while hand hygiene and physical distancing had low compliance rate ~~were not to the optimum level~~~~as good as mask using~~. The world Health organization greatly acknowledges the combined practice of non-pharmaceutical interventions as an effective tool to combat the infection (14). ~~Non-pharmaceutical interventions have been implemented to reduce transmission of COVID-19 in many countries. An earlier modelling in China has shown t~~ Evidence exist that ~~T~~the non-pharmaceutical interventions ~~to b~~were~~e~~ effective in containing the outbreak, and that the number of cases were predicted to have been 67-fold higher at the time without these interventions (15). The practice of these preventions are effective with optimum coverage if not universal in a community as has been strongly proposed in China for a high risk population (16)(Reference). However, adherence to these preventive practices has been seen to be declining in ~~many~~ countries in recent times (Reference). For instance, the Nigerian Medical Association has ~~been reported~~ strongly condemned the limited use ~~to decry the flagrant disregard for~~of the non-pharmaceutical prevention protocols by citizens (17). The low level of mask using was mainly attributed to risky social behaviors, perceived risk of transmission, gender and age differences (18). This study also showed the variation of mask using over time across many countries: some having gradually increasing, others showing a declining trend. The access of community to affordable type of in our study should have provided a sense of protection compared hand hygiene and physical distancing. The use of cloth masks that is easily accessible, cheap and existing in many different options has provided degree of protect next to surgical masks.

The present study has shown major improvement in the practice of respiratory hygiene compared to hand hygiene and physical distance during the period of observation. The improvement started to show significant change since week five that matched with the

Commented [AK1]: Order of content
Clarity
Strengths
Limitations

Formatted: Highlight

Formatted: Highlight

Formatted: Highlight

time when security forces started to enforce ~~the use of mask~~primary preventions ~~that were as mandated by~~in response to ~~-~~the state of emergency ~~to respond~~in response to the ~~pandemic~~ epidemic crisis in Ethiopia. Although the State emergency was declared on 20 April 2020, its impact came later, just after few weeks that have been used for massive promotional provisions targeting the public at large and ~~ground preparations for the provisions of mask and hand hygiene facilities by~~ public service rendering institutions in particular. The State Emergency in Ethiopia at the time of the study was characterized to have partial lockdown of public places including closing of educational facilities and work places coupled with strict use mask at public places, such as transport and public service places which resulted in rapid increasing during our monitoring period. ~~-~~The perception of self-protections by using mask might have contributed towards the increasing practice, as has been observed in other studies (refernce). To the contrary, a small portion of the public did not use mask that might be linked to the negative attitudes towards masks ineffectiveness to reduce the transmission (19)

According the provisions of this State of Emergency, public mobility was not restricted for seeking food, transport, health care, and marketing services. There are reports from other ~~studies~~ countries that NPI interventions ~~intensive interventions~~ during ~~with~~ lockdown periods ~~as a way of being~~are effective tools ~~would need to be in place for the preventive measures to be effective since adherence remains as a way~~ to interrupt covid-19 transmission (20, 21).

XXX The State Emergency ~~had a provision that~~ mandated private and government service providers to provide hand washing facilities with detergents for their clients seeking services. Hand washing facilities are usually placed at the gate or entrance of the institution to encourage hand hygiene before accessing the service (Figure XX). Despite the progressed public enforcement. ~~T~~the practice of proper hand hygiene did not show much change over the 10 weeks of observation, and could no went beyond 30%. Two reasons were indicated from ~~the qualitative~~our observation. Firstly, the media company and involvement of the enforcing volunteers, had massive ~~interventions~~ promotions in the

Formatted: Highlight

Formatted: Highlight

Commented [AK2]: Include literature

Commented [AK3]: Include refernces

Formatted: Highlight

Formatted: Highlight

Formatted: Highlight

Formatted: Highlight

Formatted: Highlight

Formatted: Highlight

Formatted: Highlight

Formatted: Highlight

Formatted: Highlight

Formatted: Highlight

Commented [AK4]: First diuscuss mask using. Then others

Formatted: Highlight

Formatted: Highlight

Commented [AK5]: Figure needed

Formatted: Highlight

Formatted: Highlight

Formatted: Highlight

first month during ~~the~~ our data collection period ~~especially~~ around churches, transport stations, and other public places. This has contributed a change in hand washing practice in that month but not much beyond that month. On the other hand, the unavailability of water and soap ~~for washing hand~~ could not be sustained in many facilities, except few public services providing institution such as banks. ~~Our qualitative observation has documented this practice~~. Generally, the provision of soap was challenged first, that was followed by the shortage and total disappearance of water in many ~~fronts of~~ public service providers. We believe, these happened at the time of weak enforcement when security and other partners discontinued ~~lifted off their~~ initiated interventions~~effort~~. There was a shift from washing hand to using hand sanitizers by some members of the population especially the youth. ~~and o~~Our result had limited coverage of the ~~did not include~~ youth, ~~that~~ thus we might have ~~may~~ underestimated the proportion of hand washing in a community. ~~The youth usually did not used to visit the facilities we have opted, thus unlikely to be covered by our study.~~

The maintenance of physical distancing at a minimum of 1 m between any two individuals had similar trend with hand washing practice, although the former had overall performance of below 40% relative to the 30% of the later practice. Physical distancing was ~~a lot~~ challenged context wise ~~in many spheres~~ of social services, predominantly observed in open markets, ~~at times of~~ walking and crossing on a street, ~~when crossing streets~~, and seeking public transport sectors. ~~The context of the provisions of public services is likely to affect the performance of physical distancing~~. Individuals in an ~~Ethiopian~~ open market (Figure XX) traditionally tends to cluster around a seller in order to make transactions. ~~Limited and unpredicted~~Seeking public transport services at peak hours make service seekers to get closer around the gate of transport vehicles (Figure XX). ~~There is a public health concern that such low level of physical distancing may enhance transmission~~. The duration of exposure and physical distancing ~~distance of the exposure to SARS infection~~ have been shown an important predictor of the transmission of Covid-19(22). A ~~previous~~ systematic review of 172 studies reported that physical distancing of one meter was associated with large reduction in the number new infections in different countries (23). Taking the economic status of Ethiopia, lack of change in the physical distancing at the public level in this study was a missed opportunity to avert the

Commented [AK6]: ADD photo

Commented [AK7]: Add photo

occurrence of new cases in the city given. Hand hygiene and physical distancing are an effective low-cost intervention that could reducing the number of new cases, hence the severity of cases through reducing viral inoculation (24, 25). We believe limited adherence in hand hygiene and physical distancing are missed opportunities to avere the occurrence of new infections in a community.

Ethiopia possesses traditions that enhances the community to get together, such as burial events, traditional religious festivals, attending churches and traditional open markets. State emergency was not universal in the restriction of mass mobility during its active implementation phase, which might be a factor for the spread of the infection. Clients are advised to perform the three practices strictly during the initial wave of the epidemic, the restriction became. However, week after lifting of the state emergency. There were incidences when the socio-cultural events ignores physical distance because of the crowding of population attending that event such us religious holidays.   The problem of not practicing physical distancing was observed during the Ethiopian religious festivals. This was wild in our traditional open market settings. Inconsistent to this finding, a study from China reported that almost all respondents avoided going to public places and reduced celebrating spring festivals due to COVID-19 epidemic in the country (26). The possible reasons for the difference in the adherence might be associated with the socio-cultural contexts of the study population in a given country and the degree of enforcing preventive practices.

The extent and trend of mask utilization had visible difference compared to physical distancing and ~~other practices~~hand hygiene. Mask utilization has shown an increasing trend until it stabilizes at about 80% towards the end of our study time. Many factors operated. The continued security enforcement, mass media mobilization, therestrictions of public services ~~banning of visitors and passengers to get a service~~ without a mask, and the provisions of regular information about covid-19 using national TVs and radios were the main ~~ones~~contributing factors. The multiple sources of information and public enforcement ~~is~~ were believed to enhance the attitude of service seekers ~~towards the use of mask~~ which greatly modified their behavior towards the use of mask. We cannot deny at all that the access to multiple types of mask, such as surgical, N95, and cloth, their local production at affordable prices has critically sustained ~~greatly changed~~ the trend of mask utilization. The local production of face masks in low-middle income countries has been indicated as a strategy to contain the epidemic (27). Systematic reviews have shown the frequent practice of hand hygiene, the use of physical distancing of 1 m or more and mask utilization have a potential benefit of lowering the covid-19 transmission (23, 28). The time of the observation, before or after the State emergency, and the age of study subjects could make a difference in the proportion of mask using. There is a preference of using mask among young population in Poland, making the figure at 60%, a study undertaken before the government's enforcement (29), which is relatively lower than ours. The study in Poland was among young population which is not consistent with ours. Our study was undertaken after the State Emergency, which has brought multiple interventions to impact the behaviors of individuals in using mask. Overall, the universal use of mask in a public has been shown as an effective tool to reduce the transmission of covid-19 in previous studies ~~targeting~~ (30, 31).

We disaggregated the mask using by type of facilities in order to get insights about the factors affecting mask using. The trend of respiratory hygiene practice visibly increased among service providers involving ~~in the entrances to the~~ health care facilities, ~~and~~ banks and transport services compared with other facilities included in this study. There were assigned controllers and guards checking for the use of face-mask and maintenance of physical distancing and hand hygiene, who did not allow individuals to enter without these practices~~using i~~t. ~~The periodic control by the security forces on the streets and around churches and by the guards of supermarkets was also observed while no control was there in the studied work places, open markets, and around food and drink establishments. The problem of respiratory hygiene in the food and drink establishments requires close attention because people used to eat in groups and physical distancing~~

Formatted: Left, Space After: 10 pt, Line spacing: Multiple 1.15 li, Adjust space between Latin and Asian text, Adjust space between Asian text and numbers

was almost not practically applied. Onsite dinning with indoor and outdoor seats was identified as risky situation by CDC(10).

The relatively improved practices at bank and health facilities sites is may be related to the fact that these institutions have committed the resources to provide hand washing facilities with detergents and that their clients would be relatively higher level of awareness and favorable attitude towards the preventive practices. In addition, the presence of security guards at the entrance/ gate of a bank has a lot enhanced compliance. As there are variabilities in people's beliefs about the effectiveness as well as the rates of engagement in the use of non-pharmaceutical interventions, public health efforts should focus on increasing perceived severity and threats of the COVID-19, while promoting the preventive measures as effective tools (32, 33) and enforcing the use of them as necessary.

To the contrary the adherence in mask using and physical distance in open markets and food establishments will continue to exist as a challenge. Food and drink establishments require close attention because people used to eat in groups and physical distancing was almost not practically applied. Onsite dinning with indoor and outdoor seats was identified as risky situation by CDC(10).

The comprehensive use of the three practice at each individual level was surprisingly very low, just below 10%. Any two combination was also low, that did not have a public health relevance. Such limited adherence The risk characterization by the level of combination is a concern given the presence of the covid-19 spread in a population. The very limited practice in a combined practice is an indication of a challenge andand the presence of a threat to sustain the spread of transmission. WHO greatly advocates the complete and regular use of these practices at every individual level when getting outside home (34). With such low level of practice coupled with limited distance of exposure, there could be an increased likely of covid-19 transmission in a population any time, hence exploding the epidemic. The trend of viral related pneumonia had demonstrated a declining trend that is asserted with the likely provisions of wearing mask, hand hygiene and social distancing in a Taiwan epidemiological study (35). WHO informs that the only use of

masks in the context of COVID-19 suggested the risk of false sense of security, which could lead to potential less compliance of other behaviors because of the risk of self-contamination during the manipulation of the mask.

This study has strengths in view of observing individuals randomly and anonymously at times of service provisions of peak hours in ~~major~~ key social providing services. The provision of weekly ~~regular~~ information to the Ministry of Health was an important input to enhance informed decisions to the public. ~~However, observing individuals throughout the day in each facility was not possible due to limited resources.~~ We assumed observing individuals at the time of possible transmission in the presence of increased mobility and crowding provides better insights of the possibility of the transmission potential. ~~Another area of limitation is in ascertaining the appropriate use of masks in reference to checking fitness. The context of this study could not allow to optimize complete information in the general population. The benefit of this type of observation was to inform policy using research evidences~~XXX.

Commented [AK8]: Revise

Formatted: Highlight

Commented [AK9]: Describe limitations of the sstudy

Formatted: Highlight

Conclusions and Recommendations

The overall community prevention practices of COVID-19 pandemic were very low in Addis Ababa, Ethiopia. Despite the relatively low adherence of the community on proper hand washing and proper physical distancing, the success of proper respiratory hygiene was encouraging which requires inclusively the proper practice of hand hygiene and physical distancing. We call to strengthen social mobilization using available medias and consistent enforcement mechanisms.

Funding: The data collection was funded by the School of Public Health of Addis Ababa University. The Authors have contributed professionally in view of supporting the Ministry of Health in enhancing proactive informed decisions.

Acknowledgements: The authors are grateful to the College of Health Sciences and the School of Public Health of Addis Ababa University for allowing this study to happen.

Competing interest: I do not have any financial competing interests and any conflicts in this publication with any other person or institution.

Author's Contributions

DH tabled a research idea on the need of generating the status of community practice. AK was involved in the writing of the proposal and drafting the manuscript. AK and SW were involved in the analysis of data. MT was involved in handling the field qualitative information. WA was involved in the design of data template in ODK. YT and TA were involved in the design of observation tools. All were involved in editing the proposal and manuscript.

References

1. Chen X, Yu B. First two months of the 2019 Coronavirus Disease (COVID-19) epidemic in China: real-time surveillance and evaluation with a second derivative model. . Glob health res policy. 2020;5(7(2020). https://doi.org/10.1186/s41256-020-00137-4).
2. Beattie RM, Ashton JJ, Penman ID. COVID-19 and the gastrointestinal tract: emerging clinical data. Frontline Gastroenterology. 2020:flgastro-2020-101507.
3. Liu J, Liao X, Qia nS, Yuan J, Wang F, Liu Y, et al. Community transmission of severe acute respiratory syndrome coronavirus 2, Shenzhen, China, 2020. Emerg Infect Dis. 2020;26(6).
4. Guoqing Q, Naibin Y, Ada HYM, Liping W, Guoxiang L, Xueqin C, et al. COVID-19 Transmission Within a Family Cluster by Presymptomatic Carriers in China Clinical Infectious Diseases. 2020;71(15):861-.
5. Yu X, Yang R. COVID-19 transmission through asymptomatic carriers is a challenge to containment. Influenza Other Respir Viruses. 2020;14(4):474–5.
6. Dudley JP, Lee NT. Disparities in Age-specific Morbidity and Mortality From SARS-CoV-2 in China and the Republic of Korea. Clin Infect Dis. 2020;71(15):863-5. Epub 2020/04/02.
7. Palaiodimos L, Kokkinidis DG, Li W, Karamanis D, Ognibene J, Arora S, et al. Severe obesity, increasing age and male sex are independently associated with worse in-hospital outcomes, and higher in-hospital mortality, in a cohort of patients with COVID-19 in the Bronx, New York. Metabolism. 2020;108:154262. Epub 2020/05/19.
8. World Health Organization. Infection prevention and control of epidemic- and pandemic-prone acute respiratory infections in health care 2014 [cited 2020 April 2]; Available from: https://apps.who.int/iris/bitstream/handle/10665/112656/9789241507134_eng.pdf?sequence=1.

9. European Center for Diseases Prevention and Control. Infection prevention and control and preparedness for COVID-19 in healthcare settings. [cited 2020 April]; Available from: https://www.ecdc.europa.eu/sites/default/files/documents/Infection-prevention-control-for-the-care-of-patients-with-2019-nCoV-healthcare-settings_update-31-March-2020.pdf.
10. Centers for Diseases Control and Prevention. Implementation of Mitigation Strategies for Communities with Local COVID-19 Transmission. [cited 2020 April]; Available from: https://www.cdc.gov/coronavirus/2019-ncov/downloads/community-mitigation-strategy.pdf.
11. World Health Organization. Corona virus disease (COVID-19) advice for the public.https://www.who.int/emergencies/diseases/novel-coronavirus-2019/advice-for-public. [cited 2020 April]; Available from: https://www.who.int/emergencies/diseases/novel-coronavirus-2019/advice-for-public.
12. Worldometers. Ethiopia: Corona Virus Cases. https://www.worldometers.info/coronavirus/country/ethiopia/. 2020.
13. Pei S, Kandula S, Shaman J. Differential Effects of Intervention Timing on COVID-19 Spread in the United States. medRxiv. 2020:2020.05.15.20103655.
14. World Health Organization. Coronovirus. https://www.who.int/health-topics/coronavirus#tab=tab_2. 2020.
15. Shengjie L, Nick WR, Liangcai Z, Olivia Prosper, Wei L, Jessica RF, Amy W, et al. Effect of non-pharmaceutical interventions to contain COVID-19 in China. Nature. 2020;585:410-3.
16. Joseph TFL, Xilin Y, Tsui HY, Ellie P. SARS related preventive and risk behaviours practised by Hong Kong-mainland China cross border travellers during the outbreak of the SARS epidemic in Hong Kong. J Epidemiol Community Health. 2004;58:988-96.
17. Alexander D. NMA Condemns Non-Adherence To COVID-19 Prevention Protocols. 2020 [cited 2021 January]; Available from: https://consumerconnectng.com/8186.
18. Badillo-Goicoechea E, Chang T-HK, Esther, LaRocca S, Morris K, Deng X, Chiu S, et al. Global Trends and Predictors of Face Mask Usage During the COVID-19 Pandemic. arXiv. 2021(https://arxiv.org/ftp/arxiv/papers/2012/2012.11678.pdf).
19. Taylor S, Asmundson G. Negative attitudes about facemasks during the COVID-19 pandemic: The dual importance of perceived ineffectiveness and psychological reactance. PLoS ONE 16(2. PLoS One. 2021;16(2):e0246317. https://doi.org/10.1371/journal.pone.
20. Yacong B, Cui G, Changqing L, Samuel YSW, Alexis KHL, Xiang QL. Effectiveness of non-pharmaceutical interventions on COVID-19 transmission in 190 countries from 23 January to 13 April 2020. International Journal of Infectious diseases. 2021;102:247-51. ttps://doi.org/10.1016/j.ijid.2020.10.066.
21. Stutt R, Retkute R, Bradley M, Gilligan C, Colvin J. A modelling framework to assess the likely effectiveness of facemasks in combination with 'lock-down' in managing the COVID-19 pandemic. Proceedings of the Royal Scociety. A 476: 20200376. http://dx.doi.org/10.1098/rspa.2020.0376. 2020.
22. Rea E, Laflèche J, Stalker S, uarda BK, Shapiro H, Johnson I, et al. Duration and distance of exposure are important predictors of transmission among community contacts of Ontario SARS cases. https://doi.org/10.1017/S0950268806007771. Epidemiology and infection. 2007;135(6):914–21.
23. Chu DK, Akl EA, Duda S, Solo K, Yaacoub S, Schünemann HJ. Physical distancing, face masks, and eye protection to prevent person-to-person transmission of SARS-CoV-2 and COVID-19: a systematic review and meta-analysis. Lancet. 2020;395(10242):1973-87.
24. Dalton CB, Corbett SJ, Katelaris AL. COVID-19: implementing sustainable low cost physical distancing and enhanced hygiene. Medical journal of Australia. 2020;212(10):443–6.e1. https://doi.org/10.5694/mja2.50602.
25. O M, F N, O R. [Epidemiology and control of COVID-19] (Abstract). Dtsch Med Wochenschr. 2020;145(10):670-4. doi 10.1055/a-162-987.

26. Global Times. Beijing districts, companies to enter into a state of emergency: official. 2020 [cited 2020 March]; Available from: https://www.globaltimes.cn/content/1211098.shtml.
27. Missoni E, Armocida B, Formenti B. Face Masks for All and All for Face Masks in the COVID-19 Pandemic: Community Level Production to Face the Global Shortage and Shorten the Epidemic. Disaster medicine and public health preparedness. 2020:1-5. https://doi.org/10.1017/dmp.2020.207.
28. Jefferson T, Del Mar CB, Dooley L, Ferroni E, Al-Ansary LA, Bawazeer GA, et al. Physical interventions to interrupt or reduce the spread of respiratory viruses. The Cochrane database of systematic reviews, 2011(7), CD006207. . Cochrane Database Syst Rev. 2011;7(CD006207). https://doi.org/10.1002/14651858.CD006207).
29. Matusiak Ł, Szepietowska M, Krajewski PK, Białynicki-Birula R, zepietowski JC. The use of face masks during the COVID-19 pandemic in Poland: A survey study of 2315 young adults. Dermatologic therapy. 2020(e13909):Advanced online publication. https://doi.org/10.1111/dth.13909.
30. Jefferson T, Del Mar C, Dooley L, Ferroni E, Al-Ansary LA, Bahamdan SA, et al. Physical interventions to interrupt or reduce the spread of respiratory viruses: systematic review. Br Med J. 2009;339(b3675).
31. Zhang L, Peng Z, Ou J, Zeng G, Fontaine RE, Liu M, et al. Protection by face masks against influenza A(H1N1)pdm09 virus on trans-Pacific passenger aircraft, 2009. Emerg Infect Dis. 2013;19(9). Epub 2013/08/24.
32. Van den Broucke S. Why health promotion matters to the COVID-19 pandemic, and vice versa. Health Promot International. 2020;35(2):181-6. doi: 10.1093/heapro/daaa042.
33. Shahnazi H, Ahmadi-Livani M, Pahlavanzadeh B, Rajabi A, Shoaib MH, Charkazi A. Shahnazi, H., Ahmadi-Livani, M., Pahlavanzadeh, B. et al. Assessing preventive health behaviors from COVID-19: a cross sectional study with health belief model in Golestan Province, Northern of Iran. Infectious Disease Poverty. 2020;0(157):https://doi.org/10.1186/s40249-020-00776-2.
34. World Health Organization. Coronavirus disease (COVID-19) advice for the public. 2021 [cited 2021 January]; Available from: https://www.who.int/emergencies/diseases/novel-coronavirus-2019/advice-for-public.
35. Chiu N, Chi H, Tai Y, Peng C, Tseng C, Chen C, et al. Impact of Wearing Masks, Hand Hygiene, and Social Distancing on Influenza, Enterovirus, and All-Cause Pneumonia During the Coronavirus Pandemic: Retrospective National Epidemiological Surveillance Study. J Med Internet Res. 2020;8:e21257. doi: 10.2196/.

30 June2 021

To: Editor-in-Chief, Plos One

From: Authors of the **Manuscript ID**:

Subject: - "Point by point response to the comments given by the reviewers"

PLOS ONE Decision: Revision required [PONE-D-21-03031]

Title: "**Trends in non-pharmaceutical intervention (NPI) related community practice for the prevention of COVID-19 in Addis Ababa, Ethiopia"**

Manuscript ID: PONE-D-21-03031

Dear Editor-in-Chief
Ref: Email message, 19 March 2021; RE: PONE-D-21-03031
Trends in non-pharmaceutical intervention (NPI) related community practice for the prevention of COVID-19 in Addis Ababa, Ethiopia; "PLOS ONE" plosone@plos.org

We would like to express our appreciation and happiness for inquiring comments for this important work piece. Covid-19 continues to kill people with the focus on older ages. Surprisingly, residents did not yet mind about the primary prevention tools we are trying to disclose.

Overall Reviewer Comments:

Reviewer #1:

Reviewer #2:

Editor in Chief:

Reviewer 1 Specific Comments (MS Section based)

Section	Comments	Response
**General**	1) the lack of statistical analysis with respect to the descriptive data presented and 2) need for language editing and clarity, particularly in the discussion.	**Comments are well taken.**<br><br>Need of using statistical analysis: The study is purely descriptive, based on using proportions, with the focus evaluating the progress of the trend ie looking the within/ weekly variation
**Abstract**	Overall, well written. However, I find the number of observations described in the methods section somewhat confusing and misleading compared to how this is described in the methods section of the paper itself. I would suggest simplifying this description. The statistics presented in the results lack significance analysis (i.e. p-values). I do not think the conclusion in the abstract necessarily match the results described.	• The abstract section was reorganized, simplified, included some statistics.<br><br>• The conclusions are really a necessity as a rule of thumb. This is the final say to a reader **based on data outputs**
**Introduction**	Paragraph 1 – “Pre-asymptomatic cases facilitate most of the transmission” Do you mean pre-SYMPTOMATIC here?	**Thanks for the comment. It was to mean**<br><br>Pre-symptomatic
	Paragraph 2 – I think the first two sentences are not necessary for your background argument.	**Overall, the introduction section was carefully designed paragraph wise:**<br><br>Para 1: a broader perspective<br>Par 2: the statement/ gap<br>Par 3: the local data access<br>Par 4: the concern of the Ministry of Health.<br><br>**If there are objections to this structure, we may omit the two sentences as needed**
	Paragraph 4 – I think the end of the introduction would benefit from	

	a clearer study objective statement as a separate small paragraph	
**Methods**	Paragraph 1 – This paragraph may work better as part of the introduction.	Yes your are right as an option. However, it is better to place it in the methods section for the description of the study area.
	Paragraph 3 – I would clearly state that all age groups (including children) were included in the study population.	**Comment accepted. Edited accordingly**
	Paragraph 4 – It would be helpful to expand on which types of sites (and how many) were included for observation. I would also include more detail about how you observed drivers and public transportation as this was specifically mentioned in the results section.	This has been elaborated on the Monitoring protocol section.<br><br>However, the study sites were included in the study design/ Study area section
	Paragraph 5 – I would include a copy of the two check lists used in the study	Now attached the two tools as apart of Annex.<br><br>The variables of this tool were customized in ODK software. Good to consider as complement file by the editor
	Paragraph 6 – A minimum of 30 minutes of observation time is suggested but was a maximum time period defined? Why was a standard observation time not used?	We, 5 investigators, had a pilot survey for 2 weeks' time to develop and validate the data collection tool. We came across the time to be adequate an average of 30 minutes per facility-site to observe ten individuals.<br><br>We have not looked at the max time, given the relevance of a min of 30 minutes.<br><br>The overall time generally lied, XXX? Ask Shibababw
	Paragraph 7 – With respect to the age variable, I am concerned about the accuracy and reproducibility of estimated age	**Good question**<br><br>We did not worry much on the accuracy and reproducibility of age. The data collector

	categories used in this study. What is the rationale for why these particular age groupings were used? I would also suggest clearly stating how you defined proper and improper respiratory hygiene, hand hygiene and physical distancing for the purpose of this study, rather than referencing WHO. It would also be important to clarify if both soap/water and hand sanitizers were considered adequate hand hygiene as there is some discussion about this later in the paper.	will not ask individuals age, otherwise the purpose of observation is defeated. There will be what is called "Hawthorne's effect'. We used our own experience to label the individual in three categories than many of us can guess the age of an individual:<br>1- < 18 years old<br>2- 18-50 years old<br>3- > 50 years old<br>There could some overlaps, but that is fine as far as individuals practice can well recorded. We used overall our knowledge to judge an individual: someone with lots of grey hair, physically weak, etc could be in most cases > 50 years old. An average person, strong physically is usually 18-50. It is an easy task to identify someone < 18. Overall, the age identification was not an issue during data collection time
	Paragraph 8 – Descriptive statistics do not appear to be used for analysis. This needs to be defined further in this section, the applied to the data and presented in the results/table section.<br>Paragraph 9 – With respect to consent, was the public or any of the places of business used as observation sites made aware of this study?	Thanks for the comment.<br>The analysis is simple, using proportions to describe the trend of the practice. No models are needed.<br>We used some tests to see the within practice difference (difference by time) and between practice differences (by sex and age).<br>This is elaborated in the data analysis section
**RESULTS, FIGURES AND TABLES**	Paragraph 1/Table 1 – Week one appears to be an outlier with respect to number of observations made on a weekday vs weekend compared to the other weeks is there is reason for this? I don't	**Ask SW needs discussion**

	believe it was addressed in the discussion, either.	
	Table 2 is not necessary as this data is easily described in the text of paragraph 2.	**Agreed. Table 2 is Deleted**
	Paragraph 3/Table 3 – No statistical analysis performed therefore unable to know if differences described are significant based on the number of observations made. Why was transportation presented separately in this table? Why not a breakdown by facility type as well (see my comments regarding figure 3)?	The comments are well considered.<br><br>Although the primary focus of this study is on the trend analysis, which does not requite tests of significance, we have now included the within subject (weekly differences) and between subject differences (Page 14). They are significant. This is understood given the presence of low and high proportions of practices.<br><br>Informing data for the driver's and his assistant is important as these two individual could be the source of Covid. The stat emergency in Month/ Year insisted that everyone who is using public transport, should use masks.<br><br>We have observed both the individual and the crew of the vehicle for this purpose. We have attached a photo that shows the relevance of observing the crew and the individual transport users.
	Paragraph 4/Figure 3 – This is interesting data regarding facility type but not particularly clear when presented in this format. I would suggest a breakdown and analysis similar to Table 3	Thanks for the data. It is possible to show the results in a Table format. However, we prefer to show in line graphs, assuming the reader can generally identify easily which facilities are doing well or not. For the sake of clarity, the table is attached as a supplementary file.
	Paragraph 5 – I think it is important to show the compliance data with all three NPI measurements at the same time as it demonstrates how challenging it can be for the	Many thanks for the comments.<br><br>Comments are well taken. Table 4 is deleted, the three combined compliance is described text wise

	general public to adhere to multiple recommendations at the same time. However, I do not feel the discussion of the various other 2 out of 3 NPI combinations is particularly useful.	
	Table 4 – The idea of "risk" categories was not defined in the methods or mentioned in the results section. These categories are arbitrary and I do not feel this particular table/analysis should be included.	Agreed and deleted Table to avoid uncertainties
**Discussion and Conclusion**	The discussion and conclusions requires a significant amount of editing for clarity. There should also be separate paragraphs specifically addressing limitations of the study as well as specific future interventions or research	Extensive editing focusing on clarity was made<br><br>Flow of content of the discussion was modified<br><br>Strengths and limitations were elaborated<br><br>Future recommendations were made
	In paragraph 1, use of face masks was considered satisfactory but what is your definition of this? There is no discussion around types of masks, observed donning/doffing procedures, re-using old/soiled masks. These issues may be beyond the scope of this particular study but should be mentioned in the limitations section.	Satisfactory mask using was meant the the proportion was about 80% which is adequate for breaking the transmission. Edit was made on this sentence.<br><br>Type of mask was indicated in the result section<br><br>Donning and doffing is meant only in health care setting<br><br>Type of mask discussed very briefly
	Paragraph 2 suggests that declining compliance with NPIs in other communities is common over time but it would be helpful to have a discussion around why this	This has been discussed with bthe refernce citation

	occurs.	
	Paragraph 3 addresses how the declaration of a state of emergency and other measures, such as limiting people's movements in the community, are also important interventions. This paragraph requires some editing to clarify this point and what the impact was specifically in Ethiopia.	This has been addressed with a citations
	Paragraph 4 – There are two competing ideas in this paragraph that need to be separated and explored further. The issue of available soap/water stations versus hand sanitizer is important. It is unclear if hand sanitizer was readily available or if it was included an appropriate method of sanitization in the methods of this study. The second issue is around public education campaigns, specifically around hand hygiene. I public education campaigns should be described and explored further with respect to their effect on this study's results and include the other NPIs as well	Hand hygiene was defined in the methods section<br><br>Find the defintions of the 3 practices<br><br>We have described education campaign: National TVs, Public radios, Posters, etc were active at the time of state emergency<br><br>However, we have not collected any data on this assuming our focus was just on the context of recoding baehaviours
	Paragraph 5 – The last sentence of this paragraph is not necessary and I would suggest removing it. Otherwise, this paragraph is very important. Are there any studies that have attempted to look at physical distancing strategies that could be useful in busy, open air market settings from other	"Hand hygiene and physical distancing are an effective low-cost intervention that could reducing the number of new cases, hence the severity of cases through reducing viral inoculation", has been modified. The reader might have understood NPI are useful in reducing the

	countries?	severity of the infection: this is nit true; It reduces the transmission.<br><br>We have included relevant citations
	Paragraph 6 –Were religious and cultural leaders involved in promoting NPIs or encouraging celebrating religious days in smaller groups or at home?	This is a good question.<br><br>Yest they were involved in the promotional activities after getting awareness by the Ministry of Health. They teach to their clients, ie those who visit them; cooperate in providing hand hygiene facilities and detergents to their clients<br><br>This paragraph was reorganized.
	Paragraph 7 – This paragraph seems out of place and many sentences are redundant with ideas addressed in other paragraphs.	Yes this para was reorganized, some deleted

Reviewer 2 Specific Comments (MS Section based)

SN	Comments	Response
1	PLOS ONE does not copyedit accepted manuscripts, so the language in submitted articles must be clear, grammatically correct, and unambiguous.	The language and grammar errors were acknowledged.<br><br>We have worked with someone to edit the language in addition to our effort using online checker
2	Your data in the results section is simply a description of the percentage of different groups of people that you observed adhering to your there non-pharmaceutical interventions. ….. If necessary, you may want to avail of a statistician.	We do not need a statistician for this descriptive study. The purpose of this study is just to generate proportions of good behaviors and not really to do complex statistics. We do not have any intention of testing a hypothesis. We just monitored the status of community practices and related changes. The core statistics is then descriptive with some simple tests of difference
3	It would be helpful if you could describe in more detail the nature of the observations and the observers. Do you believe that there was an element of observer bias over time, and that people changed their behavior because they knew they were being watched in these different settings? Clearly, it appears that adherence to masking increased to a high degree in the context of security force related enforcement. However, why was similar enforcement not seen with physical distancing? (This may have been due to the challenges of enforcing distancing in large crowded areas). It also seems fairly clear that handwashing rates did not increase due, in large part, to a lack of available soap and water as you mention. However, you mention that hand sanitizer use, which is more common amongst youth, was not included in your results. Why is this the case?	This review question has many components<br><br>1. Describing the nature of observation:<br><br>Very briefly addressed in the methods section. Supplemental file is attached to the Journal for details.<br><br>We keep the observation is anonymous ie without any consenting for which ethical approval was obtained.<br><br>The observation over time may bias the information: this is true, but used the following to redice observer's bias<br>1. Observation in one site for 10 individua observation is short (8-30 minutes on the average) depending on the flow of whom to be observed.<br><br>Secondly: the data collector is advised to act as a service recipient or a client like his study subjects.<br><br>Thirdly, he records data on a mobile using ODK which is better than paper work. The

		recording takes very quickly place as if he is dialing….<br><br>2. Mask using significantly increased as a result of security enforcement. This is true. We had a follow up observation that this was not the case on the background of weak enforcement.<br>3. Physical distancing was not as strong as mask using. This is because of unenviable, uncontrolled crowding.<br>4. Hand hygiene did not progress well: yes this is due to resource limitation: water and detergent limitations<br><br>All of the above points were addressed in the discussion section
5	With respect to your age demographic info (i.e < 18 yo, 18-50 yo and >50 yo), you mention that it was easy to categorize people into these groups based on appearance alone. Although it may be easy to tell a 35-year-old apart from a 75 year old, it may have been difficult to tell a 17 year old apart from a 19 year old. Ultimately, these discrete categories seem a bit arbitrary, given that they are "guesstimates" at best.	This is a nice concern.<br><br>Look, if we intend ask the age, we will have a self-defeating in terms of getting valid observational, unbiased ones. Guessing the best way to do it. There could be overlaps in age categories, which will not affect the focus of this study: observing individuals in the context. We will consider, the age categorization, however, as a limitation

PONE-D-21-03031
Trends in non-pharmaceutical intervention (NPI) related community practice for the prevention of COVID-19 in Addis Ababa, Ethiopia
PLOS ONE

30 June 2021

Response to Journal Academic Editor, John Conly, MD, PLOS ONE

SN	Comments	Response
1	Please address all the reviewer comments with the exception of Reviewer 2's comments about sample size given this is a descriptive epidemiologic study.	Response to each comment of the reviewer was mad in the text using a word track in word Review tool; It is also addressed in 3 files:<br>1. Response to Academic Editor<br>2. Response to Reviwer1<br>3. Response to Revier 2
2	You can look at significance of differences between groups and provide some mean and median values where appropriate	We have indicated basic tests in the data analysis section and Results, Just basic ones in terms of overtime differences and within subject differences
3	Both reviewers commented about the details surrounding the NPIs and would agree more details are required other than a general WHO reference and the respiratory hygiene which is presumably referring to masks wearing needs to be defined more precisely.	This is an important concern. WHO has just given the definitions. We have customized that definition for a research purpose. The practice was categorized into Proper, not proper and not using.<br>Just an example:<br>Proper mask using: the mouth and nose is covered completely; Improper: there is a mask on the face, but either one of them or both were not covered. A mask under a chin is also improper;

		Not used-if the mask is not at all seen in the face; if the mask is just handled by hands when it should be used.<br>All of this is indicated in the tool and a protocol that we have developed as SOP for field application.<br>The two docs are attached as a supplementary doc
4	The references to large amounts of transmission from asymptomatic cases needs updating (see Koh WC, Naing L, Chaw L, Rosledzana MA, Alikhan MF, Jamaludin SA, Amin F, Omar A, Shazli A, Griffith M, Pastore R, Wong J.<br><br>What do we know about SARS-CoV-2 transmission? A systematic review and meta-analysis of the secondary attack rate and associated risk factors. PLoS One. 2020 Oct 8;15(10):e0240205)	Thanks for valid comment. I have used this reference on the symptomatic transmission risk; and a second reference to substantiate the presympromatic transmission risk.<br><br>What matters in all cases is managing the source of COVID-19. Known cases not using hygiene, physical distance, mask, not isolated will obviously transmit to the nearby population. However, pre-symptomatic and asymptomatic are silent transmitters as the patient himself does not recognize that he has COVID-19.<br><br>We appreciate the comment
5	need to refine what part of the WHO document to which you are referring nce 14 and the Jefferson review listed as reference 23 has been updated to new date from Nov 2020.	Thanks for this comment. We have updated the WHO reference with the title, “Considerations for implementing and adjusting public health and social measures in the context of COVID-19.<br><br>In addition, we have included updated reference Jefersson, T et al, Nov 2020, the uncertainties to physical distancing and mask using in the discussion

6	The Discussion is too long and unfocused in areas and needs to be tightened and agree with the comments of Reviewer 1 in this context. The references are not in the standard format for the Journal and case, formatting, access dates and full urls must be provided.	The discussion has multiple items. We have tried to focus, a bit in-depth. We could not cut the extent of discussion, however.<br><br>The reference format has now followed the PLOS One format. Used [} format. All Web pages have now URL.
7	You are strongly recommended to have someone whose native language is English review since there are many grammatical and syntax errors and it impacts negatively on the readability.	Thanks for comment. Do not blame us much, as our initial focus was to bring the science on a Table. We very much appreciate your gesture of looking at this relevant MS.<br><br>We have now edited aggressively using online checkers and some one among us who has an excellent English language
8	A rebuttal letter that responds to each point raised by the academic editor and reviewer(s). You should upload this letter as a separate file labeled 'Response to Reviewers'.	We have done this.
9	A marked-up copy of your manuscript that highlights changes made to the original version. You should upload this as a separate file labeled 'Revised Manuscript with Track Changes'.<br><br>An unmarked version of your revised paper without tracked changes. You should upload this as a separate file labeled 'Manuscript'.	Two revised versions are attached:<br>1. Tracked word file with edits<br>2. And cleaned one with no track changes
10	If applicable, we recommend that you deposit your laboratory protocols in protocols.io to enhance the reproducibility of your results.	What we mention a protocol in the manuscript is just a working guide or SOP for the application of data collection. It is uploaded as a supplemental file, although adequate description of data collection was indicated in the Monitoring protocol section.
11	Tables and Figures formatting	Tables have now only heading and last raw lines<br>Fig 2 and 3 were a bit reformatted to relive the crowding
	**Comments on Journal Requirements**	

1	Please ensure that your manuscript meets PLOS ONE's style requirements, including those for file naming. The PLOS ONE style templates can be found at<br><br>https://journals.plos.org/plosone/s/file?id=wjVg/PLOSOne_formatting_sample_main_body.pdf and<br><br>https://journals.plos.org/plosone/s/file?id=ba62/PLOSOne_formatting_sample_title_authors_affiliations.pdf	We have made citations in brackets using Plos One style. I had a bit difficulties, but made it<br><br>Many thanks for the link. The PLOS ONE text writing format guideline was used for both the abstract and body
2	2. Please provide additional details regarding participant consent. In the ethics statement in the Methods and online submission information, please ensure that you have specified (a) whether consent was informed and (b) what type you obtained (for instance, written or verbal, and if verbal, how it was documented and witnessed). If your study included minors, state whether you obtained consent from parents or guardians. If the need for consent was waived by the ethics committee, please include this information.	Additional details were included in the ethical consideration section. We did not seek a consent and we had a permission from IRB for this.
3	3. Thank you for stating the following in the Acknowledgments Section of your manuscript:<br><br>"The data collection was funded by the School of Public Health of Addis Ababa University. The Authors have contributed professionally in view of supporting the<br>Ministry of Health in enhancing proactive informed decisions."<br><br>We note that you have provided funding information that is not currently declared in your Funding Statement. However, funding information should not appear in the Acknowledgments section or other areas of your	We have now removed and agreed with your suggestion<br><br><br>We have modified the cover letter

	manuscript. We will only publish funding information present in the Funding Statement section of the online submission form.<br><br>Please remove any funding-related text from the manuscript and let us know how you would like to update your Funding Statement. Currently, your Funding Statement reads as follows:<br><br>"The author(s) received no specific funding for this work."<br><br>Please include your amended statements within your cover letter; we will change the online submission form on your behalf.	
4	Please amend the manuscript submission data (via Edit Submission) to include author Abera Kumie.	
5	Please include your tables as part of your main manuscript and remove the individual files. Please note that supplementary tables (should remain/ be uploaded) as separate "supporting information" files	We have done this
6	Please include captions for your Supporting Information files at the end of your manuscript, and update any in-text citations to match accordingly. Please see our Supporting Information guidelines for more information: http://journals.plos.org/plosone/s/supporting-information.	
