# Supplementary material for: Trends in non-pharmaceutical intervention (NPI) related community practice for the prevention of COVID-19 in Addis Ababa, Ethiopia
Source: PLoS One. 2021 Nov 23;16(11):e0259229. doi: 10.1371/journal.pone.0259229 (PMC8610281; doi:10.1371/journal.pone.0259229)
Supplement: Attachment — Submitted filename: PONE-D-21-03031_R2 Ehtiopia editor edits.pdf [file pone.0259229.s009.pdf]

PLOS ONE

Trends in non-pharmaceutical intervention (NPI) related community practice for the prevention of COVID-19 in Addis Ababa, Ethiopia

--Manuscript Draft--

**Manuscript Number:**	PONE-D-21-03031R2
**Article Type:**	Research Article
**Full Title:**	Trends in non-pharmaceutical intervention (NPI) related community practice for the prevention of COVID-19 in Addis Ababa, Ethiopia
**Short Title:**	Community practice for the prevention of Covid-19
**Corresponding Author:**	Yifoker Tefera<br>Addis Ababa University<br>aDDIS ABABA, ETHIOPIA ETHIOPIA
**Keywords:**	COVID-19; non-pharmaceutical intervention; community practice; COVID-19 Prevention
**Abstract:**	Background: The COVID-19 pandemic affected Ethiopia since March 13, 2020, when the first case was detected in Addis Ababa. Since then, the incidence of cases has continued to increase day by day. As a result, the health sector has recommended universal preventive measures to be practiced by the public. However, studies on adherence to these preventive measures are limited to inform evidence-based policy-making interventions.<br><br>Objective: To monitor the status of preventive practices of the population related to hand washing, physical distancing, and respiratory hygiene practices at selected sites within the city of Addis Ababa.<br>Methods: A weekly cross-sectional non-participatory observation was done during the period of April-June, 2020. Data was collected using Open Data Kit (ODK) tool in ten public sites involving eight public facilities targeted for individual observations. Ten individuals were randomly observed at each facility over two days a week at peak hours of public services WHO operational definitions of the preventive behaviors were adopted for this study. Observations were anonymously fixed at gates or entrances of public facilities and places.<br>Results : 12,056 individual observations with 53% males and 82% in 18-50 years age group were involved in this study. There was an increase in the practice of respiratory hygiene (from 14% in week one to 77% in week 10), while those of hand hygiene and physical distancing changed little over the weeks from their baseline of 24% and 34%, respectively. Overall, respiratory hygiene has shown an increased rate of 6% per week, while hand hygiene and physical distancing had <1%. Females and age group 18-50 years had increased practice in respiratory hygiene with no difference in the two practices. Respiratory hygiene took about six weeks to reach a level of 77% from its baseline of 24%, making an increment of about 9% per week.<br>Conclusion: The public practice of respiratory hygiene was relatively better than hand hygiene and physical distancing. It has shown visible weekly changes towards an increasing trend. Regularly sustained public mobilization and mass education are required to sustain the achievements gained in respiratory hygiene and improving hand hygiene and physical distancing.
**Order of Authors:**	Abera Kumie
	Damen Hailemariam
	Samson Wakuma
	Yifoker Tefera
	Teferi Abegaz
	Worku Tefera
	Mulugeta Tamire
	Wondimu Ayele

Powered by Editorial Manager® and ProduXion Manager® from Aries Systems Corporation

	Shibabaw Yirsaw
**Opposed Reviewers:**	
**Response to Reviewers:**	We have all accomdated the comments
**Additional Information:**	
**Question**	**Response**
**Financial Disclosure**<br><br>Enter a financial disclosure statement that describes the sources of funding for the work included in this submission. Review the submission guidelines for detailed requirements. View published research articles from *PLOS ONE* for specific examples.<br><br>This statement is required for submission and **will appear in the published article** if the submission is accepted. Please make sure it is accurate.<br><br>**Unfunded studies**<br>Enter: *The author(s) received no specific funding for this work.*<br><br>**Funded studies**<br>Enter a statement with the following details:<br>• Initials of the authors who received each award<br>• Grant numbers awarded to each author<br>• The full name of each funder<br>• URL of each funder website<br>• Did the sponsors or funders play any role in the study design, data collection and analysis, decision to publish, or preparation of the manuscript?<br>• **NO** - Include this sentence at the end of your statement: *The funders had no role in study design, data collection and analysis, decision to publish, or preparation of the manuscript.*<br>• **YES** - Specify the role(s) played.<br><br>* typeset	The author(s) received no specific funding for this work.
**Competing Interests**<br><br>Use the instructions below to enter a competing interest statement for this submission. On behalf of all authors, disclose any competing interests that could be perceived to bias this	The authors have declared that no competing interests exist.

work—acknowledging all financial support and any other relevant financial or non-financial competing interests.<br><br>This statement is required for submission and **will appear in the published article** if the submission is accepted. Please make sure it is accurate and that any funding sources listed in your Funding Information later in the submission form are also declared in your Financial Disclosure statement.<br><br>View published research articles from *PLOS ONE* for specific examples.<br><br>**NO authors have competing interests**<br><br>Enter: *The authors have declared that no competing interests exist.*<br><br>**Authors with competing interests**<br><br>Enter competing interest details beginning with this statement:<br><br>*I have read the journal's policy and the authors of this manuscript have the following competing interests: [insert competing interests here]*<br><br>* typeset	
**Ethics Statement**<br><br>Enter an ethics statement for this submission. This statement is required if the study involved:<br><br>• Human participants<br>• Human specimens or tissue<br>• Vertebrate animals or cephalopods<br>• Vertebrate embryos or tissues<br>• Field research<br><br>Write "N/A" if the submission does not require an ethics statement.	Protocol No: 049/20/SPH<br><br>Institutional Review Board of the College of Health Sciences, Addis Ababa University

General guidance is provided below. Consult the submission guidelines for detailed instructions. **Make sure that all information entered here is included in the Methods section of the manuscript.**<br><br>**Format for specific study types**<br><br>**Human Subject Research (involving human participants and/or tissue)**<br>• Give the name of the institutional review board or ethics committee that approved the study<br>• Include the approval number and/or a statement indicating approval of this research<br>• Indicate the form of consent obtained (written/oral) or the reason that consent was not obtained (e.g. the data were analyzed anonymously)<br><br>**Animal Research (involving vertebrate animals, embryos or tissues)**<br>• Provide the name of the Institutional Animal Care and Use Committee (IACUC) or other relevant ethics board that reviewed the study protocol, and indicate whether they approved this research or granted a formal waiver of ethical approval<br>• Include an approval number if one was obtained<br>• If the study involved *non-human primates*, add *additional details* about animal welfare and steps taken to ameliorate suffering<br>• If anesthesia, euthanasia, or any kind of animal sacrifice is part of the study, include briefly which substances and/or methods were applied<br><br>**Field Research**<br><br>Include the following details if this study involves the collection of plant, animal, or other materials from a natural setting:<br>• Field permit number<br>• Name of the institution or relevant body that granted permission	
**Data Availability**<br><br>Authors are required to make all data underlying the findings described fully	Yes - all data are fully available without restriction

available, without restriction, and from the time of publication. PLOS allows rare exceptions to address legal and ethical concerns. See the PLOS Data Policy and FAQ for detailed information.<br><br>A Data Availability Statement describing where the data can be found is required at submission. Your answers to this question constitute the Data Availability Statement and **will be published in the article**, if accepted.<br><br>**Important:** Stating 'data available on request from the author' is not sufficient. If your data are only available upon request, select 'No' for the first question and explain your exceptional situation in the text box.<br><br>Do the authors confirm that all data underlying the findings described in their manuscript are fully available without restriction?	
**Describe where the data may be found in full sentences. If you are copying our sample text, replace any instances of XXX with the appropriate details.**<br><br>• If the data are **held or will be held in a public repository**, include URLs, accession numbers or DOIs. If this information will only be available after acceptance, indicate this by ticking the box below. For example: *All XXX files are available from the XXX database (accession number(s) XXX, XXX.*).<br>• If the data are all contained **within the manuscript and/or Supporting Information files**, enter the following: *All relevant data are within the manuscript and its Supporting Information files.*<br>• If neither of these applies but you are able to provide **details of access elsewhere**, with or without limitations, please do so. For example:<br><br>*Data cannot be shared publicly because of [XXX]. Data are available from the XXX Institutional Data Access / Ethics*	All relevant data are within the manuscript and its Supporting Information files.

Committee (contact via XXX) for researchers who meet the criteria for access to confidential data.<br><br>*The data underlying the results presented in the study are available from (include the name of the third party and contact information or URL).*<br>• This text is appropriate if the data are owned by a third party and authors do not have permission to share the data.<br><br>* typeset	
Additional data availability information:	

Powered by Editorial Manager® and ProduXion Manager® from Aries Systems Corporation

Cover Letter

Trends in non-pharmaceutical intervention (NPI) related community practice for the prevention of COVID-19 in Addis Ababa, Ethiopia

What this study adds

Our study involved a timely event that targets a pro-active policy decision for the prevention of COVID-19. We used a non-participatory method of observation that helps to collect valid data at population level. Weekly observation on weekly basis involving dedicated sites with public services was done to show the trend of the progress that can be with a mitigation measure in the absence of an active immunization program for COVID-19.

The study outcomes were planned to be used by the Ministry of Health here in Ethiopia. The study results were acknowledged and facilitated the raising of funds the Ministry of Health to continue the monitoring of personal protective behaviors up to present time. The Ministry is using the outcomes of data for social mobilization and consider as inputs for modelling the transmission in our setting.

Our study has not been previously published, either in whole or in part (unless as an abstract), and that no similar paper is in press or under review elsewhere to the best of our knowledge. The authors do not have any financial competing interests and no conflicts in this publication with any other person or institution. We ensure also that we do not have any closely related paper with this submission.

Source of a Fund: The data collection was funded by the School of Public Health of Addis Ababa University. The Authors have contributed professionally to support the Ministry of Health in enhancing proactive informed decisions.

Authors list: Damen Haile Mariam[1], Abera Kumie[1], Samson Wakuma[1], Yifoker Teferra[1], Teferi Abegaz[1], Worku Tefera[1], Wendimu Ayele[1], Mulugeta Tamire[1,] Shibabaw Yirsaw[1]

[1] School of Public Health, College of Health Sciences, Addis Ababa University

Manuscript

Click here to access/download;Manuscript;PLOS Manuscript_Re_Revised _Cleaned_DH4_AK_31Aug2021.docx

Research Article

Trends in non-pharmaceutical intervention (NPI) related community practice for the prevention of COVID-19 in Addis Ababa, Ethiopia

Authors List

Damen Haile Mariam[1], Abera Kumie[1*], Samson Wakuma[1], Yifoker Teferra[1], Teferi Abegaz[1], Worku Tefera[1], Wendimu Ayele[1], Mulugeta Tamire[1,] Shibabaw Yirsaw[1]

[1] School of Public Health, College of Health Sciences, Addis Ababa University

Corresponding Author: Abera Kumie, *[1]aberakumie2@yahoo.com

Abstract

Background: The COVID-19 pandemic affected Ethiopia since March 13, 2020, when the first case was detected in Addis Ababa. Since then, the incidence of cases has continued to increase day by day. As a result, the health sector has recommended universal preventive measures to be practiced by the public. However, studies on adherence to these preventive measures are limited.

Objective: To monitor the status of preventive practices of the population related to hand washing, physical distancing, and respiratory hygiene practices at selected sites within the city of Addis Ababa.

Methods: Weekly cross-sectional non-participatory observation LVZSKP*T were done during the period of April-June, 2020. Data was collected using the Open Data Kit (ODK) tool in ten public sites involving eight public facilities targeted for individual observations. Ten individuals were randomly observed at each facility over two days a week at peak hours of public services. WHO operational definitions of the preventive behaviors were adopted for this study. Observations were anonymously at gates or entrances of public facilities and places.

Results: A total of 12,056 individual observations with 53% males and 82% in an estimated age range of 18-50 years age group were involved in this study. There was an increase in the practice of respiratory hygiene from 14% in week one to 77% in week 10, while those of hand hygiene and physical distancing changed little over the weeks from their baseline of 24% and 34%, respectively. Overall, respiratory hygiene demonstrated an increased rate of 6% per week, while hand hygiene and physical distancing had less than a 1% change per week, Females and the

estimated age group of 18-50 years had practice in respiratory hygiene with no difference in hand hygiene and physical distancing. Respiratory hygiene took about six weeks to reach a level of 77% from its baseline of 24%, making an increment of about 9% per week.

Conclusion: The public practice of respiratory hygiene improved threefold whereas hand hygiene and physical distancing revealed no change. Regularly sustained public mobilization and mass education are required to sustain the achievements gained in respiratory hygiene and further hand hygiene and physical distancing.

Keywords: COVID-19, hand hygiene, physical distancing, respiratory hygiene, mask use.

Introduction

The COVID-19 pandemic was originally described in Wuhan, China, in Dec 2019 (1). Since then, it has spread to Europe, the USA, and later to Africa. The infection's nature of transmission is considered to be through droplets, , and contact transmission (2). The possibility of transmission through faeco-oral and airborne routes is still under investigation (3, 4). Pre-symptomatic cases facilitate some of the transmission for a household clustering of infection and during travels (5-7). Symptomatic infections appear to have the highest transmission potential compared to asymptomatic and presymptomatic when considering the transmission index (8). The mortality pattern is devastating among elder age groups because of limited body immunity (9, 10).

Infection prevention and control (IPC) measures for emerging infectious diseases, like COVID-19, in health care settings can be challenging, especially in outbreaks where resources are limited (11). Yet such measures, including early identification, prompt isolation, proper patient placement, adequate space ventilation, and proper use of personal protective equipment, are important in preventing and controlling the transmission of COVID-19 (12). In addition, community prevention activities are considered the best strategies to reduce the healthcare burden, as they help slow transmission of the virus in the general population (13). Community prevention strategies are a set of actions that persons and communities can implement to slow the transmission of COVID-19 by practicing hand hygiene, respiratory hygiene, and social distancing (14).

The Ministry of Health of Ethiopia provides daily updates on the number of new infections and reported mortalities associated with COVID-19 (https://www.worldometers.info/coronavirus/country/ethiopia). The first case in Ethiopia was declared on March 13, 2020, and the number of cases grew very slowly until the end of the first week of May 2020 (from 1 case on March 13 to 194 cases on 08 May 2020) (15). However, the number of

reported cases increased since then, reaching 110,074 cases and 1,706 deaths on 01 December 2020 (the time of the write-up of this manuscript) (16).

In Ethiopia, the Federal Ministry of Health (FMOH) and the Ethiopian Public Health Institute have created awareness about the virus transmission and prevention methods using different approaches, including mass media such as national television and radioduring the partial-lock down period of five months since April, 20/2021. The interventions taken by the government ranged from banning public gatherings (over four persons) to limiting the number of passengers on public transport. In addition, all public service rendering institutions (such as food establishments, marketplaces, banks, and health facilities) provided hand-washing facilities and maintained a physical distance of at least one meter between their clients. Furthermore, security forces and public authorities have shown direct involvement in maintaining mask use and maintaining physical distancing in public places such as marketplaces, public transport stations and terminals, and streets. Land borders were shut in an effort to control the entrance of the virus from abroad. These interventions have been associated with the decline of COVID-19 transmission in other countries (17) and that present the only effective methods in the absence of vaccines and medications. Data on monitoring the status of these interventions, however, is lacking in Ethiopia.

Our current study generates evidence on COVID-19 prevention practices of the residents of Addis Ababa (focusing on hand hygiene, physical distancing, and respiratory hygiene). The findings are expected to inform policy to sustain the related behaviors in the general population in our country.

Methods

Study design, Study Area, Setting, and Period

A cross-sectional observational design was conducted weekly between during April 24-June 28, 2020. Addis Ababa is the capital city of Ethiopia and is headquarters for the African Union and the Economic Commission for Africa. The city had a population of 5 million in Dec 2021 (18) and is sub-divided into ten administrative sub-cities. Sub-cities are divided into districts ("Wereda"). One of the biggest markets in Africa ("*Merkato*") is located in the heart of the city. There are about 131 Orthodox churches as well as several mosques. About 67% of the total vehicles (estimated to be about 800,000) are registered within Addis Ababa. There are several public and private banks in the city – including the Commercial Bank of Ethiopia (a state enterprise) that alone has over 120 branches in Addis Ababa.

This study was initiated as part of the Emergency Operation Center (EOC) preventive behavior monitoring activity at the College of Health Sciences (CHS), Addis Ababa University. CHS owns a teaching hospital that mandated an epidemic preparedness and management plan for its staff and visiting patients. The monitoring aimed to explore community practice levels regarding the three non-pharmaceutical interventions (NPI) for COVID-19 prevention including hand hygiene, physical distancing, and respiratory hygiene, in the ten administrative units (sub-cities) of Addis Ababa. The study was conducted by observing individual's behavior at selected sites when getting public services. These sites were selected based on the level of crowding and population mobility, and thus considered an indication of the risk for COVID-19 transmission.

Study population

The source population for the study was all individuals visiting the selected sites during the day of data collection. Study participants were individuals who visited the selected sites during the time of data collection.

Monitoring protocol

Before the data collection, a monitoring protocol had been prepared and approved by the research committee which was a standard operative procedure (SOP) used for the application of data collection. The protocol described the observation site selection, identified the list of public facilities to be observed, operationally defined the NPI practices, and the time and the procedure for the observation. First, "community center-public sites' were identified in each sub-city. These sites are characterized by having increased population mobility relative to the nearby neighborhoods. Then, one of the relevant sites was identified as a nucleus for the subsequent selection of government and public institutions and facilities for monitoring. Eight facility-sites were purposely selected based on selection criteria that included active public service provision, availability of adequate flow of service users, and appropriateness of the location for observation relative to increased population mobility. These sites represented religious places, health facilities, market places, banks, public transport, food and drink establishments, street crossing sites, and workplaces. Each public facility had ten observations.

We followed the protocol's provision in standardizing the context of observation. Fixing the distance of fewer than 10 meters from the observation point represented by the gate or entrance to the public services (buildings or open spaces like a traditional market) was maintained. This represents the distance that maintains the proximity of observation by the research assistant. The

observation of an individual seeking a public service was designed anonymously to get valid behaviours of an individual's practice in reference to hand hygiene, physical distance, and respiratory hygiene. Ethical approval was obtained for this purpose. The research assistant found a convenient place that allowed his/her visual field to be optimum for an observation. The observer used a mobile phone ODK to record data without being identified by any person. This helps to distract suspecting his work, hence maintaining neutrality and data validity. The observer kept moving within the proximity of the gate in case there might be any suspicion of being identified. The research assistant was instructed to spend about 15-30 minutes on one site, which prevented identification. Finally, only two days in a week, weekdays (Wednesdays) and weekends (Sundays) were used for data collection to have weighted data represented by working days and holidays. The provisions of the context of monitoring are indicated in the ~~support~~ information (S1)

Data collection tools

We prepared a data collection tool that had two sub-sections (attached in a supplementary file). Section 1 addressed the location characteristics, while section 2 recorded the practice of the individual. The location checklist addressed information regarding the time of observation, the Global Positioning System (GPS) of each service facility location, the presence of any enforcement, and the availability of handwashing facilities, including water and detergents. The NPI practice section contained sex, estimated age, NPI practice in three categories (proper, improper, and none). Each NPI practice was operationally defined within the monitoring protocol following the WHO recommendation (19) (S2).

Data collection procedure

One trained research assistant was assigned per sub-city, and one supervisor was appointed for two data collectors. The supervisor communicated with the field research assistant on each day of data collection to discuss the context and challenges of data collection. The field data were collected using the Open Data Kit tool (ODK) at the peak hours when the number of people was assumed to be highest at each observation site as stated in the protocol (Fig 1). The recorded ODK data were uploaded daily to the central server.

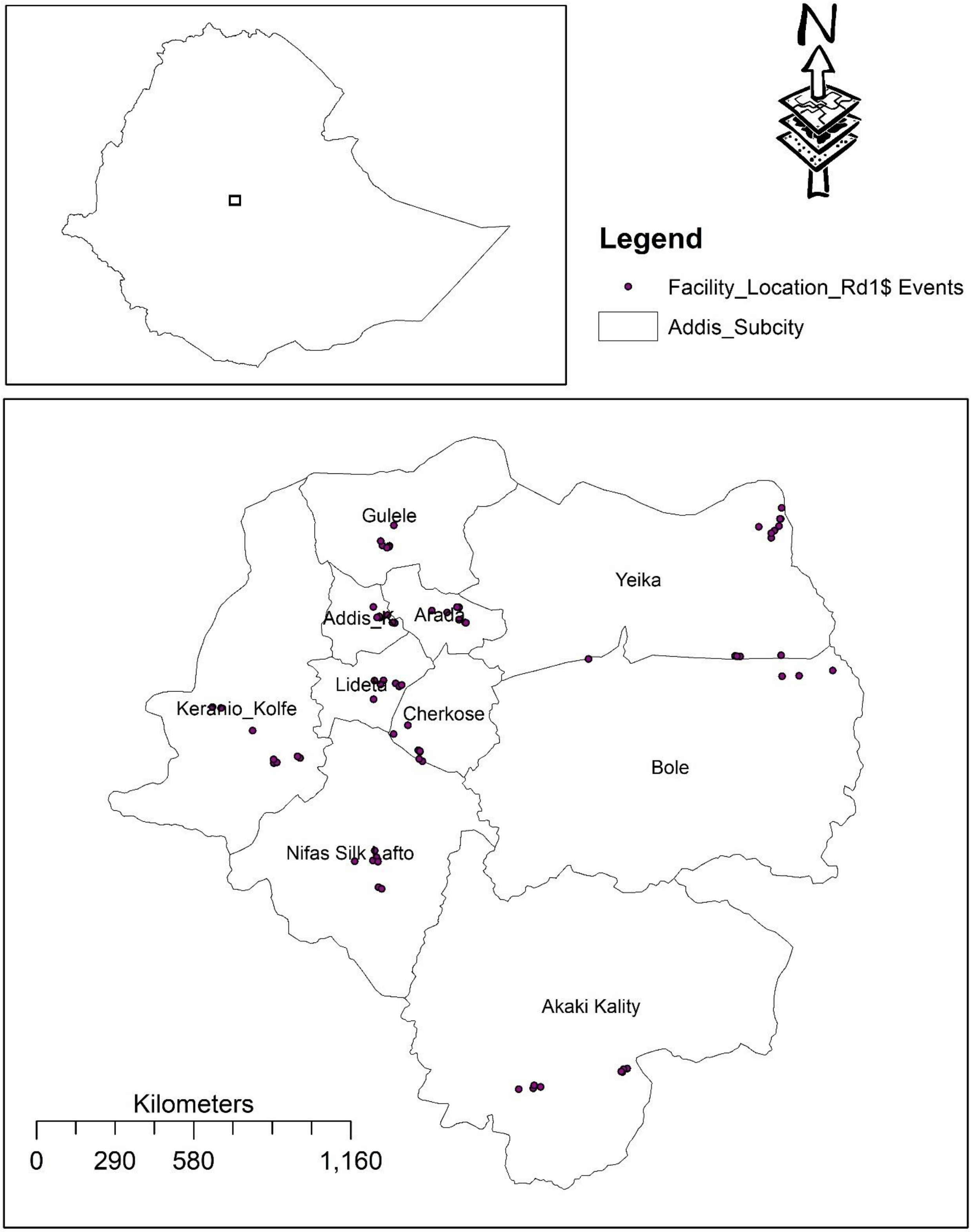

Figure 1: Observational data collection sites in Addis Ababa

Data management and analysis

Data from the main server were downloaded into Excel (Microsoft Corp.) and transferred into SPSS V 23 for data merging, cleaning, and analysis. Data were presented using descriptive statistics by calculating the proportion of practices for each week. The trend of the practice was evaluated using weekly differences in proportions (a current week with the previous week). The long-term trend was evaluated by visualizing the line graph and looking at the overall average of weekly differences. Limited statistical tests were done to see the significance of the overall practice with sex and the estimated age group. The weekly difference in the three practices was checked using a non-parametric Kruskal Wallis test. The average of differences between each week was considered to estimate the strength of the overall weekly change. Tables, line graphs, and charts were used to present data. The data set is in the supplemental support information (S3).

Ethical considerations

The study received ethical approval from the Institutional Review Board (IRB) of the College of Health Sciences at Addis Ababa University. We had permission from IRB not to have consent as the results of the observations were meant to benefit the public at large and we did not seek any identifying information or photos of any individuals. (S4). The survey used a random observation tool that does not override the rights of any individual or institution, and the observation was done purely anonymously. A weekly report on the progress of monitoring was submitted to the Ministry of Health of Ethiopia to help to take proactive actions.

Results

Study participants

A total of 12,056 people were observed from April 20 to June 28, 2020. More than half of these (59.2%) were observed on the weekdays (Table 1). We had 6,383 (52.9%) males and 5,673 (47.1%) females in the study. The majority, 9832 (81.6%), were in the estimated age group of 18-50 years.

Table 1: Number of people observed (April 20– June 14, 2020, Addis Ababa)

Observation week	Weekdays observations # (%)	Weekend observations # (%)	Total observations # (%)
Week 1 (Apr20-26)	225 (26.3)	629 (73.7)	854 (7.1)
Week 2 (Apr27-May3)	723 (62.1)	442 (37.9)	1,165 (9.7)
Week 3 (May 4-May 10)	804 (63.6)	460 (36.4)	1264 (10.5)
Week 4 (May 11-May 17)	719 (60.0)	480 (40.0)	1,199 (9.9)
Week 5 (May 20- May 24)	775 (62.2)	470 (37.8)	1,245 (10.3)
Week 6 (May 27-May 31)	706 (59.1)	488 (40.9)	1,194 (9.9)
Week 7 (June 3-June 7)	799(62.0)	489 (38.0)	1,288 (10.7)
Week 8 (June 10-June 14)	788(61.7)	490 (38.3)	1,278 (10.6)
Week 9 (June 15- June 21)	802 (62.6)	479 (37.4)	1,281 (10.6)
Week 10 (June 22- June 28)	798 (62.0)	490 (38.0)	1,288 (10.7)
**Total**	**7,139 (59.2)**	**4,917 (40.8)**	**12,056 (100)**

The overall trend of NPI community practice

Surgical masks predominated, (51%) relative to cloth masks, (41%), N95, (4%), and scarf, (4%). Proper hand hygiene in the community slowly increased from the baseline of 24.2% in week one to 32.5% in week three and then slowly declined to 23.7% in week 10. Similarly, proper physical distancing in the community increased from 34.4% in week one to 42.6 % in week four and then slowly declined to 35.2 % in week eight. On the contrary, proper respiratory hygiene in the community continuously increased from 14% in week one to 77% in week ten.

Driver's respiratory hygiene continuously increased from 13.8 % in week one to 84.6 % in week 10. Driver's assistants had 0% respiratory hygiene practice in week one but increased to 70.5% in week six, then slowly declined to 54.1% in week 10 (Table 2). The results also showed that adherence to the required vehicle occupancy less than or equal to 50% occupancy rate in commercial vehicles declined from 88.8% in week one to 68.8 % in week 10. There was a sharp rise in the practice of respiratory hygiene, while those of hand hygiene and physical distancing did not change or declined during the last week of observation (Fig 2). Week 4 was the turning point to observe an increase, while the highest mask used in a population took seven weeks. The trend in hand hygiene and physically distancing through the ten weeks was stable relative to respiratory hygiene.

The three combined hand and respiratory hygiene practices with physical distancing managed by an individual were below 10% in any week. It increased slowly from 4.7% in week one to 10.8% in week four and then declined to 8.1% in week 10.

Varation in practices

Overall, we have found statistical differences for the three practices ($p<0.05$), and within-subject differences by sex, age, and day of observation ($P<0.05$). Females and age category of 18-50 years had statistical differences in the practice for respiratory hygiene. The practices observed in the week-days had an increased proportion over the week-end days ($p<0.05$) for hand and respiratory hygiene.

Table 2: Weekly pattern of a community practice of non-pharmaceutical interventions (Addis Ababa, 2020)

NPI	NPI Practices	Week 1 (Apr20-26)	Week 2 (Apr27-May3)	Week 3 (May 4-10)	Week 4 (May 11- 17)	Week 5 (May20- 24)	Week 6 (May 27-31)	Week 7 (June 3-7)	Week 8 (June10-14)	Week 9 (June15-21)	Week 10 June 22-28
Hand hygiene	**Proper hand hygiene**	**178 (24.2)**	**328 (33.1)**	**350 (32.5)**	**323 (32.0)**	**305 (28.8)**	**26 1 (25.9)**	**288 (26.1)**	**290 (26.8)**	**281 (28.5)**	**254 (23.7)**
	Improper hand hygiene	88 (11.9)	93 (9.4)	101 (9.4)	76 (7.5)	78 (7.4)	80 (7.9)	121 (10.9)	105 (9.7)	123 (12.4)	103 (9.6)
	No hand hygiene	469 (63.8)	571 (57.6)	625 (58.1)	610 (60.4)	674 (63.8	667 (66.2)	692 (62.8)	684 (63.4)	582 (59.1)	715 (66.7)
	**Total**	**735**	**992**	**1076**	**1009**	**1057**	**1008**	**1101**	**1079**	**986**	**1072**
Physical distance	**Proper physical distance**	**294 (34.4)**	**462 (39.7)**	**491 (38.8)**	**511 (42.6)**	**46 7 (37.5)**	**444 (37.2)**	**479 (37.2)**	**496 (38.8)**	**503 (39.3)**	**454 (35.2)**
	Improper physical distance	408 (47.8)	531 (45.6)	593 (46.9)	551 (46.0)	655 (52.6)	643 (53.9)	699 (54.3)	662 (51.8)	711 (55.5)	710(55.1)
	No physical distance	152 (17.8)	172 (14.8)	180 (14.2)	137 (11.4)	123 (9.9)	107 (9.0)	110 (8.5)	120 (9.4)	67 (5.2)	124(9.6)
	**Total**	**854**	**1165**	**1264**	**1199**	**1245**	**1194**	**1288**	**1278**	**1281**	**1288**
Respiratory hygiene	**Proper respiratory hygiene**	**205 (24.0)**	**301 (25.8)**	**405 (32.0)**	**575 (48.0)**	**746 (59.9)**	**845 (70.8)**	**985 (76.5)**	**980 (76.7)**	**999 (78.0)**	**995 (77.3)**
	Improper respiratory hygiene	79 (9.3)	174 (14.9)	213 (16.9)	271 (22.6)	151 (12.1)	153 (12.8)	198 (15.4)	214 (16.7)	208 (16.2)	201 (15.6)
	No respiratory hygiene	570 (66.7)	690 (59.2)	646 (51.1)	353 (29.4)	348 (28.0)	196 (16.4)	105 (8.2)	84 (6.6)	74 (5.8)	92 (7.1)
	**Total**	**854**	**1165**	**1264**	**1199**	**1245**	**1194**	**1288**	**1278**	**1281**	**1288**
Drivers' respiratory hygiene	**Proper respiratory hygiene**	**11 (13.8)**	**34 (23.0)**	**91 (55.2)**	**133 (78.7)**	**159 (84.6)**	**170 (89.5)**	**167(84.8)**	**165(82.9)**	**156(82.5)**	**168 (84.6)**
	Improper respiratory hygiene	1 (1.3)	11 (7.4)	15 (9.1)	11 (6.5)	23 (12.2)	17 (8.9)	29 (14.7)	30 (15.1)	30 (15.9)	27 (13.6)
	No respiratory hygiene	68 (85.0)	103 (69.6)	59 (35.8)	26 (15.3)	6 (3.2)	3 (1.6)	1 (0.5)	4 (2.0)	3 (1.6)	4 (2.0)
	**Total**	**80**	**148**	**165**	**170**	**188**	**190**	**197**	**199**	**189**	**199**
Driver assistants' respiratory hygiene	**Proper respiratory hygiene**	**0%**	**29 (19.6)**	**60 (37.3)**	**87 (52.1)**	**119 (62.6)**	**141 (70.5)**	**135 (69.9)**	**118 (60.5)**	**111 (59.0)**	**106 (54.1)**
	Improper respiratory hygiene	3(4.0)	19(12.8)	29 (18.0)	44 (26.3)	61 (32.1)	48 (24.0)	57 (29.5)	72 (36.9)	73 (38.8)	80 (40.8)
	No respiratory hygiene	72 (96.0)	100 (67.6)	72 (44.7)	36 (21.6)	10 (5.3)	11 (5.5)	1 (0.5)	5 (2.6)	4 (2.1)	10 (5.1)
	**Total**	**75**	**148**	**161**	**167**	**190**	**200**	**193**	**195**	**188**	**196**
Vehicle occupant capacity	**Less than or equal to 50% capacity**	**71 (88.8)**	**122 (83.0)**	**114 (69.1)**	**110 (65.1)**	**128 (68.1)**	**128 (67.4)**	**136(69.0)**	**140(70.4)**	**132(69.8)**	**137(68.8)**
	Greater than 50% capacity	9 (11.3)	25 (17.0)	51 (30.9)	59 (34.9)	60 (31.9)	62 (32.6)	61 (31.0)	59 (29.6)	57 (30.2)	62 (31.2)
	**Total**	**80**	**147**	**165**	**169**	**188**	**190**	**197**	**199**	**189**	**199**

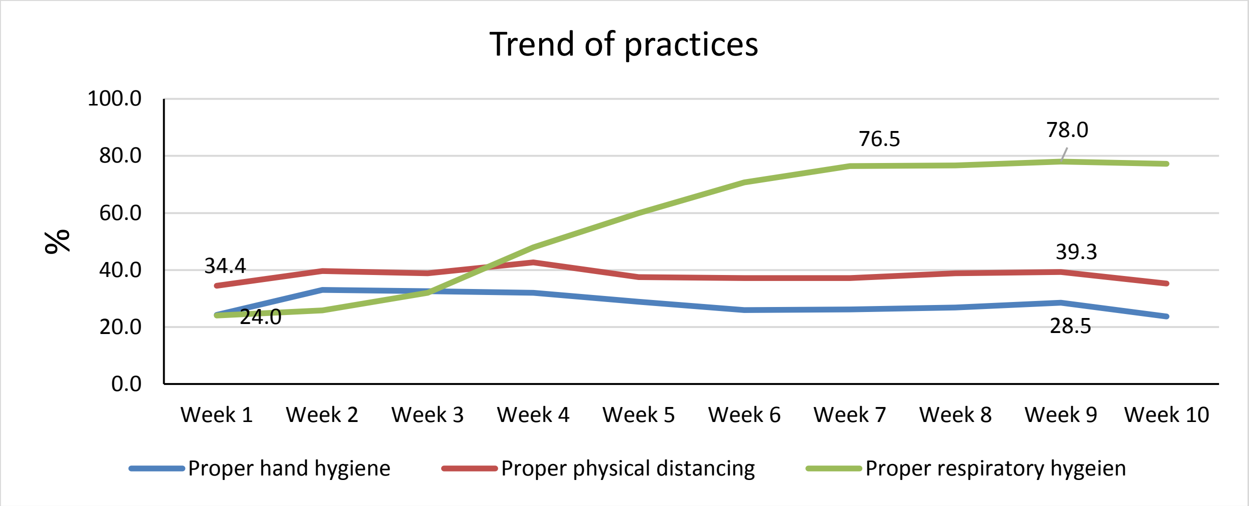

Figure 2: The overall trend of the proper community practices for the three NPIs

The trend of respiratory hygiene by observation facilities

There was a similar pattern of an increasing trend in all eight facilities with varying intensity of weekly increases. Banks and health facilities showed fast and stable growth rates over the ten weeks, from 30% in week one to 90% in week ten and from 30% in week 1 to 86% in week 10, respectively (Fig 3).

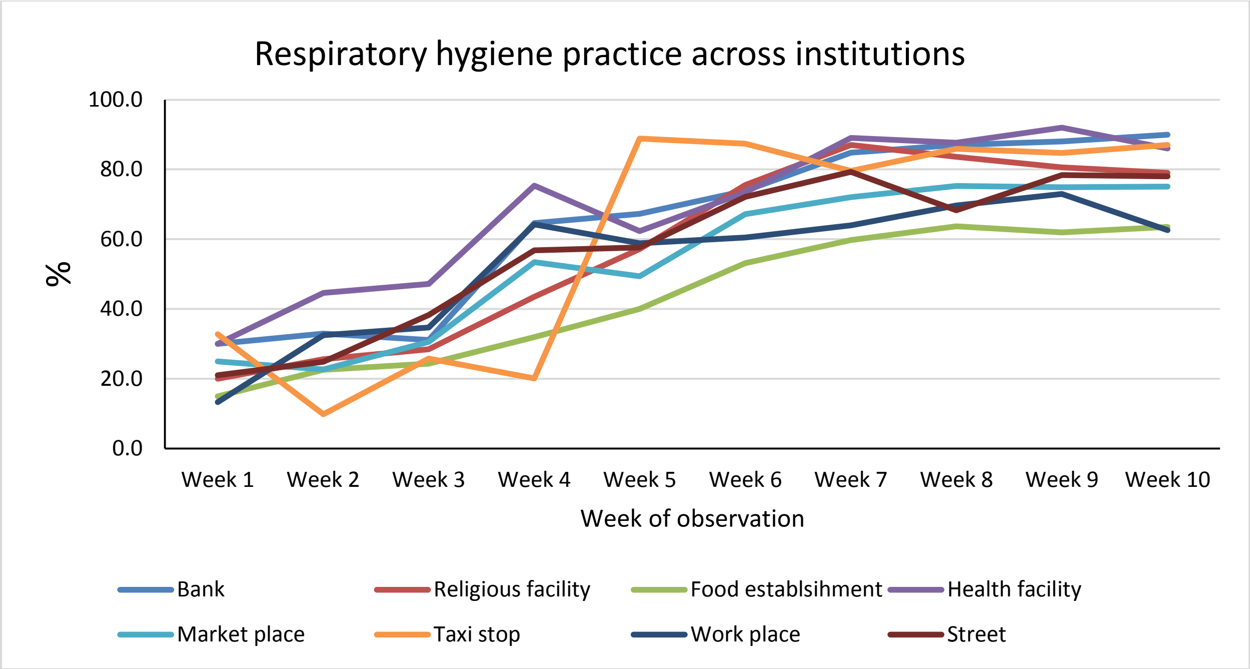

Figure 3: The trend of the proper respiratory hygiene practices by public facilities

Discussion

Non-pharmaceutical interventions are the primary preventions to restrict the spread of COVID-19 in the setting of limited access to vaccines. Overall, our results demonstrated that the use of masks was stable at around 80%, while hand hygiene and physical distancing were not observed to go beyond 40%. The optimum application of the three practices was limited in our study and only attained for the respiratory hygiene. The World Health Organization greatly acknowledged the combined practice of non-pharmaceutical interventions as a frontline tool to combat the infection (19, 20) .The non-pharmaceutical interventions were effective in containing the outbreak demonstrated by the number of cases that could had been 67-fold higher at the time without these interventions (20). Systematic reviews have shown the frequent practice of hand hygiene, the use of physical distancing of 1 m or more, and mask utilization have a potential benefit of lowering the COVID-19 transmission (21, 22). These interventions are effective with optimum compliance if not universal in a community as has been strongly proposed in China for a high-risk population (23). The Nigerian Medical Association strongly criticized the limited use of non-pharmaceutical prevention protocols by citizens (24). The low level of mask using was mainly attributed to risky social behaviors, perceived risk of transmission, gender and age differences (25). This study also showed the variation of the mask use over time across many countries: some having gradually increased, others showing a declining trend. The community's access to affordable masks in our study should have provided a sense of protection compared to hand hygiene and physical distancing. The use of cloth masks that are easily accessible, cheap, and existing in many different options has provided a degree of protection next to surgical masks.

The present study has shown major improvements in the practice of respiratory hygiene compared to hand hygiene and physical distance during the period of our observation. The improvement started to show significant change after week five matched with when security forces started to enforce primary preventions in response to the state of emergency related to the epidemic crisis in Ethiopia. Although the state of emergency was declared on 20 April 2020, the full breadth of the impact came later, several weeks later of massive promotional provisions targeting the public at large and public service rendering institutions in particular. At the time of the study, the State Emergency in Ethiopia was characterized to have partial lock-down of public places, including the closing of educational facilities and workplaces coupled with strict use of masks at public places, such as transport and public service places, which resulted in a rapid increase during our monitoring period. According to the state of emergency provisions, public mobility was not restricted to seeking food, transport, health care, and marketing services. There are reports from other countries that NPI interventions during lock-down periods indicating them as effective tools to interrupt COVID-19 transmission (26, 27), although the benefit of physical distancing and masking has been uncertain due to lack of strong study designs (28). The state of emergency mandated private and government service providers to avail handwashing facilities with detergents for their clients who seek services. Handwashing facilities were usually placed at the gate or entrance of the institution to encourage hand hygiene before accessing the service. The perception of self-protections by using masks might have contributed towards the increasing practice. On the contrary, a small portion of the public, about 20%, did not use a mask that might be linked to the negative attitudes towards mask ineffectiveness to reduce the transmission (29).

Despite the p public enforcement, the practice of proper hand hygiene did not show much change over the ten weeks of observation and did not go beyond 30%. Two reasons were suggested from our observation. Firstly, the media company and involvement of the enforcing volunteers had massive promotions in the first four weeks during our data collection period around churches, transport stations, and other public places. This may have contributed to a change in handwashing practice in those weeks but not much beyond these weeks. On the other hand, the unavailability of water and soap could not be sustained in many facilities, except few public services providing institutions such as banks and health facilities. Generally, the provision of soap was initially challenging, which was followed by a shortage and total disappearance of water in many public service providers. We believe these challenges happened at the same time of weak enforcement when security and other partners discontinued the initiated interventions. There was a shift from washing hands to using hand sanitizer by some members of the population, especially the youth. Our observations had limited coverage of the youth; thus, we might have underestimated the proportion of handwashing in a community. The youths had limited needs for seeking services in facilities that were involved in our study.

The maintenance of physical distancing at a minimum of one meter between any two individuals had a similar trend with handwashing practice. However, the former had an overall performance of below 40% relative to the 30% of the later practices. Physical distancing was challenging for social services, predominantly observed in open markets, walking and crossing on a street, and seeking public transport sectors. Individuals in an open market traditionally tend to cluster around a seller to make transactions. Seeking public transport services at peak hours makes service seekers get closer around the gate of transport vehicles. The duration of exposure and physical distancing has been shown to be an important predictor of the transmission of SARS-CoV-1. (30). A systematic

review of 172 studies reported that physical distancing of one meter was associated with a large reduction in the number of new infections in different countries (21). Taking the economic status of Ethiopia, the lack of change in the physical distancing at the public level in this study was a missed opportunity to avert the occurrence of new cases in the city. Hand hygiene and physical distancing are effective, low-cost interventions that could reduce the number of new cases (17, 31). We believe limited adherence in hand hygiene and physical distancing are missed opportunities to avert new infections in a community.

Ethiopia possesses traditions that encourage the community to get together, such as burial events, traditional religious festivals, attending churches, and traditional open markets. The State emergency was not universal in the restriction of mass mobility during its active implementation phase, which might be a factor for the spread of the infection. Clients were advised to perform the three practices strictly during the initial wave of the epidemic. The enforcement of NPIs became weaker, however, a week after lifting off the state emergency. There were incidences during the socio-cultural events when physical distancing was ignored because of the population's crowding, such as religious holidays. Contrary with this finding, a study from China reported that almost all respondents avoided going to public places and reduced celebrating spring festivals due to the COVID-19 epidemic (32). The possible reasons for the difference in adherence might be associated with the socio-cultural contexts in a given country and the degree of enforcing preventive practices.

The extent and trend of mask utilization had visible differences compared to physical distancing and hand hygiene. Mask utilization was observed to have an increasing trend until it stabilized at

about 80% towards the end of our study time. The continued security enforcement, mass media mobilization, the restrictions of public services without a mask, and the provisions of regular information about COVID-19 using national TVs and radios were the main contributing factors. The multiple sources of information and public enforcement were believed to enhance the attitude of service seekers, which greatly modified their behavior towards the use of a mask. We cannot deny that access to multiple types of masks, such as surgical, N95, and cloth masks and their local production at affordable prices has critically sustained the trend of mask utilization. The local production of face masks in low-middle income countries has been indicated as a strategy to contain the epidemic (33). Before or after the state of emergency, the time of the observation and the age of study subjects could make a difference in the proportion of mask using. There wad a preference of using masks among the young population in Poland, 60%, a study undertaken before the government's enforcement (34, 35), which is relatively lower than ours.. Our study was undertaken after the declaration of State Emergency, which has brought multiple interventions to impact the behaviors of individuals towards using a mask. Overall, the universal use of masks in public has been considered as a means to reduce the transmission of respiratory viruses COVID-19 in previous studies (36, 37).

We disaggregated the mask by type of facilities to get insights about the factors affecting mask using. The trend of respiratory hygiene practice visibly increased among service providers involving health care facilities, banks, and transport services compared with other facilities included in this study. There were assigned controllers and guards checking for the use of face-masks and maintenance of physical distancing and hand hygiene, who did not allow individuals to get a public service without these practices.

The relatively improved practices at bank and health facilities may have been related to the fact that these institutions have committed the resources to provide handwashing facilities with detergents and that their clients would have a relatively higher level of awareness and favourable attitude towards the preventive practices As there are variabilities in people's beliefs about the effectiveness as well as the rates of engagement in the use of non-pharmaceutical interventions, public health efforts should focus on increasing perceived severity and threats of the COVID-19 while promoting the preventive measures as effective tools (36, 38) and enforcing the use of them as necessary. On the contrary, the adherence to mask using and physical distancing in open markets and food establishments will likely continue to exist as a challenge. Food and drink establishments require close attention because people eat in groups, and physical distancing was never practically applied. Onsite dining with indoor and outdoor seats was identified as a risky situation by the CDC (13).

A strength of our study was observing individuals randomly and anonymously at times of service provisions during peak hours in key social providing services. This approach would have reduced respondent's bias. The provision of weekly information to the Ministry of Health was an important input to enhance informed decisions to the public. We assumed observing individuals at the time of possible transmission in the presence of increased mobility and crowding provided better insights into the possibility of the transmission potential. We recognize the limitations of our study. We could not determine the presence of interview bias that could happen over time, although we had regular supervisions and meetings with research assistants. The data collected was for a limited time observation and may not represent the profile individual's behavior on a given day. We recognize the accuracy and reproducibility of age categories used are only estimates and variance

was likely in this regard. Finally, our study cannot predict the trends in community practice across the whole country in the absence of state wide interventions.

Conclusions and recommendations

The overall community prevention practices for the ~~of~~ COVID-19 pandemic were very low in Addis Ababa, Ethiopia, although respiratory hygiene singly showed a relatively increased rate. Despite the relatively low adherence of the community to proper handwashing and proper physical distancing, the success of proper respiratory hygiene was encouraging. Strengthening social mobilization using available media and consistent enforcement mechanisms to get sustained performances in community practices would be of value.

Acknowledgments: The authors are grateful to the College of Health Sciences and the School of Public Health of Addis Ababa University for allowing this study to happen. The leadership of Dr Dawit Wendimagegn, who is the Chief Executive Director of the College of Health Sciences is greatly thanked for his support to undertake this study.

Competing interest: We do not have any competing financial interests or conflicts in this publication with any other person or institution.

Author's Contributions

DH tabled a research idea on the need to generate the status of community practice. AK was involved in the writing of the proposal and drafting the manuscript. AK and SW were involved in the analysis of data. MT was involved in handling the qualitative field information. WA was involved in the design of the data template in ODK. YT and TA were involved in the design of observation tools. SY was involved in coordinating the data collection. Finally, all were involved in editing the proposal and manuscript.

References

1. Chen X, Yu B. First two months of the 2019 Coronavirus Disease (COVID-19) epidemic in China: real-time surveillance and evaluation with a second derivative model. Global health research and policy. 2020;5:7. Epub 2020/03/12. https://doi.org/10.1186/s41256-020-00137-4.
2. Wold Health Organization. Modes of transmission of virus causing COVID-19: implications for IPC precaution recommendations. News and press release. 2021 [cited 2021 December]; Available from: https://reliefweb.int/report/world/modes-transmission-virus-causing-covid-19-implications-ipc-precaution-recommendations?gclid=Cj0KCQjwssyJBhDXARIsAK98ITRhh2Gzq9h-GPShFQYBpV0WJoAIKyXYgnx-RPb_KxnvDil199rRVgMaAuYTEALw_wcB.
3. Beattie RM, Ashton JJ, Penman ID. COVID-19 and the gastrointestinal tract: emerging clinical data. Frontline gastroenterology. 2020;11(4):290-2. Epub 2020/06/26. https://fg.bmj.com/content/flgastro/early/2020/04/27/flgastro--101507.full.pdf.
4. Liu J, Liao X, Qian S, Yuan J, Wang F, Liu Y, et al. Community Transmission of Severe Acute Respiratory Syndrome Coronavirus 2, Shenzhen, China, 2020. Emerging infectious diseases. 2020;26(6):1320-3. Epub 2020/03/04.
5. Guoqing Q, Naibin Y, Ada HYM, Liping W, Guoxiang L, Xueqin C, et al. COVID-19 Transmission Within a Family Cluster by Presymptomatic Carriers in China Clinical Infectious Diseases 2020;71(15):861-2. doi: 10.1093/cid/ciaa316.
6. Qiu X, Nergiz AI, Maraolo AE, Bogoch, II, Low N, Cevik M. The role of asymptomatic and pre-symptomatic infection in SARS-CoV-2 transmission-a living systematic review. Clinical microbiology and infection : the official publication of the European Society of Clinical Microbiology and Infectious Diseases. 2021;27(4):511-9. Epub 2021/01/24. http://www.ncbi.nlm.nih.gov/pubmed/33484843.
7. Yu X, Yang R. COVID-19 transmission through asymptomatic carriers is a challenge to containment. Influenza Other Respir Viruses. 2020;14(4):474–5. Epub Epub 2020. https://pubmed.ncbi.nlm.nih.gov/32246886/.
8. Koh WC, Naing L, Chaw L, Rosledzana MA, Alikhan MF, Jamaludin SA, et al. What do we know about SARS-CoV-2 transmission? A systematic review and meta-analysis of the secondary attack rate and associated risk factors. PLoS One. 2020;15(10):e0240205. Epub 2020/10/09.
9. Dudley JP, Lee NT. Disparities in Age-specific Morbidity and Mortality From SARS-CoV-2 in China and the Republic of Korea. Clinical infectious diseases : an official publication of the Infectious Diseases Society of America. 2020;71(15):863-5. Epub 2020/04/02. http://www.ncbi.nlm.nih.gov/pubmed/32232322.
10. Palaiodimos L, Kokkinidis DG, Li W, Karamanis D, Ognibene J, Arora S, et al. Severe obesity, increasing age and male sex are independently associated with worse in-hospital outcomes, and higher in-hospital mortality, in a cohort of patients with COVID-19 in the Bronx, New York. Metabolism. 2020;108:154262. Epub 2020/05/19. http://www.ncbi.nlm.nih.gov/pubmed/32422233.
11. World Health Organization. Infection prevention and control of epidemic- and pandemic-prone acute respiratory infections in health care. 2014 [cited 2020 April 2]; Available from:

https://apps.who.int/iris/bitstream/handle/10665/112656/9789241507134_eng.pdf?sequence=1.

12. European Center for Diseases Prevention and Control. Infection prevention and control and preparedness for COVID-19 in healthcare settings. 2021 [cited 2020 April]; Available from: https://www.ecdc.europa.eu/sites/default/files/documents/Infection-prevention-control-for-the-care-of-patients-with-2019-nCoV-healthcare-settings_update-31-March-2020.pdf.
13. Centers for Diseases Control and Prevention. Implementation of Mitigation Strategies for Communities with Local COVID-19 Transmission. 2020 [cited 2020 April]; Available from: https://www.cdc.gov/coronavirus/2019-ncov/downloads/community-mitigation-strategy.pdf.
14. World Health Organization. Coronavirus disease (COVID-19) advice for the public. World Health Organization; 2019 [cited 2020 December]; Available from: https://www.who.int/emergencies/diseases/novel-coronavirus-2019/advice-for-public.
15. Worldometers. Ethiopia: Corona Virus Cases. 2020; Available from: https://www.worldometers.info/coronavirus/country/ethiopia/.
16. Pei S, Kandula S, Shaman J. Differential Effects of Intervention Timing on COVID-19 Spread in the United States. medRxiv : the preprint server for health sciences. 2020. Epub 2020/06/09. http://www.ncbi.nlm.nih.gov/pubmed/32511526.
17. Muller O, Neuhann F, Razum O. [Epidemiology and control of COVID-19]. Dtsch Med Wochenschr. 2020;145(10):670-4. Epub 2020/04/29. . Epidemiologie und Kontrollmassnahmen bei COVID-19. http://www.ncbi.nlm.nih.gov/pubmed/32344440.
18. United Nations. World Population Prospects. 2021; Available from: https://www.macrotrends.net/cities/20921/addis-ababa/population.
19. World Health Organization. Considerations for implementing and adjusting public health and social measures in the context of COVID-19. 2020; Available from: https://www.who.int/health-topics/coronavirus#tab=tab_2.
20. Lai S, Ruktanonchai NW, Zhou L, Prosper O, Luo W, Floyd JR, et al. Effect of non-pharmaceutical interventions to contain COVID-19 in China. Nature. 2020;585(7825):410-3. Epub 2020/05/05. http://www.ncbi.nlm.nih.gov/pubmed/32365354.
21. Chu DK, Akl EA, Duda S, Solo K, Yaacoub S, Schunemann HJ. Physical distancing, face masks, and eye protection to prevent person-to-person transmission of SARS-CoV-2 and COVID-19: a systematic review and meta-analysis. Lancet. 2020;395(10242):1973-87. Epub 2020/06/05. https://doi.org/10.5694/mja2.50602.
22. Jefferson T, Del Mar CB, Dooley L, Ferroni E, Al-Ansary LA, Bawazeer GA, et al. Physical interventions to interrupt or reduce the spread of respiratory viruses. The Cochrane database of systematic reviews. 2011(7):CD006207. Epub 2011/07/08. http://www.ncbi.nlm.nih.gov/pubmed/21735402.
23. Lau JT, Yang X, Tsui HY, Pang E. SARS related preventive and risk behaviours practised by Hong Kong-mainland China cross border travellers during the outbreak of the SARS epidemic in Hong Kong. J Epidemiol Community Health. 2004;58(12):988-96. Epub 2004/11/18. http://www.ncbi.nlm.nih.gov/pubmed/15547057.
24. Alexander D. NMA Condemns Non-Adherence To COVID-19 Prevention Protocols. 2020 [cited 2021 January]; Available from: https://consumerconnectng.com/8186.

25. Badillo-Goicoechea E, Chang T-HK, Esther, LaRocca S, Morris K, Deng X, Chiu S, et al. Global Trends and Predictors of Face Mask Usage During the COVID-19 Pandemic. arXiv API https://arxivorg/ftp/arxiv/papers/2012/201211678pdf. 2021.
26. Bo Y, Guo C, Lin C, Zeng Y, Li HB, Zhang Y, et al. Effectiveness of non-pharmaceutical interventions on COVID-19 transmission in 190 countries from 23 January to 13 April 2020. International journal of infectious diseases : IJID : official publication of the International Society for Infectious Diseases. 2021;102:247-53. Epub 2020/11/02. http://www.ncbi.nlm.nih.gov/pubmed/33129965.
27. Stutt R, Retkute R, Bradley M, Gilligan C, Colvin J. A modelling framework to assess the likely effectiveness of facemasks in combination with 'lock-down' in managing the COVID-19 pandemic. Proceedings of the Royal Scociety. A 476: 20200376. http://dx.doi.org/10.1098/rspa.2020.0376. 2020.
28. Jefferson T, Del Mar CB, Dooley L, Ferroni E, Al-Ansary LA, Bawazeer GA, et al. Physical interventions to interrupt or reduce the spread of respiratory viruses. The Cochrane database of systematic reviews. 2020;11:CD006207. Epub 2020/11/21. http://www.ncbi.nlm.nih.gov/pubmed/33215698.
29. Taylor S, Asmundson G. Negative attitudes about facemasks during the COVID-19 pandemic: The dual importance of perceived ineffectiveness and psychological reactance. PLoS One. 2021;16(2)):e0246317. https://doi.org/10.1371/journal.pone.
30. Rea E, Lafleche J, Stalker S, Guarda BK, Shapiro H, Johnson I, et al. Duration and distance of exposure are important predictors of transmission among community contacts of Ontario SARS cases. Epidemiology and infection. 2007;135(6):914-21. Epub 2007/01/16. http://www.ncbi.nlm.nih.gov/pubmed/17217552.
31. Dalton CB, Corbett SJ, Katelaris AL. COVID-19: implementing sustainable low cost physical distancing and enhanced hygiene. The Medical journal of Australia. 2020;212(10):443-6 e1. Epub 2020/05/02.
32. Global Times. Beijing districts, companies to enter into a state of emergency: official. 2020 [cited 2020 March]; Available from: https://www.globaltimes.cn/content/1211098.shtml.
33. Missoni E, Armocida B, Formenti B. Face Masks for All and All for Face Masks in the COVID-19 Pandemic: Community Level Production to Face the Global Shortage and Shorten the Epidemic. Disaster medicine and public health preparedness. 2020:1-5. https://doi.org/10.1017/dmp.2020.207.
34. Jefferson T, Del Mar C, Dooley L, Ferroni E, Al-Ansary LA, Bawazeer GA, et al. Physical interventions to interrupt or reduce the spread of respiratory viruses: systematic review. BMJ. 2009;339:b3675. Epub 2009/09/24.
35. Matusiak Ł, Szepietowska M, Krajewski PK, Białynicki-Birula R, zepietowski JC. The use of face masks during the COVID-19 pandemic in Poland: A survey study of 2315 young adults. Dermatologic therapy. 2020(e13909):Advanced online publication. https://doi.org/10.1111/dth.13909.
36. Van den Broucke S. Why health promotion matters to the COVID-19 pandemic, and vice versa. Health promotion international. 2020;35(2):181-6. Epub 2020/04/17. http://www.ncbi.nlm.nih.gov/pubmed/32297931.

37. Zhang L, Peng Z, Ou J, Zeng G, Fontaine RE, Liu M, et al. Protection by face masks against influenza A(H1N1)pdm09 virus on trans-Pacific passenger aircraft, 2009. Emerging infectious diseases. 2013;19(9). Epub 2013/08/24. http://www.ncbi.nlm.nih.gov/pubmed/23968983.

38. Shahnazi H, Ahmadi-Livani M, Pahlavanzadeh B, Rajabi A, Hamrah MS, Charkazi A. Assessing preventive health behaviors from COVID-19: a cross sectional study with health belief model in Golestan Province, Northern of Iran. Infectious diseases of poverty. 2020;9(1):157. Epub 2020/11/19. http://www.ncbi.nlm.nih.gov/pubmed/33203453.

Supporting information

S1 Study/Monitoring Protocol

S2 Data collection check list

S3 Data set in SPSS format

S4 IRB Approval

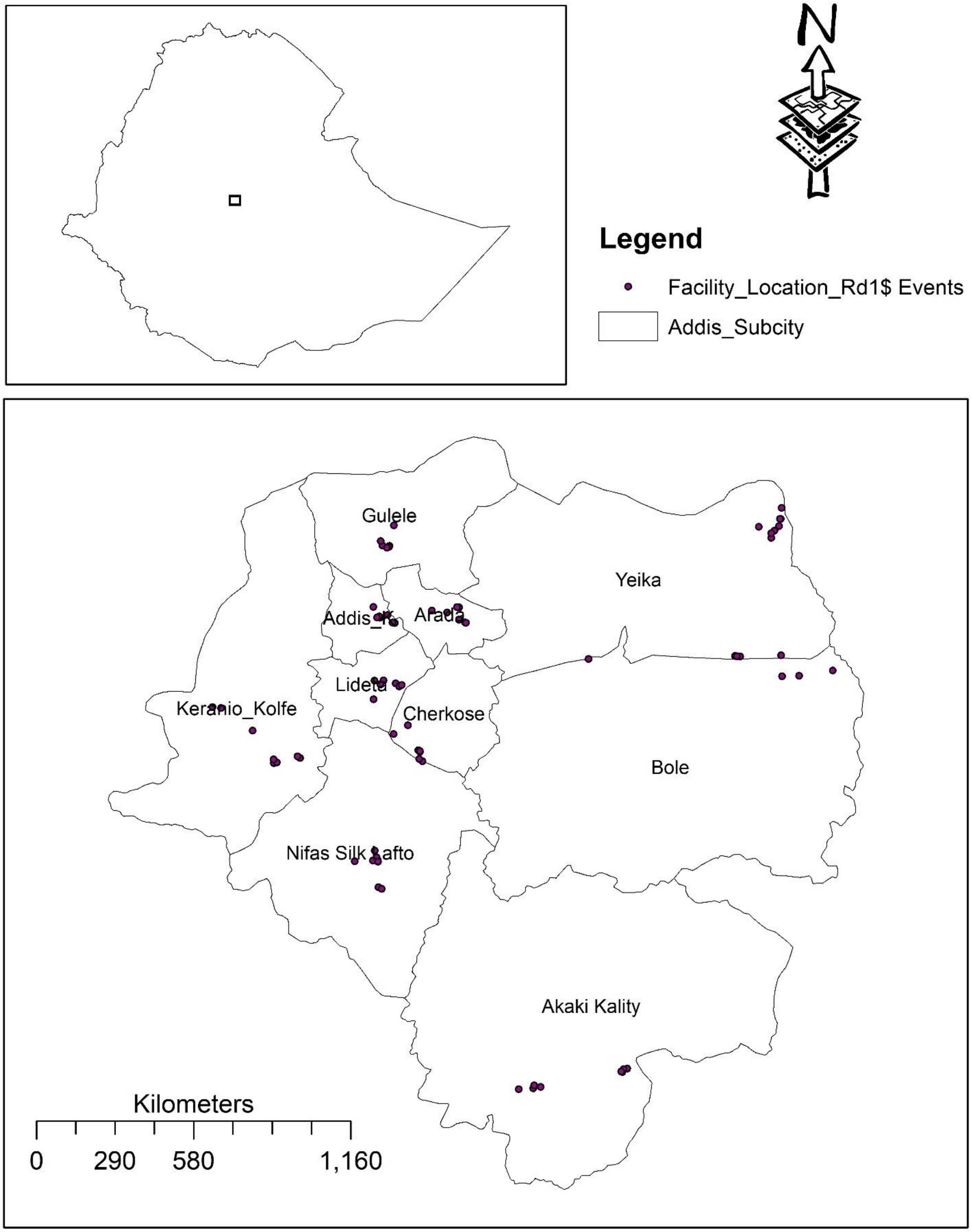

Figure 1: Observational data collection sites in Addis Ababa

Table 1: Number of people observed (April 20– June 14, 2020, Addis Ababa)

Insert Table 1

**Observation weeks**	**Weekdays observations # (%)**	**Weekend observations # (%)**	**Total observations # (%)**
Week 1 (Apr20-26)	225 (26.3)	629 (73.7)	854 (7.08)
Week 2 (Apr27-May3)	723 (62.1)	442 (37.9)	1165 (9.66)
Week 3 (May 4-May 10)	804 (63.6)	460 (36.4)	1264 (10.48)
Week 4 (May 11-May 17)	719 (60.0)	480 (40.0)	1199 (9.95)
Week 5 (May 20- May 24)	775 (62.2)	470 (37.8)	1245 (10.33)
Week 6 (May 27-May 31)	706 (59.1)	488 (40.9)	1194 (9.90)
Week 7 (June 3-June 7)	799(62.0)	489 (38.0)	1288 (10.68)
Week 8 (June 10-June 14)	788(61.7)	490 (38.3)	1278 (10.60)
Week 9 (June 15- June 21)	802 (62.6)	479 (37.4)	1281 (10.63)
Week 10 (June 22- June 28)	798 (62.0)	490 (38.0)	1288 (10.68)
**Total**	**7139 (59.2)**	**4917 (40.8)**	**12056 (100)**

Table 2: Demographic profile of participants by sub-city (April 20– June 14, 2020, Addis Ababa)

Variable	Total n (%) (n=12056)
Sex	
Male	6383 (52.9)
Female	5673 (47.1)
Age*	
<18 years	551 (4.6)
18-50 years	9832 (81.6)
>50 years	1673 (13.9)

*Age categories were made broad purposely in order to identify the closest age using observation without asking an individual ~~nd thus represent estimates~~and thus represent estimates

Table 3: Weekly pattern of community practice of non-pharmaceutical interventions (Addis Ababa, 2020)

NPI	NPI Practices	Week 1 (Apr20-26)	Week 2 (Apr27-May3)	Week 3 (May 4- 10)	Week 4 (May 11- 17)	Week 5 (May20- 24)	Week 6 (May 27-31)	Week 7 (June 3-7)	Week 8 (June10-14)	Week 9 (June15-21)	Week 10 June 22-28
Hand hygiene	**Proper hand hygiene**	**178 (24.2)**	**328 (33.1)**	**350 (32.5)**	**323 (32.0)**	**305 (28.8)**	**26 1 (25.9)**	**288 (26.1)**	**290 (26.8)**	**281 (28.5)**	**254 (23.7)**
	Improper hand hygiene	88 (11.9)	93 (9.4)	101 (9.4)	76 (7.5)	78 (7.4)	80 (7.9)	121 (10.9)	105 (9.7)	123 (12.4)	103 (9.6)
	No hand hygiene	469 (63.8)	571 (57.6)	625 (58.1)	610 (60.4)	674 (63.8	667 (66.2)	692 (62.8)	684 (63.4)	582 (59.1)	715 (66.7)
	**Total**	**735**	**992**	**1076**	**1009**	**1057**	**1008**	**1101**	**1079**	**986**	**1072**
Physical distance	**Proper physical distance**	**294 (34.4)**	**462 (39.7)**	**491 (38.8)**	**511 (42.6)**	**46 7 (37.5)**	**444 (37.2)**	**479 (37.2)**	**496 (38.8)**	**503 (39.3)**	**454 (35.2)**
	Improper physical distance	408 (47.8)	531 (45.6)	593 (46.9)	551 (46.0)	655 (52.6)	643 (53.9)	699 (54.3)	662 (51.8)	711 (55.5)	710(55.1)
	No physical distance	152 (17.8)	172 (14.8)	180 (14.2)	137 (11.4)	123 (9.9)	107 (9.0)	110 (8.5)	120 (9.4)	67 (5.2)	124(9.6)
	**Total**	**854**	**1165**	**1264**	**1199**	**1245**	**1194**	**1288**	**1278**	**1281**	**1288**
Respiratory hygiene	**Proper respiratory hygiene**	**205 (24.0)**	**301 (25.8)**	**405 (32.0)**	**575 (48.0)**	**746 (59.9)**	**845 (70.8)**	**985 (76.5)**	**980 (76.7)**	**999 (78.0)**	**995 (77.3)**
	Improper respiratory hygiene	79 (9.3)	174 (14.9)	213 (16.9)	271 (22.6)	151 (12.1)	153 (12.8)	198 (15.4)	214 (16.7)	208 (16.2)	201 (15.6)
	No respiratory hygiene	570 (66.7)	690 (59.2)	646 (51.1)	353 (29.4)	348 (28.0)	196 (16.4)	105 (8.2)	84 (6.6)	74 (5.8)	92 (7.1)
	**Total**	**854**	**1165**	**1264**	**1199**	**1245**	**1194**	**1288**	**1278**	**1281**	**1288**
Drivers' respiratory hygiene	**Proper respiratory hygiene**	**11 (13.8)**	**34 (23.0)**	**91 (55.2)**	**133 (78.7)**	**159 (84.6)**	**170 (89.5)**	**167(84.8)**	**165(82.9)**	**156(82.5)**	**168 (84.6)**
	Improper respiratory hygiene	1 (1.3)	11 (7.4)	15 (9.1)	11 (6.5)	23 (12.2)	17 (8.9)	29 (14.7)	30 (15.1)	30 (15.9)	27 (13.6)
	No respiratory hygiene	68 (85.0)	103 (69.6)	59 (35.8)	26 (15.3)	6 (3.2)	3 (1.6)	1 (0.5)	4 (2.0)	3 (1.6)	4 (2.0)
	**Total**	**80**	**148**	**165**	**170**	**188**	**190**	**197**	**199**	**189**	**199**
Driver assistants' respiratory hygiene	**Proper respiratory hygiene**	**0%**	**29 (19.6)**	**60 (37.3)**	**87 (52.1)**	**119 (62.6)**	**141 (70.5)**	**135 (69.9)**	**118 (60.5)**	**111 (59.0)**	**106 (54.1)**
	Improper respiratory hygiene	3(4.0)	19(12.8)	29 (18.0)	44 (26.3)	61 (32.1)	48 (24.0)	57 (29.5)	72 (36.9)	73 (38.8)	80 (40.8)
	No respiratory hygiene	72 (96.0)	100 (67.6)	72 (44.7)	36 (21.6)	10 (5.3)	11 (5.5)	1 (0.5)	5 (2.6)	4 (2.1)	10 (5.1)
	**Total**	**75**	**148**	**161**	**167**	**190**	**200**	**193**	**195**	**188**	**196**
Vehicle occupant capacity	**Less than or equal to 50% capacity**	**71 (88.8)**	**122 (83.0)**	**114 (69.1)**	**110 (65.1)**	**128 (68.1)**	**128 (67.4)**	**136(69.0)**	**140(70.4)**	**132(69.8)**	**137(68.8)**
	Greater than 50% capacity	9 (11.3)	25 (17.0)	51 (30.9)	59 (34.9)	60 (31.9)	62 (32.6)	61 (31.0)	59 (29.6)	57 (30.2)	62 (31.2)
	**Total**	**80**	**147**	**165**	**169**	**188**	**190**	**197**	**199**	**189**	**199**

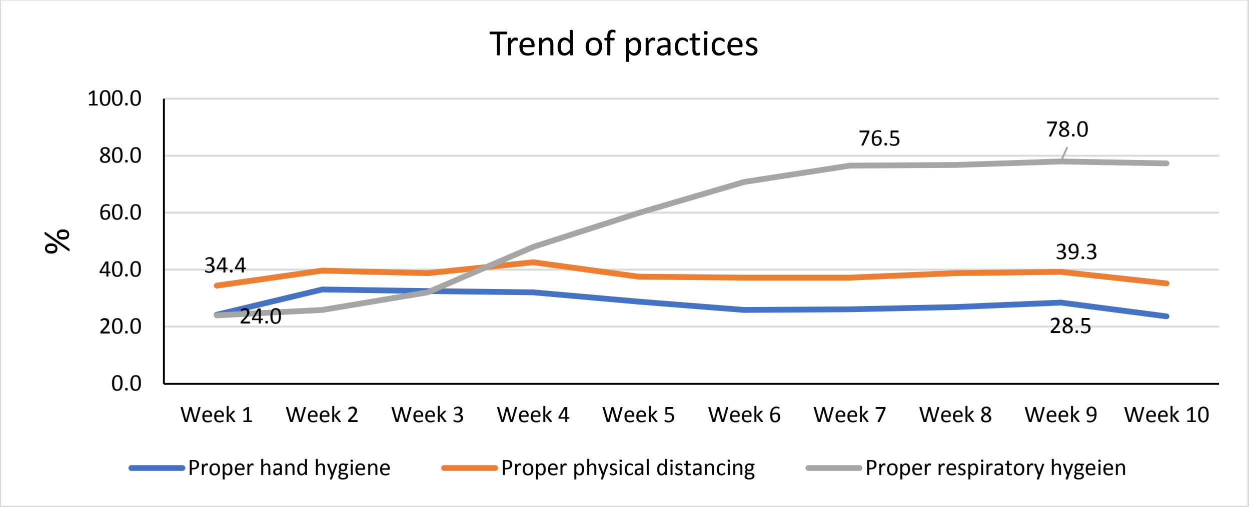

Figure 2: The overall trend of the proper community practices for the three
non-pharmaceutical interventions
~~universal precautions~~

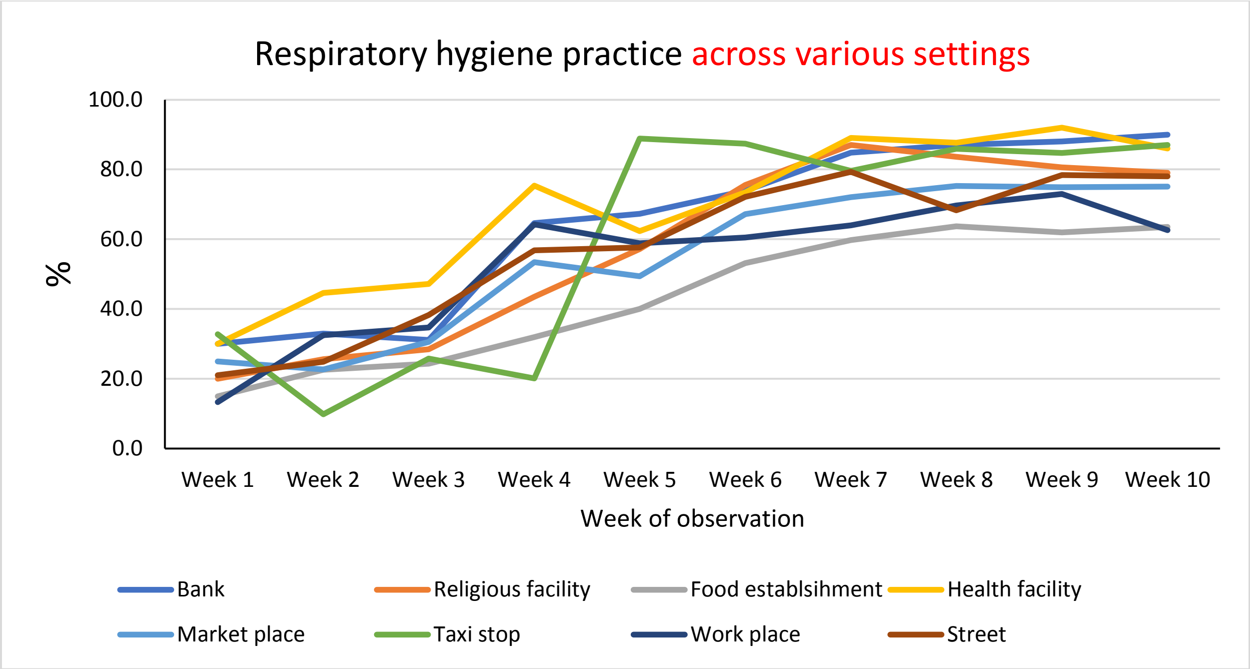

Figure 3: The trends of ~~the~~ proper respiratory hygiene practices at different institution sites

Table 4: Combined different~~s~~ community practices

Risk category	Risk classification	Week 1 (Apr 20-26)	Week 2 (Apr 27-May 3)	Week 3 (May 4-10)	Week 4 (May 11-17)	Week 5 May 20-24	Week 6 May 27-31	Week 7 June 3-7	Week 8 June10-14	Week 9 June15-21	Week 10 June22-28	Total
**Proper hand hygiene & distancing & respiratory**	Low risk	40 (4.7)	79(6.8)	96 (7.6)	130 (10.8)	99 (8.0)	95 (8.0)	98 (7.6)	123 (9.6)	113 (8.9)	100 (7.8)	973 (8.1)
**Proper distance & respiratory**	Low risk	97 (11.4)	150(12.9)	206 (16.3)	264 (22.0)	311 (25.0)	334 (28.0)	396 (30.7)	405 (31.7)	413 (32.2)	383 (29.7)	2959 (24.5)
**Proper hand hygiene & distancing**	Medium risk	91 (10.7)	172(14.8)	174 (13.8)	180 (15.0)	149 (12.0)	118 (9.9)	116 (9.0)	140 (11.0)	130 (10.3)	110 (8.6)	1380 (11.5)
**Proper hand and resp hygiene**	Medium risk	58 (6.8)	127(10.9)	163 (12.9)	205 (17.1)	183 (14.7)	193 (16.2)	237 (18.4)	250 (19.6)	240 (20.2)	227 (17.8)	1883 (15.8)
**Only mask**	High risk	73 (8.5)	68(5.8)	90 (7.1)	165 (13.8)	276 (22.2)	328 (27.5)	348 (27.0)	375 (29.3)	290 (24.1)	381 (29.8)	2394 (20.0)
**Only physical distancing**	High risk	130 (15.2)	183(15.7)	181 (14.3)	176 (14.7)	87 (7.0)	68 (5.7)	50 (3.9)	54 (4.2)	59 (4.6)	47 (3.7)	1035 (8.6)
**Only hand hygiene**	High risk	69 (8.1)	108(9.3)	109 (8.6)	68(5.7)	72(5.8)	45(3.8)	33(2.6)	23(1.8)	24(1.9)	17(1.3)	568 (4.7)
**No hand & respiratory hygiene & no distancing**	High risk	305 (35.7)	350 (30.0)	375 (29.7)	248 (20.7)	211 (16.9)	161 (13.5)	162 (12.6)	141 (11.0)	124 (9.8)	169 (13.1)	2246 (18.7)

*Risk classification was made based on the level of the three practices at an individual level. ~~It is a relative term: an~~ If an individual practiced~~in~~g all the three, he is grouped into ~~is in~~ a low risk category, while not practicing all the three or ~~none of~~ practicing only ~~doing hand hygiene and physical distance~~one of them is in high-risk group. An individual with two combination is in the medium risk.

Click here to access/download
Supporting Information
S2 Data Collection Checklist .docx

Click here to access/download
Supporting Information
S3 Data set SPSS.sav

Click here to access/download
Supporting Information
S4 IRB approval letter_08June2020 .pdf

Click here to access/download
Supporting Information
S1 Study protocol _Edited 31Aug2021.docx

Click here to access/download
Other
Rebuttal letter_04Sept 2021.docx

Research Article

Trends in non-pharmaceutical intervention (NPI) related community practice for the prevention of COVID-19 in Addis Ababa, Ethiopia

Authors List

Damen Haile Mariam[1], Abera Kumie[1*], Samson Wakuma[1], Yifoker Teferra[1], Teferi Abegaz[1], Worku Tefera[1], Wendimu Ayele[1], Mulugeta Tamire[1,] Shibabaw Yirsaw[1]

[1] School of Public Health, College of Health Sciences, Addis Ababa University

Corresponding Author

*aberakumie2@yahoo.com

Abstract

Background: The COVID-19 pandemic ~~had~~ affected Ethiopia since March 13, 2020, when the first case was detected in Addis Ababa. Since then, the incidence of cases has continued to increase day by day. As a result, the health sector has recommended universal preventive measures to be practiced by the public. However, studies on adherence to these preventive measures are limited~~ to inform evidence-based policy-making interventions~~.

Objective: To monitor the status of preventive practices of the population related to hand washing, physical distancing, and respiratory hygiene practices at selected sites within the city of Addis Ababa.

Methods: ~~A w~~Weekly cross-sectional non-participatory observation were~~was~~ done during the period of April-June, 2020. Data was collected using the Open Data Kit (ODK) tool in ten public sites involving eight public facilities targeted for individual observations. Ten individuals were randomly observed at each facility over two days a week at peak hours of public services. WHO operational definitions of the preventive behaviors were adopted for this study. Observations were anonymously ~~fixed~~ at gates or entrances of public facilities and places.

Results: A total of 12,056 individual observations with 53% males and 82% in an estimated age range of 18-50 years age group were involved in this study. There was an increase in the practice of respiratory hygiene ~~(~~from 14% in week one to 77% in week 10~~)~~, while those of hand hygiene and physical distancing changed little over the weeks from their baseline of 24% and 34%, respectively. Overall, respiratory hygiene ~~has shown~~demonstrated an increased rate of 6% per

week, while hand hygiene and physical distancing had ~~<1%~~ less than a 1% change per ~~-~~week, Females and the estimated age group of 18-50 years had ~~increased~~ practice in respiratory hygiene with no difference in hand hygiene and physical distancing. Respiratory hygiene took about six weeks to reach a level of 77% from its baseline of 24%, making an increment of about 9% per week.

Conclusion: The public practice of respiratory hygiene improved threefold whereas hand hygiene and physical distancing revealed no change. ~~was relatively better than hand hygiene and physical distancing~~. ~~It has shown visible weekly changes towards an increasing trend~~. Regularly sustained public mobilization and mass education are required to sustain the achievements gained in respiratory hygiene and further ~~improving~~ hand hygiene and physical distancing.

Keywords: COVID-19, hand hygiene, physical distancing, respiratory hygiene, mask use.

Introduction

The COVID-19 pandemic was originally described in is assumed to originate from Wuhan, China, in Dec 2019 (1). Since then, it has spread to Europe, the USA, and later to Africa. The infection's nature of transmission is considered to be shown to be through droplets, aerosols, and contact transmission (2). The possibility of transmission through faeco-oral and airborne routes is still under investigation (3, 4). Pre-symptomatic cases facilitate most of some of the the transmission for a household clustering of infection and during travels (5-7). Symptomatic infections appear to havewere having the highest transmission potential compared to asymptomatic and presymptomatic when considering the transmission index (8). This is in a way to say known COVID-19 cases with symptoms have the greatest transmission potential if left uncontrolled the source of transmission. While asymptomatic and pre-symptomatic cases involve unnoticed silent transmissions. The mortality pattern is devastating among elder age groups because of limited body immunity (9, 10).

Infection prevention and control (IPC) measures for emerging infectious diseases, like COVID-19, in health care settings can be managed with difficulty and confusionchallenging, especially in outbreaks where resources are limited (11). Yet such measures, including early identification, prompt isolation, proper patient placement, adequate space ventilation, and proper use of personal protective equipment, are important in preventing and controlling the transmission of COVID-19 (12). In addition, community prevention activities are considered the best strategies to reduce the healthcare burden, as they help slow transmission of the virus in the generala population (13). Community prevention strategies are a set of actions that persons and communities can implement

Formatted: Font: 12 pt

to slow the transmission of COVID-19 by practicing hand hygiene, respiratory hygiene, and social distancing (14).

The Ministry of Health of Ethiopia provides daily updates on the number of new infections and reported mortalities because of associated with COVID-19 (https://www.worldometers.info/coronavirus/country/ethiopia/). The first case in Ethiopia was declared on March 13, 2020, and the number of cases grew very slowly until the end of the first week of May 2020 (from 1 case on March 13 to 194 cases on 08 May 2020) (15). However, the number of reported cases increased since then, reaching 110,074 cases and 1,706 deaths on 01 December 2020 (the time of the write-up of this manuscript) (16).

In Ethiopia, the Federal Ministry of Health (FMOH) and the Ethiopian Public Health Institute have created awareness about the virus transmission and prevention methods using different approaches, including mass media such as national television and radionation TVs and radios during the partial-lock down period of five months since April, 20/2021. The interventions taken by the government ranged from banning public gatherings (over four persons) to limiting the number of passengers on public transport. In addition, all public service rendering institutions (such as food establishments, marketplaces, banks, and health facilities) provided hand-washing facilities and maintained a physical distance of at least one meter between their clients. Furthermore, security forces and public authorities have shown direct involvement in maintaining mask use and maintaining physical distancing keeping physical distance in public places such as marketplaces, public transport stations and terminals, and streets. Land borders were shut in an effort to control view of controlling the entrance of the virus from abroad. These interventions havehave been associated with proven the decline of COVID-19 transmission in other countries (17) and that

present the only effective methods ones in the absence of vaccines and medications. Data on monitoring the status of these interventions, however, is lacking in Ethiopia.

Our current study This monitoring generates evidence on COVID-19 prevention practices of the residents of Addis Ababa (focusing on hand hygiene, physical distancing, and respiratory hygiene). The findings are expected to inform policy to sustain the related behaviors in the general population in our country.

Methods

Study design, Study Area, Setting, and Period

A ~~weekly~~ cross-sectional observational design was conducted weekly between during April 24-June 28, 2020. Addis Ababa is the capital city of Ethiopia and is headquarters for the African Union and the Economic Commission for Africa. The city had a population of -5 million ~~has five mln~~ in Dec 2021 (18) and is sub-divided into ten administrative sub-cities. ~~Subcities~~Sub-cities are divided into districts ("Wereda"). One of the biggest markets in Africa ("*Merkato*") is located in the heart of the city. There are about 131 Orthodox churches as well as several mosques. About 67% of the total vehicles (estimated to be about 800,000) are registered within Addis Ababa. There are several public and private banks in the city – including the Commercial Bank of Ethiopia (a state enterprise) that alone has over 120 branches in Addis Ababa.

This study was initiated as part of the Emergency Operation Center (EOC) preventive behaviour monitoring activity at the College of Health Sciences (CHS), Addis Ababa University. CHS owns a teaching hospital that mandated~~required~~ an epidemic preparedness and management plan for its staff and visiting patients. The monitoring aimed to explore community practice levels regarding the three non-pharmaceutical interventions (NPI) for COVID-19 prevention including ~~(~~hand hygiene, physical distancing, and respiratory hygiene~~)~~, in the ten administrative units (sub-cities) of Addis Ababa. The study was~~has been~~ conducted by observing individual's behavior at selected sites when getting public services. These sites were selected based on the level of crowding and population mobility, and thus considered~~ing~~ an indication of the risk for COVID-19 transmission.

Study population

The source population for the study was all individuals visiting the selected sites during the day of data collection. Study participants were individuals who visited the selected sites during the time of data collection.

Monitoring protocol

Before the data collection, a monitoring protocol ~~has~~ hadbeen prepared and approved by the research committee which was ~~. This is~~ a standard operative procedure (SOP) used for the application of data collection. The protocol describe~~s~~d the observation site selection, identifie~~s~~d the list of public facilities to be observed, operationally define~~s~~d the NPI practices, and the time and the procedure for the observation. First, “community center-public sites' were identified in each sub-city. These sites are characterized by having increased population mobility relative to the nearby neighborhoods. Then, one of the relevant sites was identified as a nucleus for the subsequent selection of government and public institutions and facilities for monitoring. Eight facility-sites were purposely selected based on selection criteria that included active public service provision, availability of adequate flow of service users, and appropriateness of the location for observation relative to increased population mobility. These sites ~~are~~ represented religious places, health facilities, market places, banks, public transport, food and drink establishments, street crossing sites, and workplaces. Each public facility had ten observations.

We followed the protocol’s provision in standardizing the context of observation. Fixing the distance of fewer than 10 meters from the observation point represented by the gate or entrance to the public services (buildings or open spaces like a traditional market) was ~~kept~~maintained. This ~~is~~ represents the distance that maintains the proximity of observation by the research assistant.

The observation of an individual seeking a public service was designed anonymously to get valid behaviours of an individual's practice in reference to hand hygiene, physical distance, and respiratory hygiene. Ethical approval was obtained for this purpose. ~~No names or photos were required for observation.~~ The research assistant foundfinds a convenient place that allowed his/her visual field to be optimum for an observation. The observer ~~He~~ used a mobile phone ODK to record data without being identified by any person. This helps to distract suspecting his work, hence maintaining neutrality and data validity. ~~He~~ The observer kept moving within the proximity of the gate in case there might be any suspicion~~the discomfort~~ of being identified. The research assistant was instructed to spend about 15-30 minutes on one site, which ~~allowed him not to be identified easil~~prevented indentificationy. Finally, only two days in a week, weekdays (Wednesdays) and weekends (Sundays) were used for data collection to have weighted data represented by working days and holidays. The provisions of the context of monitoring are indicated in the support information (S1)

Data collection tools

We prepared a data collection tool that had two sub-sections (attached in a supplementary file). Section 1 addressed the location characteristics, while section 2 recordeds the practice of the individual. The location checklist addressed information regarding the time of observation, the Global Positioning System (GPS) of each service facility location, the presence of any enforcement, and the availability of handwashing facilities, including water and detergents. The NPI practice section contained sex, estimated age, NPI practice in three categories (proper, improper, and none). Each NPI practice was operationally defined within the monitoring protocol following the WHO recommendation (19) (S2).

Data collection procedure

One trained research assistant was assigned per sub-city, and one supervisor was appointed for two data collectors. The supervisor ~~used to communicate~~communicated with the field research assistant on each day of data collection to discuss the context and challenges of data collection. The field data were collected using the Open Data Kit tool (ODK) at the peak hours when the number of people was assumed to be highest at each observation site as stated in the protocol (Fig 1). The ~~daily~~ recorded ODK data were uploaded daily to the central server.

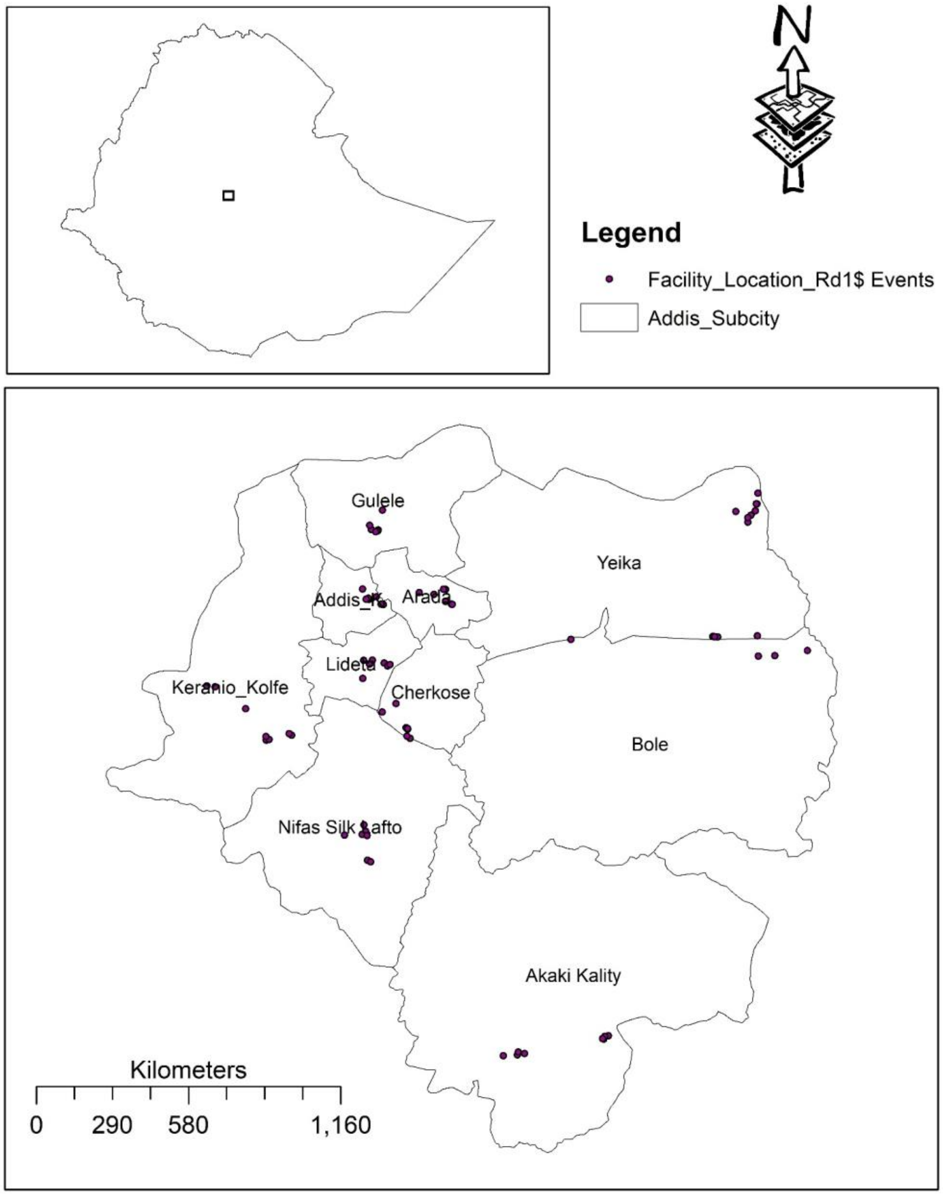

Figure 1: Observational data collection sites in Addis Ababa

Data management and analysis

Data from the main server were downloaded into Excel (Microsoft Corp.) in excel format and transferred into SPSS V 23 for data merging, cleaning, and analysis. Data were presented using descriptive statistics by calculating the proportion of practices for each week. The trend of the practice was evaluated using weekly differences in proportions (a current week with the previous week). The long-term trend was evaluated by visualizing the line graph and looking at the overall average of weekly differences. Limited statistical tests were done to see the significance of the overall practice with sex and the estimated age group. The weekly difference in the three practices was checked using a non-parametric test of Kruskal Wallis test. The average of differences between each week was considered to estimate the strength of the overall weekly change. Tables, line graphs, and charts were used to present data. The data set is in the supplemental support information (S3).

Ethical considerations

The study has received ethical approval from the Institutional Review Board (IRB) of the College of Health Sciences at Addis Ababa University. We had permission from IRB not to have consent as the results of the observations were meant to benefit the public at large and we did not seek any identifying information or photos of any individuals. identifiers and photos of an individual (S4). The survey useds a random observation tool that does not override the rights of any individual or institution, and the observation wasis done purely anonymously. A weekly report on the progress of monitoring was submitted to the Ministry of Health of Ethiopia to help to take proactive actions.

Results

Study participants

A total of 12,056 people were observed from April 20 to June 28, 2020. More than half of these (59.2%) were observed on the weekdays (Table 1). We had ~~males~~ 6,383 (52.9%) males and ~~females~~ 5,673 (47.1%) females in the study. The majority, 9832 (81.6%), were in the estimated age group of 18-50 years.

Table 1: Number of people observed (April 20– June 14, 2020, Addis Ababa)

Observation week	Weekdays observations # (%)	Weekend observations # (%)	Total observations # (%)
Week 1 (Apr20-26)	225 (26.3)	629 (73.7)	854 (7.1~~08~~)
Week 2 (Apr27-May3)	723 (62.1)	442 (37.9)	1,165 (9.7~~66~~)
Week 3 (May 4-May 10)	804 (63.6)	460 (36.4)	1264 (10.5~~48~~)
Week 4 (May 11-May 17)	719 (60.0)	480 (40.0)	1,199 (9.9~~5~~)
Week 5 (May 20- May 24)	775 (62.2)	470 (37.8)	1,245 (10.3~~3~~)
Week 6 (May 27-May 31)	706 (59.1)	488 (40.9)	1,194 (9.9~~0~~)
Week 7 (June 3-June 7)	799(62.0)	489 (38.0)	1,288 (10.7~~68~~)
Week 8 (June 10-June 14)	788(61.7)	490 (38.3)	1,278 (10.6~~0~~)
Week 9 (June 15- June 21)	802 (62.6)	479 (37.4)	1,281 (10.6~~3~~)
Week 10 (June 22- June 28)	798 (62.0)	490 (38.0)	1,288 (10.~~68~~7)
**Total**	**7,139 (59.2)**	**4,917 (40.8)**	**12,056 (100)**

Formatted Table

The overall trend of NPI community practice

Formatted: Font: 13 pt

Surgical masks predominated, (51%) relative to cloth masks, (41%), N95, (4%), and scarf, (4%). Proper hand hygiene in the community slowly increased from the baseline of 24.2% in week one to 32.5% in week three and then slowly declined to 23.7% in week 10. Similarly, proper physical distancing in the community increased from 34.4% in week one to 42.6 % in week four and then slowly declined to 35.2 % in week eight. On the contrary, proper respiratory hygiene in the community continuously increased from 14% in week one to 77% in week ten.

Driver's respiratory hygiene continuously increased from 13.8 % in week one to 84.6 % in week 10. Driver's assistants had 0% respiratory hygiene practice in week one but increased to 70.5% in week six, then slowly declined to 54.1% in week 10 (Table 2). The results also showed that adherence to the required vehicle occupancy less than or equal to 50% occupancy rate in commercial vehicles declined from 88.8% in week one to 68.8 % in week 10. There was a sharp rise in the practice of respiratory hygiene, while those of hand hygiene and physical distancing did not change or declined during the last week of observation (Fig 2). Week 4 was the turning point to observe an ~~increasing change~~increase, while the highest mask used in a population took seven weeks. The trend in hand hygiene and physically distancing through the ten weeks was stable relative to respiratory hygiene.

The three combined hand and respiratory hygiene practices with physical distancing managed by an individual were below 10% in any week. It increased slowly from 4.7% in week one to 10.8% in week four and then declined to 8.1% in week 10.

Varation in practices

Overall, we have found the within observations overtime statistical differences for the three practices ($p<0.05$), and within-subject differences by sex, age, and day of observation ($P<0.05$). Females and age category of 18-50 years had statistical differences in the relatively increased practice for respiratory hygiene. The practices observed in the week-days had an increased proportion over the week-end days ($p<0.05$) for hand and respiratory hygiene.

Formatted: Font: 13 pt

Table 2: Weekly pattern of a community practice of non-pharmaceutical interventions (Addis Ababa, 2020)

NPI	NPI Practices	Week 1 (Apr20-26)	Week 2 (Apr27-May3)	Week 3 (May 4-10)	Week 4 (May 11- 17)	Week 5 (May20- 24)	Week 6 (May 27-31)	Week 7 (June 3-7)	Week 8 (June10-14)	Week 9 (June15-21)	Week 10 June 22-28
Hand hygiene	**Proper hand hygiene**	**178 (24.2)**	**328 (33.1)**	**350 (32.5)**	**323 (32.0)**	**305 (28.8)**	**26 1 (25.9)**	**288 (26.1)**	**290 (26.8)**	**281 (28.5)**	**254 (23.7)**
	Improper hand hygiene	88 (11.9)	93 (9.4)	101 (9.4)	76 (7.5)	78 (7.4)	80 (7.9)	121 (10.9)	105 (9.7)	123 (12.4)	103 (9.6)
	No hand hygiene	469 (63.8)	571 (57.6)	625 (58.1)	610 (60.4)	674 (63.8	667 (66.2)	692 (62.8)	684 (63.4)	582 (59.1)	715 (66.7)
	**Total**	**735**	**992**	**1076**	**1009**	**1057**	**1008**	**1101**	**1079**	**986**	**1072**
Physical distance	**Proper physical distance**	**294 (34.4)**	**462 (39.7)**	**491 (38.8)**	**511 (42.6)**	**46 7 (37.5)**	**444 (37.2)**	**479 (37.2)**	**496 (38.8)**	**503 (39.3)**	**454 (35.2)**
	Improper physical distance	408 (47.8)	531 (45.6)	593 (46.9)	551 (46.0)	655 (52.6)	643 (53.9)	699 (54.3)	662 (51.8)	711 (55.5)	710(55.1)
	No physical distance	152 (17.8)	172 (14.8)	180 (14.2)	137 (11.4)	123 (9.9)	107 (9.0)	110 (8.5)	120 (9.4)	67 (5.2)	124(9.6)
	**Total**	**854**	**1165**	**1264**	**1199**	**1245**	**1194**	**1288**	**1278**	**1281**	**1288**
Respiratory hygiene	**Proper respiratory hygiene**	**205 (24.0)**	**301 (25.8)**	**405 (32.0)**	**575 (48.0)**	**746 (59.9)**	**845 (70.8)**	**985 (76.5)**	**980 (76.7)**	**999 (78.0)**	**995 (77.3)**
	Improper respiratory hygiene	79 (9.3)	174 (14.9)	213 (16.9)	271 (22.6)	151 (12.1)	153 (12.8)	198 (15.4)	214 (16.7)	208 (16.2)	201 (15.6)
	No respiratory hygiene	570 (66.7)	690 (59.2)	646 (51.1)	353 (29.4)	348 (28.0)	196 (16.4)	105 (8.2)	84 (6.6)	74 (5.8)	92 (7.1)
	**Total**	**854**	**1165**	**1264**	**1199**	**1245**	**1194**	**1288**	**1278**	**1281**	**1288**
Drivers' respiratory hygiene	**Proper respiratory hygiene**	**11 (13.8)**	**34 (23.0)**	**91 (55.2)**	**133 (78.7)**	**159 (84.6)**	**170 (89.5)**	**167(84.8)**	**165(82.9)**	**156(82.5)**	**168 (84.6)**
	Improper respiratory hygiene	1 (1.3)	11 (7.4)	15 (9.1)	11 (6.5)	23 (12.2)	17 (8.9)	29 (14.7)	30 (15.1)	30 (15.9)	27 (13.6)
	No respiratory hygiene	68 (85.0)	103 (69.6)	59 (35.8)	26 (15.3)	6 (3.2)	3 (1.6)	1 (0.5)	4 (2.0)	3 (1.6)	4 (2.0)
	**Total**	**80**	**148**	**165**	**170**	**188**	**190**	**197**	**199**	**189**	**199**
Driver assistants' respiratory hygiene	**Proper respiratory hygiene**	**0%**	**29 (19.6)**	**60 (37.3)**	**87 (52.1)**	**119 (62.6)**	**141 (70.5)**	**135 (69.9)**	**118 (60.5)**	**111 (59.0)**	**106 (54.1)**
	Improper respiratory hygiene	3(4.0)	19(12.8)	29 (18.0)	44 (26.3)	61 (32.1)	48 (24.0)	57 (29.5)	72 (36.9)	73 (38.8)	80 (40.8)
	No respiratory hygiene	72 (96.0)	100 (67.6)	72 (44.7)	36 (21.6)	10 (5.3)	11 (5.5)	1 (0.5)	5 (2.6)	4 (2.1)	10 (5.1)
	**Total**	**75**	**148**	**161**	**167**	**190**	**200**	**193**	**195**	**188**	**196**
Vehicle occupant capacity	**Less than or equal to 50% capacity**	**71 (88.8)**	**122 (83.0)**	**114 (69.1)**	**110 (65.1)**	**128 (68.1)**	**128 (67.4)**	**136(69.0)**	**140(70.4)**	**132(69.8)**	**137(68.8)**
	Greater than 50% capacity	9 (11.3)	25 (17.0)	51 (30.9)	59 (34.9)	60 (31.9)	62 (32.6)	61 (31.0)	59 (29.6)	57 (30.2)	62 (31.2)
	**Total**	**80**	**147**	**165**	**169**	**188**	**190**	**197**	**199**	**189**	**199**

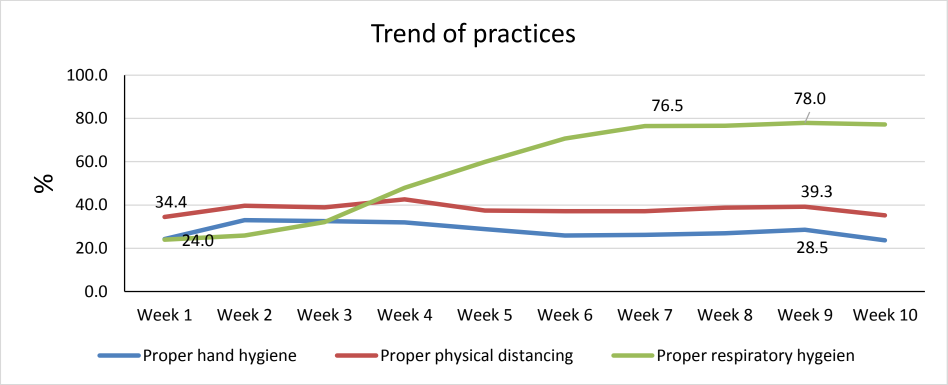

Figure 2: The overall trend of the proper community practices for the three ~~universal precautions~~NPIs

The trend of respiratory hygiene by observation facilities

There was a similar pattern of an increasing trend in all eight facilities with varying intensity of weekly increases~~ing~~. Banks and health facilities showed fast and stable growth rates over the ten weeks, from 30% in week one to 90% in week ten and from 30% in week 1 to 86% in week 10, respectively (Fig 3).

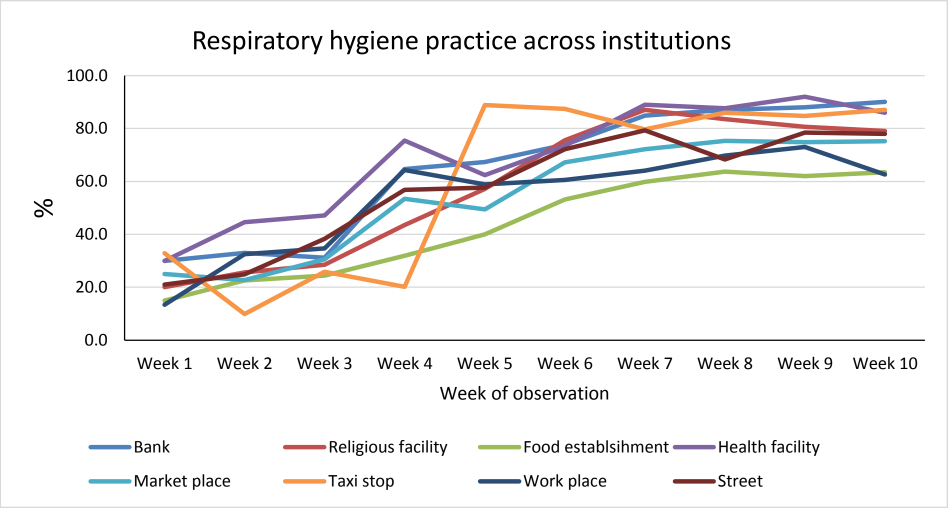

Figure 3: The trend of the proper respiratory hygiene practices by public facilities

Discussion

Non-pharmaceutical interventions are the primary preventions to restrict the spread of COVID-19 on the backgroundin the setting of limited access to vaccines. Overall, our results demonstrated that the use of masks was stable at around 80%, while hand hygiene and physical distancing could were not observed tonot go beyond 40%. The optimum application of the three practices was limited in our study and . This is only attained for the respiratory hygiene. The World Health Organization greatly acknowledged the combined practice of non-pharmaceutical interventions as a frontline tool to combat the infection (19, 20) .The non-pharmaceutical interventions were effective in containing the outbreak demonstrated by the number of cases that could had been 67-fold higher at the time without these interventions (20). Systematic reviews have shown the frequent practice of hand hygiene, the use of physical distancing of 1 m or more, and mask utilization have the a potential benefit of lowering the COVID-19 transmission (21, 22). These preventions interventions are effective with optimum coverage compliance if not universal in a community as has been strongly proposed in China for a high-risk population (23). The Nigerian Medical Association strongly condemned criticized the limited use of non-pharmaceutical prevention protocols by citizens (24). The low level of mask using was mainly attributed to risky social behaviors, perceived risk of transmission, gender and age differences (25). This study also showed the variation of the mask use over time across many countries: some having gradually increased, others showing a declining trend. The community's access to affordable masks in our study should have provided a sense of protection compared to hand hygiene and physical distancing. The use of cloth masks that areis easily accessible, cheap, and existing in many different options has provided a degree of protection next to surgical masks.

The present study has shown major improvement in the practice of respiratory hygiene compared to hand hygiene and physical distance during the period of our observation. The improvement started to show significant change after~~since~~ week five matched with when security forces started to enforce primary preventions in response to the state of emergency related to the epidemic crisis in Ethiopia. Although the state of emergency was declared on 20 April 2020, ~~its~~ the full breadth of ~~impact~~the impact came later, several weeks later of ~~just after few weeks that have been used for~~ massive promotional provisions targeting the public at large and public service rendering institutions in particular. At the time of the study, the State Emergency in Ethiopia was characterized to have partial lock-down of public places, including the closing of educational facilities and workplaces coupled with strict use of masks at public places, such as transport and public service places, which resulted in a rapid increase during our monitoring period. According to the state of emergency provisions, public mobility was not restricted to seeking food, transport, health care, and marketing services. There are reports from other countries that NPI interventions during lock-down periods indicating them ~~are effective~~as effective tools to interrupt COVID-19 transmission (26, 27), although the benefit of physical distanc~~e~~inge and masking has been uncertain ~~reported with uncertainties~~ due to ~~limited~~ lack of strong study designs (28). The state of emergency mandated private and government service providers to avail handwashing facilities with detergents for their clients who seek services. Handwashing facilities were~~are~~ usually placed at the gate or entrance of the institution to encourage hand hygiene before accessing the service. The perception of self-protections by using masks might have contributed towards the increasing practice. On the contrary, a small portion of the public, about 20%, did not use a mask that might be linked to the negative attitudes towards mask ineffectiveness to reduce the transmission (29).

Despite the progressed public enforcement, the practice of proper hand hygiene did not show much change over the ten weeks of observation and didcould not go beyond 30%. Two reasons were indicated suggested from our observation. Firstly, the media company and involvement of the enforcing volunteers had massive promotions in the first four weeks during our data collection period around churches, transport stations, and other public places. This has may have contributed to a change in handwashing practice in those weeks but not much beyond these weeks. On the other hand, the unavailability of water and soap could not be sustained in many facilities, except few public services providing institutions such as banks and health facilities. Generally, the provision of soap was initially challengingchallenged first, which was followed by the a shortage and total disappearance of water in many public service providers. We believe these challenges happened at the same time of weak enforcement when security and other partners discontinued the initiated interventions. There was a shift from washing hands to using hand sanitizer by some members of the population, especially the youth. Our observations result had limited coverage of the youth; thus, we might have underestimated the proportion of handwashing in a community. The youths had limited needs for seeking services in facilities that were involved in our study.

The maintenance of physical distancing at a minimum of one meter between any two individuals had a similar trend with handwashing practice. However, the former had an overall performance of below 40% relative to the 30% of the later practices. Physical distancing was challenged challenging for context-wise of social services, predominantly observed in open markets, walking and crossing on a street, and seeking public transport sectors. Individuals in an open market traditionally tend to cluster around a seller to make transactions. Seeking public transport services at peak hours makes service seekers get closer around the gate of transport vehicles. The duration

of exposure and physical distancing has been shown to be an important predictor of the transmission of SARS-CoV-1.~~COVID-19~~ (30). A systematic review of 172 studies reported that physical distancing of one meter was associated with a large reduction in the number of new infections in different countries (21). Taking the economic status of Ethiopia, the lack of change in the physical distancing at the public level in this study was a missed opportunity to avert the occurrence of new cases in the city~~ given~~. Hand hygiene and physical distancing are effective, low-cost interventions that could reduce~~be reducing~~ the number of new cases (17, 31). We believe limited adherence in hand hygiene and physical distancing are missed opportunities to avert new infections in a community.

Ethiopia possesses traditions that ~~enhance~~ encourage the community to get together, such as burial events, traditional religious festivals, attending churches, and traditional open markets. The State emergency was not universal in the restriction of mass mobility during its active implementation phase, which might be a factor for the spread of the infection. Clients were~~are~~ advised to perform the three practices strictly during the initial wave of the epidemic. The enforcement of NPIs ~~restriction~~ became weaker, however, a week after lifting off the state emergency. There were incidences ~~when~~ during the socio-cultural events ~~ignore~~ when physical distancing was ignored~~e~~ because of the population's crowding, such as religious holidays. ~~Inconsistent~~ Contrary with this finding, a study from China reported that almost all respondents avoided going to public places and reduced celebrating spring festivals due to the COVID-19 epidemic (32). The possible reasons for the difference in adherence might be associated with the socio-cultural contexts in a given country and the degree of enforcing preventive practices.

The extent and trend of mask utilization had visible differences compared to physical distancing and hand hygiene. Mask utilization has shownwas observed to have an increasing trend until it stabilizedds at about 80% towards the end of our study time. Many factors operated. The continued security enforcement, mass media mobilization, the restrictions of public services without a mask, and the provisions of regular information about COVID-19 using national TVs and radios were the main contributing factors. The multiple sources of information and public enforcement were believed to enhance the attitude of service seekers, which greatly modified their behavior towards the use of a mask. We cannot deny that access to multiple types of masks, such as surgical, N95, and cloth masks and , their local production at affordable prices has critically sustained the trend of mask utilization. The local production of face masks in low-middle income countries has been indicated as a strategy to contain the epidemic (33). Before or after the state of emergency, the time of the observation and the age of study subjects could make a difference in the proportion of mask using. There iswad a preference of using masks among the young population in Poland, making the figure at 60%, a study undertaken before the government's enforcement (34, 35), which is relatively lower than ours. The study in Poland was among the young population, which is not consistent with ours. Our study was undertaken after the declaration of State Emergency, which has brought multiple interventions to impact the behaviors of individuals intowards using a mask. Overall, the universal use of masks in public has been shown as an effective toolconsidered as a means to reduce the transmission of respiratory viruses COVID-19 in previous studies (36, 37).

We disaggregated the mask used by type of facilities to get insights about the factors affecting mask using. The trend of respiratory hygiene practice visibly increased among service providers involving health care facilities, banks, and transport services compared with other facilities

included in this study. There were assigned controllers and guards checking for the use of face-masks and maintenance of physical distancing and hand hygiene, who did not allow individuals to get a public service without these ~~proper~~ practices.

The relatively improved practices at bank and health facilities ~~are~~ may have been related to the fact that these institutions have committed the resources to provide handwashing facilities with detergents and that their clients would have ~~be~~ a relatively higher level of awareness and favourable attitude towards the preventive practices As there are variabilities in people's beliefs about the effectiveness as well as the rates of engagement in the use of non-pharmaceutical interventions, public health efforts should focus on increasing perceived severity and threats of the COVID-19 while promoting the preventive measures as effective tools (36, 38) and enforcing the use of them as necessary. On the contrary, the adherence to mask using and physical distancinge in open markets and food establishments will likely continue to exist as a challenge. Food and drink establishments require close attention because people eat in groups, and physical distancing was never~~almost not~~ practically applied. Onsite dining with indoor and outdoor seats was identified as a risky situation by the CDC (13).

~~This study has strengths because of~~A strength of our study was observing individuals randomly and anonymously at times of service provisions during peak hours in key social providing services. This approach ~~is believed to manage~~ would have reduced respondent's bias. The provision of weekly information to the Ministry of Health was an important input to enhance informed decisions to the public. We assumed observing individuals at the time of possible transmission in the presence of increased mobility and crowding provide~~s~~d better insights into the possibility of

the transmission potential. We recognize the limitations of our study. We could not determine ~~undermin~~e the presence of interview bias that could happen over time, although we had regular supervisions and meetings with research assistants. The data collected was for ~~representing only for~~ a limited time observation and may not ~~could not~~ represent the profile individual's behavior ~~in~~on a given day. We recognize the accuracy and reproducibility of age categories used are only estimates and variance was likely in this regard. Finally our study ~~This study~~ cannot predict the trends in community practice across the whole country in the absence of state wide interventions.

Conclusions and recommendations

Formatted: Font: 13 pt

Formatted: Heading 1

The overall community prevention practices for the of COVID-19 pandemic were very low in Addis Ababa, Ethiopia, although respiratory hygiene singly showed a relatively increased rate. Despite the relatively low adherence of the community to proper handwashing and proper physical distancing, the success of proper respiratory hygiene was encouraging. ~~We call to strengthen~~Strengthening social mobilization using available media and consistent enforcement mechanisms to get sustained performances in community practices would be of value.

Acknowledgments: The authors are grateful to the College of Health Sciences and the School of Public Health of Addis Ababa University for allowing this study to happen. The leadership of Dr Dawit Wendimagegn, who is the Chief ~~e~~Executive Director of the College of Health Sciences~~,~~ is greatly thanked for his support to undertake this study.

Competing interest: We do not have any competing financial interests or conflicts in this publication with any other person or institution.

Author's Contributions

DH tabled a research idea on the need to generate the status of community practice. AK was involved in the writing of the proposal and drafting the manuscript. AK and SW were involved in the analysis of data. MT was involved in handling the qualitative field information. WA was involved in the design of the data template in ODK. YT and TA were involved in the design of observation tools. SY was involved in coordinating the data collection. Finally, all were involved in editing the proposal and manuscript.

References

1. Chen X, Yu B. First two months of the 2019 Coronavirus Disease (COVID-19) epidemic in China: real-time surveillance and evaluation with a second derivative model. Global health research and policy. 2020;5:7. Epub 2020/03/12. https://doi.org/10.1186/s41256-020-00137-4.
2. Wold Health Organization. Modes of transmission of virus causing COVID-19: implications for IPC precaution recommendations. News and press release. 2021 [cited 2021 December]; Available from: https://reliefweb.int/report/world/modes-transmission-virus-causing-covid-19-implications-ipc-precaution-recommendations?gclid=Cj0KCQjwssyJBhDXARIsAK98ITRhh2Gzq9h-GPShFQYBpV0WJoAIKyXYgnx-RPb_KxnvDil199rRVgMaAuYTEALw_wcB.
3. Beattie RM, Ashton JJ, Penman ID. COVID-19 and the gastrointestinal tract: emerging clinical data. Frontline gastroenterology. 2020;11(4):290-2. Epub 2020/06/26. https://fg.bmj.com/content/flgastro/early/2020/04/27/flgastro--101507.full.pdf.
4. Liu J, Liao X, Qian S, Yuan J, Wang F, Liu Y, et al. Community Transmission of Severe Acute Respiratory Syndrome Coronavirus 2, Shenzhen, China, 2020. Emerging infectious diseases. 2020;26(6):1320-3. Epub 2020/03/04.
5. Guoqing Q, Naibin Y, Ada HYM, Liping W, Guoxiang L, Xueqin C, et al. COVID-19 Transmission Within a Family Cluster by Presymptomatic Carriers in China Clinical Infectious Diseases 2020;71(15):861-2. doi: 10.1093/cid/ciaa316.
6. Qiu X, Nergiz AI, Maraolo AE, Bogoch, II, Low N, Cevik M. The role of asymptomatic and pre-symptomatic infection in SARS-CoV-2 transmission-a living systematic review. Clinical microbiology and infection : the official publication of the European Society of Clinical Microbiology and Infectious Diseases. 2021;27(4):511-9. Epub 2021/01/24. http://www.ncbi.nlm.nih.gov/pubmed/33484843.
7. Yu X, Yang R. COVID-19 transmission through asymptomatic carriers is a challenge to containment. Influenza Other Respir Viruses. 2020;14(4):474–5. Epub Epub 2020. https://pubmed.ncbi.nlm.nih.gov/32246886/.
8. Koh WC, Naing L, Chaw L, Rosledzana MA, Alikhan MF, Jamaludin SA, et al. What do we know about SARS-CoV-2 transmission? A systematic review and meta-analysis of the secondary attack rate and associated risk factors. PLoS One. 2020;15(10):e0240205. Epub 2020/10/09.
9. Dudley JP, Lee NT. Disparities in Age-specific Morbidity and Mortality From SARS-CoV-2 in China and the Republic of Korea. Clinical infectious diseases : an official publication of the Infectious Diseases Society of America. 2020;71(15):863-5. Epub 2020/04/02. http://www.ncbi.nlm.nih.gov/pubmed/32232322.
10. Palaiodimos L, Kokkinidis DG, Li W, Karamanis D, Ognibene J, Arora S, et al. Severe obesity, increasing age and male sex are independently associated with worse in-hospital outcomes, and higher in-hospital mortality, in a cohort of patients with COVID-19 in the Bronx, New York. Metabolism. 2020;108:154262. Epub 2020/05/19. http://www.ncbi.nlm.nih.gov/pubmed/32422233.
11. World Health Organization. Infection prevention and control of epidemic- and pandemic-prone acute respiratory infections in health care. 2014 [cited 2020 April 2]; Available from: https://apps.who.int/iris/bitstream/handle/10665/112656/9789241507134_eng.pdf?sequence=1.
12. European Center for Diseases Prevention and Control. Infection prevention and control and preparedness for COVID-19 in healthcare settings. 2021 [cited 2020 April]; Available from: https://www.ecdc.europa.eu/sites/default/files/documents/Infection-prevention-control-for-the-care-of-patients-with-2019-nCoV-healthcare-settings_update-31-March-2020.pdf.
13. Centers for Diseases Control and Prevention. Implementation of Mitigation Strategies for Communities with Local COVID-19 Transmission. 2020 [cited 2020 April]; Available from: https://www.cdc.gov/coronavirus/2019-ncov/downloads/community-mitigation-strategy.pdf.

14. World Health Organization. Coronavirus disease (COVID-19) advice for the public. World Health Organization; 2019 [cited 2020 December]; Available from: https://www.who.int/emergencies/diseases/novel-coronavirus-2019/advice-for-public.
15. Worldometers. Ethiopia: Corona Virus Cases. 2020; Available from: https://www.worldometers.info/coronavirus/country/ethiopia/.
16. Pei S, Kandula S, Shaman J. Differential Effects of Intervention Timing on COVID-19 Spread in the United States. medRxiv : the preprint server for health sciences. 2020. Epub 2020/06/09. http://www.ncbi.nlm.nih.gov/pubmed/32511526.
17. Muller O, Neuhann F, Razum O. [Epidemiology and control of COVID-19]. Dtsch Med Wochenschr. 2020;145(10):670-4. Epub 2020/04/29. . Epidemiologie und Kontrollmassnahmen bei COVID-19. http://www.ncbi.nlm.nih.gov/pubmed/32344440.
18. United Nations. World Population Prospects. 2021; Available from: https://www.macrotrends.net/cities/20921/addis-ababa/population.
19. World Health Organization. Considerations for implementing and adjusting public health and social measures in the context of COVID-19. 2020; Available from: https://www.who.int/health-topics/coronavirus#tab=tab_2.
20. Lai S, Ruktanonchai NW, Zhou L, Prosper O, Luo W, Floyd JR, et al. Effect of non-pharmaceutical interventions to contain COVID-19 in China. Nature. 2020;585(7825):410-3. Epub 2020/05/05. http://www.ncbi.nlm.nih.gov/pubmed/32365354.
21. Chu DK, Akl EA, Duda S, Solo K, Yaacoub S, Schunemann HJ. Physical distancing, face masks, and eye protection to prevent person-to-person transmission of SARS-CoV-2 and COVID-19: a systematic review and meta-analysis. Lancet. 2020;395(10242):1973-87. Epub 2020/06/05. https://doi.org/10.5694/mja2.50602.
22. Jefferson T, Del Mar CB, Dooley L, Ferroni E, Al-Ansary LA, Bawazeer GA, et al. Physical interventions to interrupt or reduce the spread of respiratory viruses. The Cochrane database of systematic reviews. 2011(7):CD006207. Epub 2011/07/08. http://www.ncbi.nlm.nih.gov/pubmed/21735402.
23. Lau JT, Yang X, Tsui HY, Pang E. SARS related preventive and risk behaviours practised by Hong Kong-mainland China cross border travellers during the outbreak of the SARS epidemic in Hong Kong. J Epidemiol Community Health. 2004;58(12):988-96. Epub 2004/11/18. http://www.ncbi.nlm.nih.gov/pubmed/15547057.
24. Alexander D. NMA Condemns Non-Adherence To COVID-19 Prevention Protocols. 2020 [cited 2021 January]; Available from: https://consumerconnectng.com/8186.
25. Badillo-Goicoechea E, Chang T-HK, Esther, LaRocca S, Morris K, Deng X, Chiu S, et al. Global Trends and Predictors of Face Mask Usage During the COVID-19 Pandemic. arXiv API https://arxivorg/ftp/arxiv/papers/2012/201211678pdf. 2021.
26. Bo Y, Guo C, Lin C, Zeng Y, Li HB, Zhang Y, et al. Effectiveness of non-pharmaceutical interventions on COVID-19 transmission in 190 countries from 23 January to 13 April 2020. International journal of infectious diseases : IJID : official publication of the International Society for Infectious Diseases. 2021;102:247-53. Epub 2020/11/02. http://www.ncbi.nlm.nih.gov/pubmed/33129965.
27. Stutt R, Retkute R, Bradley M, Gilligan C, Colvin J. A modelling framework to assess the likely effectiveness of facemasks in combination with 'lock-down' in managing the COVID-19 pandemic. Proceedings of the Royal Scociety. A 476: 20200376. http://dx.doi.org/10.1098/rspa.2020.0376. 2020.
28. Jefferson T, Del Mar CB, Dooley L, Ferroni E, Al-Ansary LA, Bawazeer GA, et al. Physical interventions to interrupt or reduce the spread of respiratory viruses. The Cochrane database of systematic reviews. 2020;11:CD006207. Epub 2020/11/21. http://www.ncbi.nlm.nih.gov/pubmed/33215698.

29. Taylor S, Asmundson G. Negative attitudes about facemasks during the COVID-19 pandemic: The dual importance of perceived ineffectiveness and psychological reactance. PLoS One. 2021;16(2)):e0246317. https://doi.org/10.1371/journal.pone.
30. Rea E, Lafleche J, Stalker S, Guarda BK, Shapiro H, Johnson I, et al. Duration and distance of exposure are important predictors of transmission among community contacts of Ontario SARS cases. Epidemiology and infection. 2007;135(6):914-21. Epub 2007/01/16. http://www.ncbi.nlm.nih.gov/pubmed/17217552.
31. Dalton CB, Corbett SJ, Katelaris AL. COVID-19: implementing sustainable low cost physical distancing and enhanced hygiene. The Medical journal of Australia. 2020;212(10):443-6 e1. Epub 2020/05/02.
32. Global Times. Beijing districts, companies to enter into a state of emergency: official. 2020 [cited 2020 March]; Available from: https://www.globaltimes.cn/content/1211098.shtml.
33. Missoni E, Armocida B, Formenti B. Face Masks for All and All for Face Masks in the COVID-19 Pandemic: Community Level Production to Face the Global Shortage and Shorten the Epidemic. Disaster medicine and public health preparedness. 2020:1-5. https://doi.org/10.1017/dmp.2020.207.
34. Jefferson T, Del Mar C, Dooley L, Ferroni E, Al-Ansary LA, Bawazeer GA, et al. Physical interventions to interrupt or reduce the spread of respiratory viruses: systematic review. BMJ. 2009;339:b3675. Epub 2009/09/24.
35. Matusiak Ł, Szepietowska M, Krajewski PK, Białynicki-Birula R, zepietowski JC. The use of face masks during the COVID-19 pandemic in Poland: A survey study of 2315 young adults. Dermatologic therapy. 2020(e13909):Advanced online publication. https://doi.org/10.1111/dth.13909.
36. Van den Broucke S. Why health promotion matters to the COVID-19 pandemic, and vice versa. Health promotion international. 2020;35(2):181-6. Epub 2020/04/17. http://www.ncbi.nlm.nih.gov/pubmed/32297931.
37. Zhang L, Peng Z, Ou J, Zeng G, Fontaine RE, Liu M, et al. Protection by face masks against influenza A(H1N1)pdm09 virus on trans-Pacific passenger aircraft, 2009. Emerging infectious diseases. 2013;19(9). Epub 2013/08/24. http://www.ncbi.nlm.nih.gov/pubmed/23968983.
38. Shahnazi H, Ahmadi-Livani M, Pahlavanzadeh B, Rajabi A, Hamrah MS, Charkazi A. Assessing preventive health behaviors from COVID-19: a cross sectional study with health belief model in Golestan Province, Northern of Iran. Infectious diseases of poverty. 2020;9(1):157. Epub 2020/11/19. http://www.ncbi.nlm.nih.gov/pubmed/33203453.

Supporting information

S1 Study/Monitoring Protocol

S2 Data collection check list

S3 Data set in SPSS format

S4 IRB Approval

PONE-D-21-03031R1

Trends in non-pharmaceutical intervention (NPI) related community practice for the prevention of COVID-19 in Addis Ababa, Ethiopia

PLOS ON

31 Aug 2021

Response to Journal Academic Editor, John Conly, MD, PLOS ONE

Many thanks for the edits and for the overall guidance and encouragement

SN	Comments	Response
1	The English grammar and syntax are not at a standard which would be acceptable. I have taken the liberty of addressing the English grammatical errors throughout the manuscript and have placed the corrections in a series of strikethroughs and sticky notes.<br><br>Please review carefully. And make the changes in the Word version and ensure no scientific content has been altered.	First of all, many thanks for the liberty you did.<br><br>Apology for the language quality. We have done to our best using the free online Grammarly software. However, further editing required the payment for the business software support. We did not use this as our university has not that help.<br><br>We used the pdf file named. "PONE-D-21-03031_R1 Edits by Academic Editor" for editing between pdf and word file.

		All, the English grammar editing, strikeouts and stick notes were taken from the pdf to a word doc. This is well done accordingly
2	The references are in major need of "cleaning" to ensure they are in the appropriate format for the Journal with respect to abbreviations of the journal names, italics for Latin terms, case, punctuation, etc and must be completely correct.	References editing included<br>1. Avoiding any abbreviation for consistency<br>2. Now, many of them were downloaded from online sources using EndNote, which will help to capture the italics, punctuations as used to be<br>3. The reference style of PLOS ONE is now Vancouver.<br>4. The text citation Vs the Reference pages were checked for consistency. It was true some of the references were messy: repeating URL twice.<br>5. The EPUB and web link (URL) is included for the most of references.<br><br>Checking was made on:<br>1. International journal abbreviation<br>2. Vancouver<br>3. Italics checked<br>4. Dowload online as much as possible

3	The supplemental files have spelling errors, syntax errors and English grammatical errors and need to be carefully reviewed and appropriate corrections made.	The supplemental files were edited as per the comments.<br>This is mainly for the S1: Study/Monitoring Protocol<br>Others (S2-4) did not require any language checking as understood
