# Supplementary material for: Trends in non-pharmaceutical intervention (NPI) related community practice for the prevention of COVID-19 in Addis Ababa, Ethiopia
Source: PLoS One. 2021 Nov 23;16(11):e0259229. doi: 10.1371/journal.pone.0259229 (PMC8610281; doi:10.1371/journal.pone.0259229)
Supplement: Attachment — Submitted filename: Response to Academic Editor.docx [file pone.0259229.s006.docx]

PONE-D-21-03031
Trends in non-pharmaceutical intervention (NPI) related community practice for the prevention of COVID-19 in Addis Ababa, Ethiopia
PLOS ONE

30 June 2021

Response to Journal Academic Editor, John Conly, MD, PLOS ONE

| **SN** | **Comments** | **Response** |
| --- | --- | --- |
| 1 | Please address all the reviewer comments with the exception of Reviewer 2's comments about sample size given this is a descriptive epidemiologic study. | Response to each comment of the reviewer was mad in the text using a word track in word Review tool; It is also addressed in 3 files:   1. Response to Academic Editor 2. Response to Reviwer1 3. Response to Revier 2 |
| 2 | You can look at significance of differences between groups and provide some mean and median values where appropriate | We have indicated basic tests in the data analysis section and Results, Just basic ones in terms of overtime differences and within subject differences |
| 3 | Both reviewers commented about the details surrounding the NPIs and would agree more details are required other than a general WHO reference and the respiratory hygiene which is presumably referring to masks wearing needs to be defined more precisely. | This is an important concern. WHO has just given the definitions. We have customized that definition for a research purpose. The practice was categorized into Proper, not proper and not using.  Just an example:  Proper mask using: the mouth and nose is covered completely; Improper: there is a mask on the face, but either one of them or both were not covered. A mask under a chin is also improper;  Not used-if the mask is not at all seen in the face; if the mask is just handled by hands when it should be used.  All of this is indicated in the tool and a protocol that we have developed as SOP for field application.  The two docs are attached as a supplementary doc |
| 4 | The references to large amounts of transmission from asymptomatic cases needs  updating  (see  Koh WC, Naing L, Chaw L, Rosledzana MA, Alikhan MF, Jamaludin SA, Amin F, Omar A, Shazli A, Griffith M, Pastore R, Wong J.  What do we know about SARS-CoV-2 transmission? A systematic review and meta-analysis of the secondary attack rate and associated risk factors. PLoS One. 2020 Oct 8;15(10):e0240205) | Thanks for valid comment. I have used this reference on the symptomatic transmission risk; and a second reference to substantiate the presympromatic transmission risk.  What matters in all cases is managing the source of COVID-19. Known cases not using hygiene, physical distance, mask, not isolated will obviously transmit to the nearby population. However, pre-symptomatic and asymptomatic are silent transmitters as the patient himself does not recognize that he has COVID-19.  We appreciate the comment |
| 5 | You also need to refine what part of the WHO document to which you are referring in reference 14 and the Jefferson review listed as reference 23 has been updated to a recent new date from Nov 2020. | Thanks for this comment. We have updated the WHO reference with the title, “Considerations for implementing and adjusting public health and social measures in the context of COVID-19.  In addition, we have included updated reference Jefersson, T et al, Nov 2020, the uncertainties to physical distancing and mask using in the discussion |
| 6 | The Discussion is too long and unfocused in areas and needs to be tightened and agree with the comments of Reviewer 1 in this context. The references are not in the standard format for the Journal and case, formatting, access dates and full urls must be provided. | The discussion has multiple items. We have tried to focus, a bit in-depth. We could not cut the extent of discussion, however.  The reference format has now followed the PLOS One format. Used [} format. All Web pages have now URL. |
| 7 | You are strongly recommended to have someone whose native language is English review since there are many grammatical and syntax errors and it impacts negatively on the readability. | Thanks for comment. Do not blame us much, as our initial focus was to bring the science on a Table. We very much appreciate your gesture of looking at this relevant MS.  We have now edited aggressively using online checkers and some one among us who has an excellent English language |
| 8 | A rebuttal letter that responds to each point raised by the academic editor and reviewer(s). You should upload this letter as a separate file labeled 'Response to Reviewers'. | We have done this. |
| 9 | A marked-up copy of your manuscript that highlights changes made to the original version. You should upload this as a separate file labeled 'Revised Manuscript with Track Changes'.  An unmarked version of your revised paper without tracked changes. You should upload this as a separate file labeled 'Manuscript'. | Two revised versions are attached:   1. Tracked word file with edits 2. And cleaned one with no track changes |
| 10 | If applicable, we recommend that you deposit your laboratory protocols in protocols.io to enhance the reproducibility of your results. | What we mention a protocol in the manuscript is just a working guide or SOP for the application of data collection.  It is uploaded as a supplemental file, although adequate description of data collection was indicated in the Monitoring protocol section. |
| 11 | Tables and Figures formatting | Tables have now only heading and last raw lines  Fig 2 and 3 were a bit reformatted to relive the crowding |
|  | **Comments on Journal Requirements** |  |
| 1 | Please ensure that your manuscript meets PLOS ONE's style requirements, including those for file naming. The PLOS ONE style templates can be found at  https://journals.plos.org/plosone/s/file?id=wjVg/PLOSOne_formatting_sample_main_body.pdf and  [https://journals.plos.org/plosone/s/file?id=ba62/PLOSOne_formatting_sample_title_authors_affiliations.pdf](about:blank) | We have made citations in brackets using Plos One style. I had a bit difficulties, but made it  Many thanks for the link. The PLOS ONE text writing format guideline was used for both the abstract and body |
| 2 | 2. Please provide additional details regarding participant consent. In the ethics statement in the Methods and online submission information, please ensure that you have specified (a) whether consent was informed and (b) what type you obtained (for instance, written or verbal, and if verbal, how it was documented and witnessed). If your study included minors, state whether you obtained consent from parents or guardians. If the need for consent was waived by the ethics committee, please include this information. | Additional details were included in the ethical consideration section. We did not seek a consent and we had a permission from IRB for this. |
| 3 | 3. Thank you for stating the following in the Acknowledgments Section of your manuscript:  "The data collection was funded by the School of Public Health of Addis Ababa University. The Authors have contributed professionally in view of supporting the Ministry of Health in enhancing proactive informed decisions."  We note that you have provided funding information that is not currently declared in your Funding Statement. However, funding information should not appear in the Acknowledgments section or other areas of your manuscript. We will only publish funding information present in the Funding Statement section of the online submission form.  Please remove any funding-related text from the manuscript and let us know how you would like to update your Funding Statement. Currently, your Funding Statement reads as follows:  "The author(s) received no specific funding for this work."  Please include your amended statements within your cover letter; we will change the online submission form on your behalf. | We have now removed and agreed with your suggestion  We have modified the cover letter |
| 4 | Please amend the manuscript submission data (via Edit Submission) to include author Abera Kumie. |  |
| 5 | Please include your tables as part of your main manuscript and remove the individual files. Please note that supplementary tables (should remain/ be uploaded) as separate "supporting information" files | We have done this |
| 6 | Please include captions for your Supporting Information files at the end of your manuscript, and update any in-text citations to match accordingly. Please see our Supporting Information guidelines for more information: [http://journals.plos.org/plosone/s/supporting-information](about:blank). |  |
| 7 |  |  |
