# Supplementary material for: Trends in non-pharmaceutical intervention (NPI) related community practice for the prevention of COVID-19 in Addis Ababa, Ethiopia
Source: PLoS One. 2021 Nov 23;16(11):e0259229. doi: 10.1371/journal.pone.0259229 (PMC8610281; doi:10.1371/journal.pone.0259229)
Supplement: Attachment — Submitted filename: Response to Academic Editor_04 Sept 2021Use.docx [file pone.0259229.s008.docx]

PONE-D-21-03031R1
Trends in non-pharmaceutical intervention (NPI) related community practice for the prevention of COVID-19 in Addis Ababa, Ethiopia
PLOS ON

31 Aug 2021

Response to Journal Academic Editor, John Conly, MD, PLOS ONE

Many thanks for the edits and for the overall guidance and encouragement

| **SN** | **Comments** | **Response** |
| --- | --- | --- |
| 1 | The English grammar and syntax are not at a standard which would be acceptable. I have taken the liberty of addressing the English grammatical errors throughout the manuscript and have placed the corrections in a series of strikethroughs and sticky notes.  Please review carefully. And make the changes in the Word version and ensure no scientific content has been altered. | First of all, many thanks for the liberty you did.  Apology for the language quality. We have done to our best using the free online Grammarly software. However, further editing required the payment for the business software support. We did not use this as our university has not that help.  We used the pdf file named. “PONE-D-21-03031_R1 Edits by Academic Editor” for editing between pdf and word file.  All, the English grammar editing, strikeouts and stick notes were taken from the pdf to a word doc. This is well done accordingly |
| 2 | The references are in major need of "cleaning" to ensure they are in the appropriate format for the Journal with respect to abbreviations of the journal names, italics for Latin terms, case, punctuation, etc and must be completely correct. | References editing included   1. Avoiding any abbreviation for consistency 2. Now, many of them were downloaded from online sources using EndNote, which will help to capture the italics, punctuations as used to be 3. The reference style of PLOS ONE is now Vancouver. 4. The text citation Vs the Reference pages were checked for consistency. It was true some of the references were messy: repeating URL twice. 5. The EPUB and web link (URL) is included for the most of references.   Checking was made on:   1. International journal abbreviation 2. Vancouver 3. Italics checked 4. Dowload online as much as possible |
| 3 | The supplemental files have spelling errors, syntax errors and English grammatical errors and need to be carefully reviewed and appropriate corrections made. | The supplemental files were edited as per the comments. This is mainly for the S1: Study/Monitoring Protocol  Others (S2-4) did not require any language checking as understood |
