# Supplementary material for: Trends in non-pharmaceutical intervention (NPI) related community practice for the prevention of COVID-19 in Addis Ababa, Ethiopia
Source: PLoS One. 2021 Nov 23;16(11):e0259229. doi: 10.1371/journal.pone.0259229 (PMC8610281; doi:10.1371/journal.pone.0259229)
Supplement: Attachment — Submitted filename: Rebuttal letter_ONE-D-21-03031_R2_07Sept 2021.docx [file pone.0259229.s010.docx]

**Rebuttal letter**

| **Date:** | 07 October 2021 |
| --- | --- |
| **From:** | Abera Kumie, aberakum2@yahoo.com |
| **To:** | "PLOS ONE" [plosone@plos.org](mailto:plosone@plos.org) |
| **Subject** | **Edited publication files attached in the submission system** |

Attention:  [PONE-D-21-03031_R2 Ehtiopia editor edits.pdf](https://www.editorialmanager.com/pone/download.aspx?guid=%7ba39e3bff-ee0e-45ca-8ae3-dc703cba2d10%7d&scheme=13&id=448202)

Trends in non-pharmaceutical intervention (NPI) related community practice for the prevention of COVID-19 in Addis Ababa, Ethiopia
PLOS ONE

Dear Academic Edier

Given the comments we had on Sept 23/ 2021,

1. We have made edits and corrections according the edits of the Academic Editor using a file name: PONE-D-21-03031_R2 Ethiopia editor edits Academic Editor with Tracked edits. The language editing help was very helpful.
2. Reference was edited and corrected using EndNote link mainly with PubMed data base. Vancouver style was used based on the PloSOne reference guideline. The Journal titles were made in upper Case. However, some titles could not be made in this style: example is the Cochrane data base. Duplicate web link addresses were removed using URL
3. We did not make any other changes in the writing except for what was necessary as indicated by the Academic Editor.

We are now hoping to fulfill all the requirements of PolosOne., hoping its publication soon

Attached file

1. Rebuttal Letter
2. Word tracked cleaned Manuscript with edits
3. Word Cleaned Manuscript, edits removed

We very much thank the Academic Editor for the extended support in many aspects of edits.

Corresponding Author as Abera Kumie, [aberakum2@yahoo.com](mailto:aberakum2@yahoo.com)

Kind Regards
